# State of ex situ conservation of landrace groups of 25 major crops

**DOI:** 10.1038/s41477-022-01144-8

**Published:** 2022-05-09

**Authors:** Julian Ramirez-Villegas, Colin K. Khoury, Harold A. Achicanoy, Maria Victoria Diaz, Andres C. Mendez, Chrystian C. Sosa, Zakaria Kehel, Luigi Guarino, Michael Abberton, Jorrel Aunario, Bashir Al Awar, Juan Carlos Alarcon, Ahmed Amri, Noelle L. Anglin, Vania Azevedo, Khadija Aziz, Grace Lee Capilit, Oswaldo Chavez, Dmytro Chebotarov, Denise E. Costich, Daniel G. Debouck, David Ellis, Hamidou Falalou, Albert Fiu, Michel Edmond Ghanem, Peter Giovannini, Alphonse J. Goungoulou, Badara Gueye, Amal Ibn El Hobyb, Ramni Jamnadass, Chris S. Jones, Bienvenu Kpeki, Jae-Sung Lee, Kenneth L. McNally, Alice Muchugi, Marie-Noelle Ndjiondjop, Olaniyi Oyatomi, Thomas S. Payne, Senthil Ramachandran, Genoveva Rossel, Nicolas Roux, Max Ruas, Carolina Sansaloni, Julie Sardos, Tri Deri Setiyono, Marimagne Tchamba, Ines van den Houwe, J. Alejandro Velazquez, Ramaiah Venuprasad, Peter Wenzl, Mariana Yazbek, Cristian Zavala

**Affiliations:** 1 International Center for Tropical Agriculture (CIAT), Cali, Colombia; 2 CGIAR Research Program on Climate Change, Agriculture and Food Security (CCAFS), Cali, Colombia; 3 Wageningen University & Research (WUR), Plant Production Systems Group, Wageningen, The Netherlands; 4 San Diego Botanic Garden, Encinitas, CA USA; 5 Pontificia Universidad Javeriana Cali, Cali, Colombia; 6 Universidad del Quindío, Armenia, Colombia; 7 International Center for Agricultural Research in the Dry Areas (ICARDA), Rabat, Morocco; 8 Global Crop Diversity Trust, Bonn, Germany; 9 International Institute of Tropical Agriculture (IITA), Ibadan, Nigeria; 10 International Rice Research Institute (IRRI), Los Baños, Philippines; 11 International Center for Agricultural Research in the Dry Areas (ICARDA), Beirut, Lebanon; 12 International Maize and Wheat Improvement Center (CIMMYT), Texcoco, México; 13 International Potato Center (CIP), Lima, Peru; 14 United States Department of Agriculture (USDA), Agricultural Research Service, Aberdeen, ID USA; 15 International Crops Research Institute for the Semi-arid Tropics (ICRISAT), Hyderabad, India; 16 International Crops Research Institute for the Semi-arid Tropics (ICRISAT), Niamey, Niger; 17 Centre for Pacific Crops and Trees (CePaCT), Narere, Fiji; 18 Mohammed VI Polytechnic University (UM6P), Benguerir, Morocco; 19 Africa Rice Center (AfricaRice), Bouaké, Côte d’Ivoire; 20 World Agroforestry (ICRAF), Nairobi, Kenya; 21 International Livestock Research Institute (ILRI), Addis Ababa, Ethiopia; 22 Bioversity International, Montpellier, France; 23 Louisiana State University, Baton Rouge, LA USA; 24 Bioversity International, Leuven, Belgium

**Keywords:** Conservation biology, Climate-change adaptation

## Abstract

Crop landraces have unique local agroecological and societal functions and offer important genetic resources for plant breeding. Recognition of the value of landrace diversity and concern about its erosion on farms have led to sustained efforts to establish ex situ collections worldwide. The degree to which these efforts have succeeded in conserving landraces has not been comprehensively assessed. Here we modelled the potential distributions of eco-geographically distinguishable groups of landraces of 25 cereal, pulse and starchy root/tuber/fruit crops within their geographic regions of diversity. We then analysed the extent to which these landrace groups are represented in genebank collections, using geographic and ecological coverage metrics as a proxy for genetic diversity. We find that ex situ conservation of landrace groups is currently moderately comprehensive on average, with substantial variation among crops; a mean of 63% ± 12.6% of distributions is currently represented in genebanks. Breadfruit, bananas and plantains, lentils, common beans, chickpeas, barley and bread wheat landrace groups are among the most fully represented, whereas the largest conservation gaps persist for pearl millet, yams, finger millet, groundnut, potatoes and peas. Geographic regions prioritized for further collection of landrace groups for ex situ conservation include South Asia, the Mediterranean and West Asia, Mesoamerica, sub-Saharan Africa, the Andean mountains of South America and Central to East Asia. With further progress to fill these gaps, a high degree of representation of landrace group diversity in genebanks is feasible globally, thus fulfilling international targets for their ex situ conservation.

## Abstract (web summary)

By analysing the state of representation of traditional varieties of 25 major crops in ex situ repositories, this study demonstrates conservation progress made over more than a half-century and identifies the gaps remaining to be filled.

## Main

Crop landraces, also known as farmers’ traditional, heritage, folk or heirloom varieties, are cultivated plant populations developed and managed by Indigenous or traditional agrarian cultures through cultivation, selection and diffusion^1^. Having recognizable characteristics and geographic origins, landraces continue to be cultivated by these communities in many regions for their unique agroecological and societal functions and services^1,2^. These typically genetically heterogeneous populations are commonly planted in a mosaic of different crop species and varieties, in combinations sustaining local agricultural resilience and adaptive capacity, human nutrition and cultural needs^1,2^. Farmer-based exchange^3^ and gene flow among landrace populations, occasionally also involving modern cultivars^4^ or wild progenitors^5^, encourage the development of new variation, while longstanding cultivation and selection lead to adaptation to local environmental and societal conditions^6^.

Landrace diversity is an essential genetic resource for modern crop breeding^7^ and is key to understanding agricultural origins and domestication processes^8^. Landraces are typically accessed via ex situ repositories, called genebanks, for these research purposes. Efforts to collect landraces for genebank conservation have often prioritized sampling from geographic regions and cultures wherein crops were domesticated and/or have been cultivated for a very long time, in recognition of the extraordinary genetic variation in landraces found in these environments^1,7,9^. These activities have gained urgency since the 1960s as economic, agricultural, demographic, environmental and climatic changes increasingly impact in situ populations^1,7^. The result of these collection efforts has been the assemblage of approximately three million landrace samples in international, regional, national and subnational genebanks^10^.

Despite these extensive efforts, landrace diversity is not commonly considered to be comprehensively represented ex situ, and major international agreements, including the Convention on Biological Diversity (CBD) Aichi Target 13 (ref. ^11^) and the Sustainable Development Goals (SDGs) Target 2.5 (ref. ^12^), urgently prioritize the resolution of this conservation gap. To reach these targets, information about the current distributions of landraces and their degree of representation in genebanks is needed. To respond to this need, in this Article we employ a conservation gap analysis methodology^13^ to predict the distributions and quantify the current ex situ conservation status of 71 eco-geographically distinguishable groups of landraces within 25 cereal, pulse and starchy root/tuber/fruit crops whose genetic resources are researched and conserved by CGIAR international agricultural research centres or by the Centre for Pacific Crops and Trees (CePaCT) of the Pacific Community (SPC). We identify gaps in existing ex situ collections to inform further collecting efforts.

## Results

### Geographic distributions of crop landrace groups

**Fig. 1:**
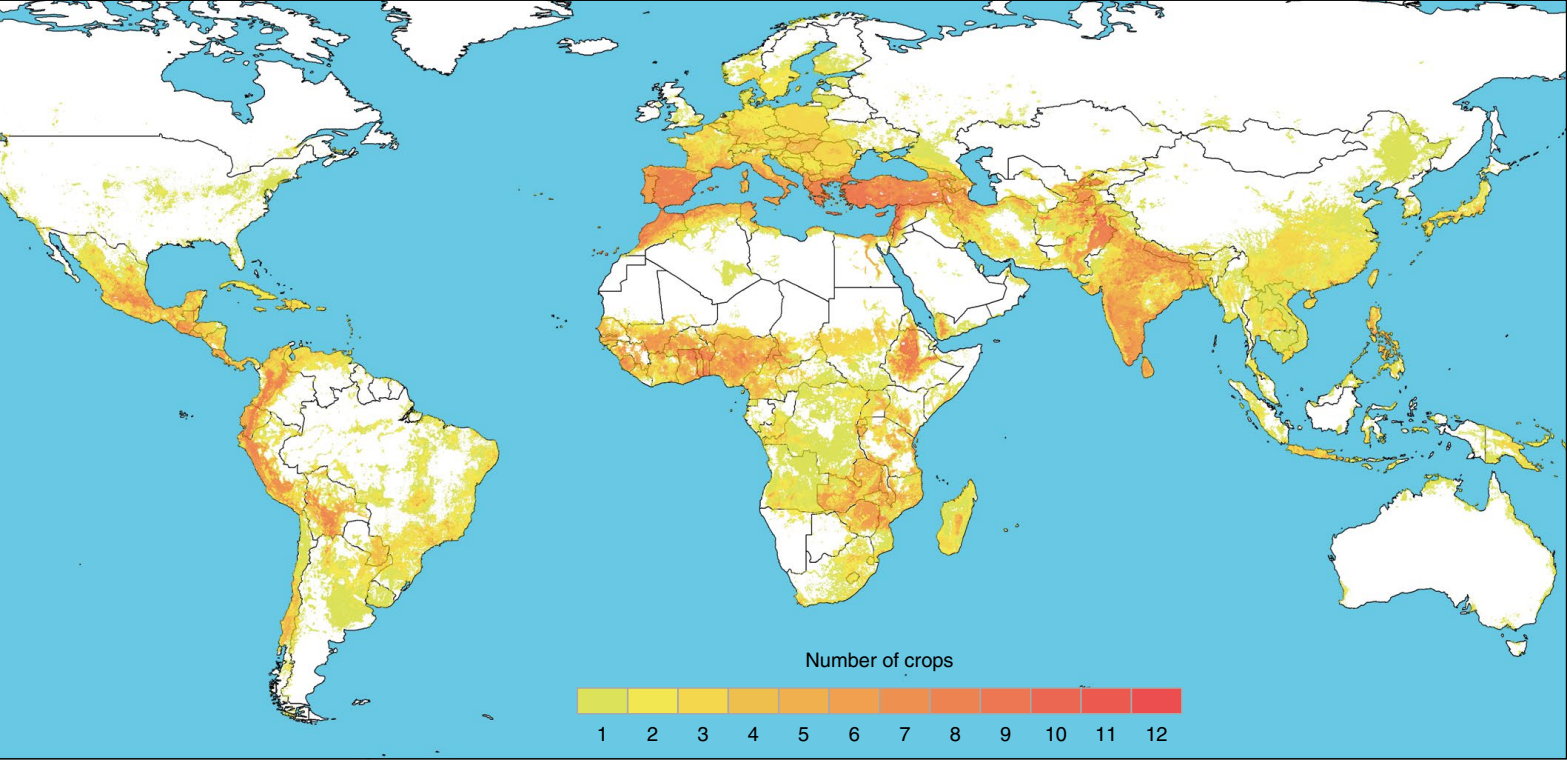
Richness map of the predicted distributions of landrace groups of 25 cereal, pulse and starchy root/tuber/fruit crops within their geographic regions of diversity. Darker colours indicate greater numbers of crop landrace groups potentially overlapping in the same 2.5-arc-minute cells, quantified in terms of number of crops. See Extended Data Fig. 1 for richness across all 71 landrace groups within the 25 crops. Source data

**Extended Data Fig. 1:**
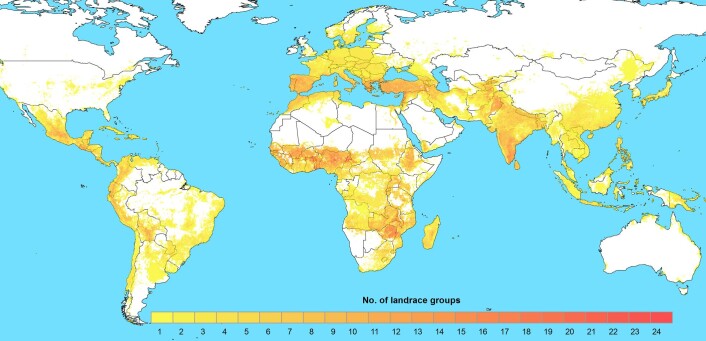
Richness map of the predicted distributions of 71 landrace groups of 25 cereal, pulse, and starchy root/tuber/fruit crops within their geographic regions of diversity. Richness map of the predicted distributions of 71 landrace groups of 25 cereal, pulse, and starchy root/tuber/fruit crops within their geographic regions of diversity. Darker colors indicate greater numbers of crop landrace groups potentially overlapping in the same 2.5 arc-minute cells. Source data

On the basis of correlations among 93,269 landrace occurrences of 25 crops (61.9% of occurrences having pre-assigned landrace group assignments and the rest inferred) and 50 environmental and socioeconomic predictor variables, landraces as a whole were predicted to be distributed on all inhabited continents, including throughout most of the world’s tropical and subtropical lands (Fig. 1 and Extended Data Fig. 1). Regions with particularly high levels of richness across crops were projected in East and Southern Africa, South and Central Asia, the Mediterranean and West Asia, West Africa and the Andean mountains of South America and Mesoamerica, with landraces of up to 12 of the 25 crops potentially cultivated within single 2.5-arc-minute grid cells in Bangladesh, Ethiopia, India, Nepal and Pakistan. These geographic concentrations of landrace group diversity align well with the historically recognized centres of origin and primary regions of diversity of the world’s major crops^14,15^. Notably less landrace diversity across crops was predicted to be cultivated in most temperate regions, in some very arid zones such as the Saharan Desert and in a few highly mesic areas such as the Amazon Basin.

**Fig. 2:**
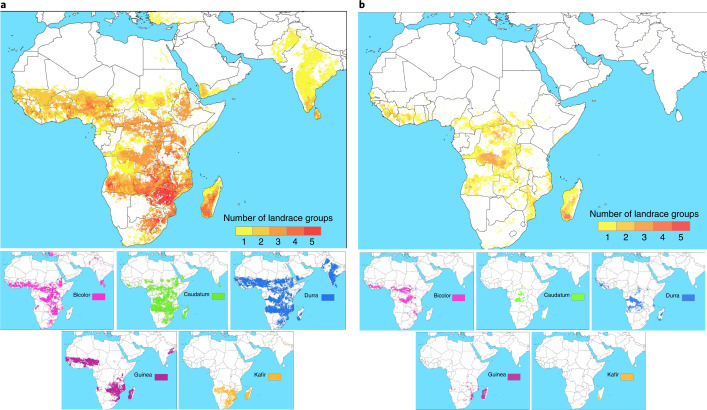
Richness maps of sorghum landrace group distributions and ex situ conservation gaps. **a**,**b**, Predicted distributions (**a**) and ex situ conservation gaps (**b**) for five landrace groups of sorghum in Africa, South Asia, the Mediterranean, and West Asia—namely, the races bicolor, caudatum, durra, guinea and kafir. Small maps, individual distributions of each landrace group; large maps, richness at the crop level. Source data

**Extended Data Fig. 2:**
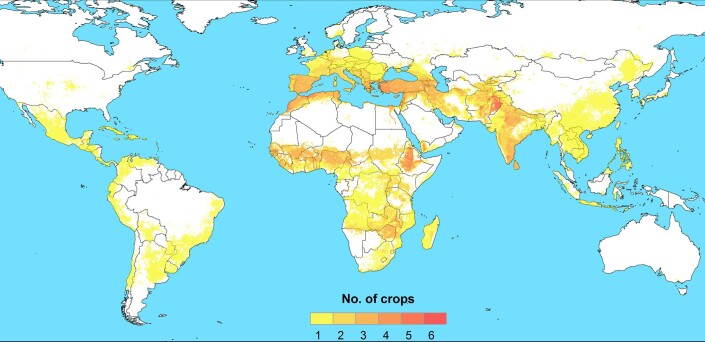
Richness map of the predicted distributions of landrace groups of 9 cereal crops within their geographic regions of diversity. Richness map of the predicted distributions of landrace groups of 9 cereal crops within their geographic regions of diversity. Darker colors indicate greater numbers of crop landraces potentially overlapping in the same 2.5 arc-minute cells, quantified in terms of numbers of crops. Source data

**Extended Data Fig. 3:**
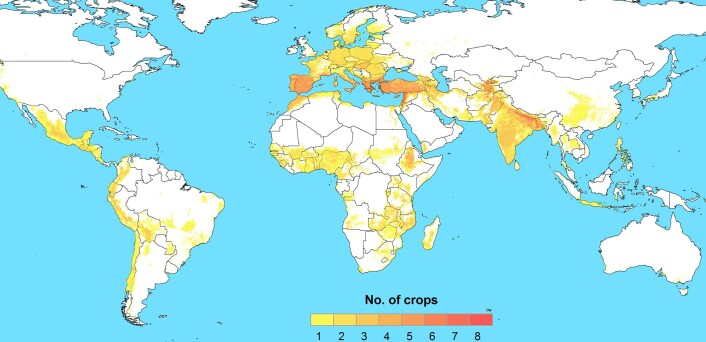
Richness map of the predicted distributions of landrace groups of 9 pulse crops within their geographic regions of diversity. Richness map of the predicted distributions of landrace groups of 9 pulse crops within their geographic regions of diversity. Darker colors indicate greater numbers of crop landraces potentially overlapping in the same 2.5 arc-minute cells, quantified in terms of numbers of crops. Source data

**Extended Data Fig. 4:**
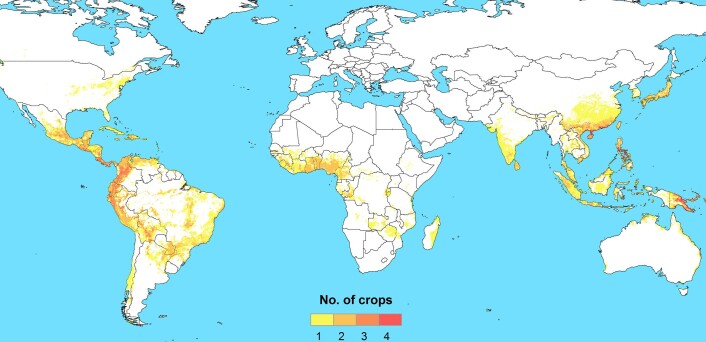
Richness map of the predicted distributions of landrace groups of 7 starchy root, tuber, and fruit crops within their geographic regions of diversity. Richness map of the predicted distributions of landrace groups of 7 starchy root, tuber, and fruit crops within their geographic regions of diversity. Darker colors indicate greater numbers of crop landraces potentially overlapping in the same 2.5 arc-minute cells, quantified in terms of numbers of crops. Source data

The predicted distributions of the five major races of sorghum are provided in Fig. 2a as an example of landrace-group-level results (the Supplementary Information presents the occurrences and predicted distributions of landrace groups for all assessed crops). Sorghum landrace group ranges were modelled throughout the crop’s main regions of diversity in Africa, South Asia, the Mediterranean and West Asia. Its races inhabit distinct eco-geographic ranges but also overlap in specific areas, particularly in Southern and West Africa and in South Asia. Regarding different types of assessed crops, cereal and pulse landrace group diversity was predicted to be particularly rich in South and Central Asia; West, East and Southern Africa; the Mediterranean and West Asia; Europe; the Andean mountains; and Mesoamerica. Meanwhile, starchy root/tuber/fruit crop landrace group richness was concentrated in Mesoamerica, Southeast Asia and the Pacific, South America, West Africa and South Asia (Extended Data Figs. 2–4).

### Ex situ conservation status and gaps for crop landrace groups

**Fig. 3:**
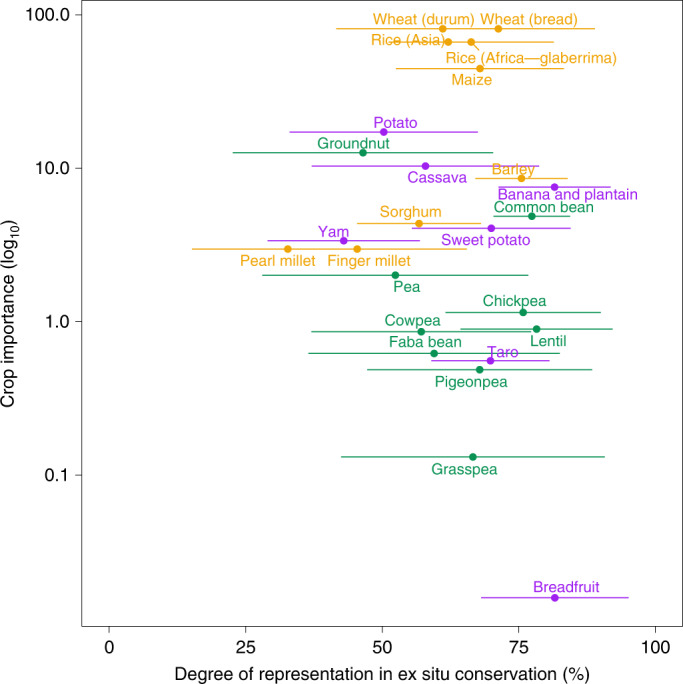
The current representation of crop landrace groups in ex situ conservation. Conservation metrics provide a scale from the lower to the upper estimates of current ex situ conservation status per crop with the averages denoted by circles. The crop importance metric indicates the current significance of the crop, averaged across global food supply, production and trade metrics (Supplementary Information). Gold, cereals; green, pulses; purple, starchy roots/tubers/fruits. Source data

On average, ex situ conservation of crop landrace groups—measured in terms of the extent of current cultivated geographic range and ecological variation in the range that has previously been collected from and is now conserved in genebanks—was estimated to be moderately comprehensive at present, with substantial variation among crops; an average of 63% ± 12.6% of distributions was represented ex situ (Fig. 3 and Supplementary Table 1). Measured as the mean of the estimated minimum and maximum extent of representation in genebanks, geographic and ecological variation in landrace groups of the following crops was among the most comprehensively represented: breadfruit at 81.6% conserved, bananas and plantains at 81.5%, lentils at 78.3%, common beans at 77.4%, chickpeas at 75.8%, barley at 75.5% and bread wheat at 71.3%. Conversely, the largest conservation gaps persist for pearl millet at 32.7%, yams at 43.0%, finger millet at 45.4%, groundnut at 46.5%, potatoes at 50.3% and peas at 52.4%. The maximum potential representation metrics indicate that breadfruit, lentil, banana and plantain, grasspea and chickpea landrace group variation may already be very well conserved, since all have maximum current ex situ conservation scores above 90%, while the minimum coverage metrics warn that some crops may still face extensive conservation gaps, such as pearl millet at 15.2%, groundnut at 22.6%, finger millet at 25.3%, peas at 28.1% and yams at 29.0%.

Regarding types of assessed crops, the average degree of ex situ conservation of cereal, pulse and starchy root/tuber/fruit landrace groups did not differ significantly (*P* = 0.69), measuring 59.9%, 64.6% and 64.9%, respectively. At 45.0%, 45.6% and 50.4%, their mean minimum potential representation values were also similar, as were their maximum potential representation values of 74.8%, 83.6% and 79.3%. For the final crop-type category, this finding is remarkable because these plants are represented by lower overall numbers of genebank samples (an average of 1,052.7 accessions versus 2,827.4 of pulses and 5,796.4 of cereals); these typically clonally propagated crops often require higher ex situ conservation expenditures per sample and present more substantial challenges from pests and diseases^16^. Nonetheless, cereal, pulse and starchy root/tuber/fruit crop types all included members with some of the least and most comprehensive conservation scores (Fig. 3). Moreover, these scores were not correlated with importance of the crop to global food supplies, production and trade (*r* = 0.064)

At the landrace group level, considerable differences in current conservation status were identified among groups within many crops (Supplementary Table 2). For example, geographic and ecological variation in barley landraces with covered (with hull) grains was estimated to be 89.1% conserved, while diversity in landraces with naked or hull-less grains was only 31.3% represented ex situ. Asian rice, finger millet, potato, sorghum and yam landrace groups also varied rather widely regarding current conservation in genebanks, while cassava, chickpea, common bean, cowpea, groundnut, lentil, maize, pea, pearl millet, African rice, sweetpotato and bread wheat landrace groups had more similar within-crop ex situ representation estimates.

Taking sorghum landrace groups as an example, high-confidence gaps in current ex situ conservation in terms of geographic and ecological variation were identified for all five major races in sub-Saharan Africa, with overlapping gaps concentrated in Central, West and Southern Africa, including in Madagascar (Fig. 2b). The Supplementary Information provides conservation gap maps for all assessed crops.

**Fig. 4:**
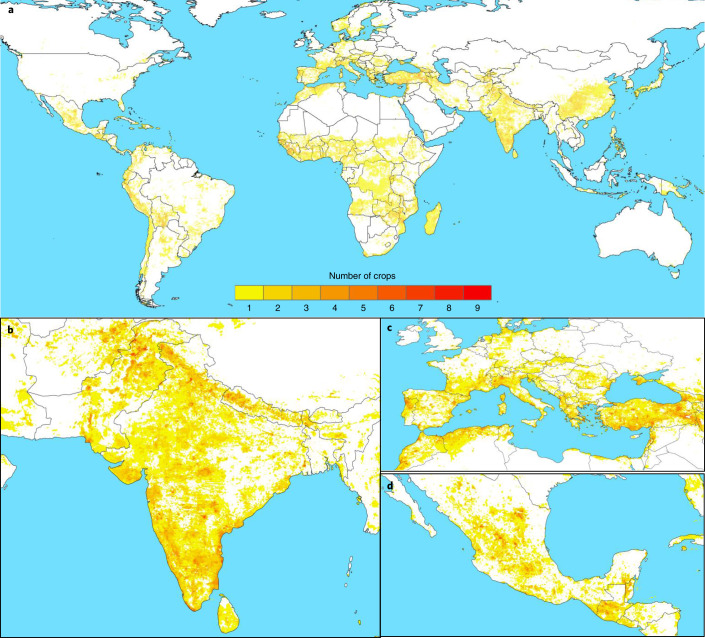
Geographic hotspots for further collection for the ex situ conservation of crop landrace groups. **a**, Global map of ‘gap richness’ across the predicted distributions of landrace groups of 25 cereal, pulse and starchy root/tuber/fruit crops within their geographic regions of diversity, indicating where landraces are expected to occur and have not yet been collected and conserved in genebanks. Darker colours indicate greater numbers of uncollected crop landrace groups potentially overlapping in the same 2.5-arc-minute cells, quantified in terms of numbers of crops. **b**–**d**, Examples of regions with particularly high gap richness in South Asia (**b**), the Mediterranean and West Asia (**c**) and Mesoamerica (**d**). See Extended Data Fig. 5 for gap richness across the 71 landrace groups within the 25 crops. Source data

**Extended Data Fig. 5:**
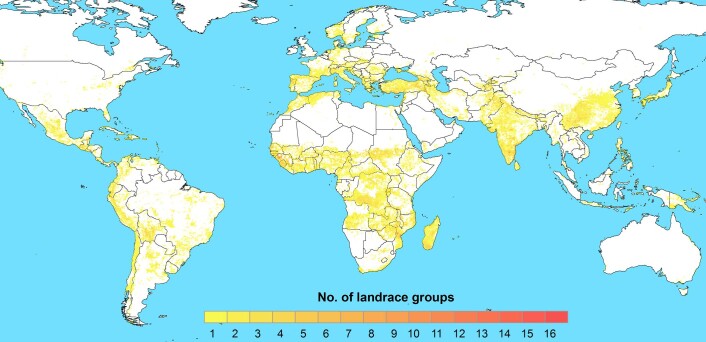
Geographic hotspots for further collection for the *ex situ* conservation of crop landrace groups. Geographic hotspots for further collection for the *ex situ* conservation of crop landrace groups. The map displays ‘gap richness’ across the predicted worldwide distributions of 71 landrace groups of 25 cereal, pulse, and starchy root/tuber/fruit crops within their geographic regions of diversity, indicating where landrace groups are expected to occur and have not yet been collected and conserved in genebanks. Darker colors indicate greater numbers of un-collected crop landrace groups potentially overlapping in the same 2.5 arc-minute cells. Source data

**Extended Data Fig. 6:**
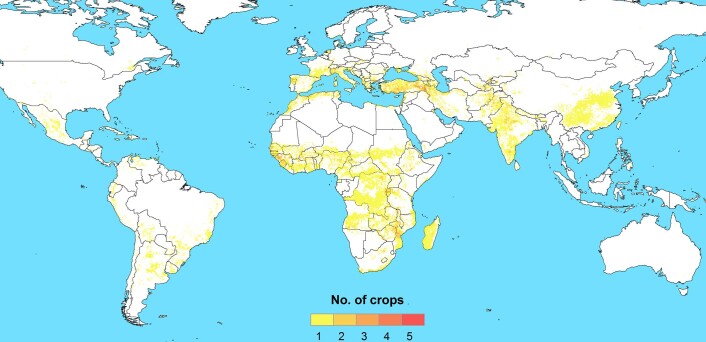
Geographic hotspots for further collection for the *ex situ* conservation of landrace groups of cereal crops. Geographic hotspots for further collection for the *ex situ* conservation of landrace groups of cereal crops. The map displays ‘gap richness’ across the predicted distributions of landrace groups of 9 cereal crops within their geographic regions of diversity, indicating where landrace groups are expected to occur and have not yet been collected and conserved in genebanks. Darker colors indicate greater numbers of un-collected cereal crop landrace groups potentially overlapping in the same 2.5 arc-minute cells, quantified in terms of numbers of crops. Source data

**Extended Data Fig. 7:**
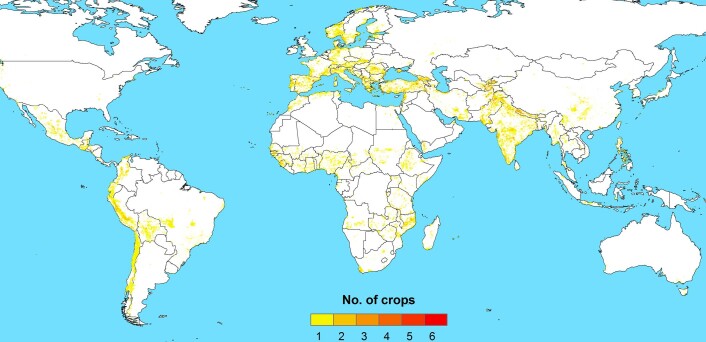
Geographic hotspots for further collection for the *ex situ* conservation of landrace groups of pulse crops. Geographic hotspots for further collection for the *ex situ* conservation of landrace groups of pulse crops. The map displays ‘gap richness’ across the predicted distributions of landrace groups of 9 pulse crops within their geographic regions of diversity, indicating where landrace groups are expected to occur and have not yet been collected and conserved in genebanks. Darker colors indicate greater numbers of un-collected pulse crop landrace groups potentially overlapping in the same 2.5 arc-minute cells, quantified in terms of numbers of crops. Source data

**Extended Data Fig. 8:**
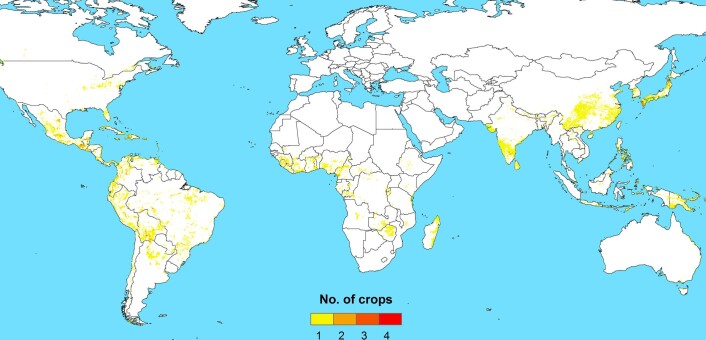
Geographic hotspots for further collection for the *ex situ* conservation of crop landrace groups of starchy root, tuber, and fruit crops. Geographic hotspots for further collection for the *ex situ* conservation of crop landrace groups of starchy root, tuber, and fruit crops. The map displays ‘gap richness’ across the predicted distributions of landrace groups of 7 starchy root, tuber, and fruit crops within their geographic regions of diversity, indicating where landrace groups are expected to occur and have not yet been collected and conserved in genebanks. Darker colors indicate greater numbers of un-collected starchy root, tuber, and fruit crop landrace groups potentially overlapping in the same 2.5 arc-minute cells, quantified in terms of numbers of crops. Source data

Across the landrace groups of all 25 crops, geographic areas identified as hotspots requiring further collecting for ex situ conservation were concentrated in South Asia; the Mediterranean and West Asia; Mesoamerica; West, East and Southern Africa; the Andean mountains; and Central and East Asia (Fig. 4 and Extended Data Fig. 5; online results at https://ciat.shinyapps.io/LGA_dashboard/). Currently, uncollected landrace groups of up to nine crops are potentially cultivated within single 2.5-arc-minute grid cells in India and Morocco and of up to eight crops in Algeria, Greece, Iran, Mexico, Pakistan, Sierra Leone and Turkey. Regarding types of assessed crops, cereal and pulse landrace group diversity was predicted to be particularly in need of further collecting in the Mediterranean and West Asia; South Asia; West, East and Southern Africa; Europe; the Andean mountains; and Mesoamerica. Conversely, starchy root/tuber/fruit crop landrace group ex situ conservation gaps were concentrated in East and Southeast Asia, South Asia, West Africa, South America and Mesoamerica (Extended Data Figs. 6–8).

## Discussion

**Extended Data Fig. 9:**
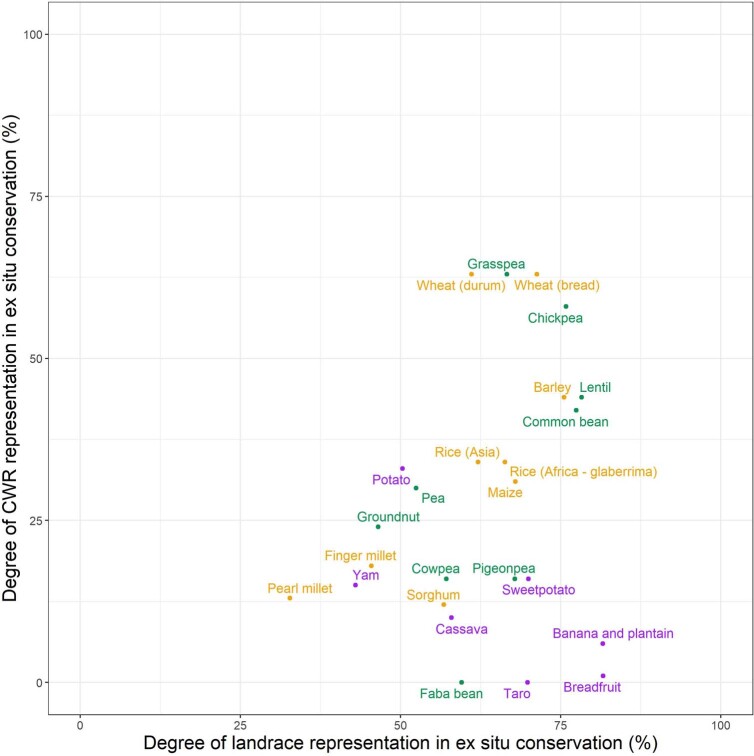
Comparison of *ex situ* conservation representation of crop landrace groups and crop wild relative (CWR) for 25 cereal, pulse, and starchy root/tuber/fruit crops. Comparison of *ex situ* conservation representation of crop landrace groups and crop wild relative (CWR) for 25 cereal, pulse, and starchy root/tuber/fruit crops. For CWR, conservation representation results were first averaged across CWR taxa in each crop genepool^19^. The summary results were also averaged across related crops assessed here; for example, the results for three yam crop genepools were averaged to form a single result for the global yam genepool. The crop genepool results were then transformed to the crop landrace scale and format used here, and are compared to the crop aggregated-level conservation representation average (%) estimate. Crop wild relatives of taro were not assessed in Castaneda-Alvarez et al. (2016)19; for this figure the pertinent score was set to zero. Cereals are displayed in gold, pulses in green, and starchy roots, tubers, and fruits in purple. Source data

Our analysis of the ex situ conservation status of landrace groups within 25 staple crops suggests that their representation in genebanks is most often substantial, a finding that highlights the impact of extensive international, national and subnational efforts worldwide over more than a half-century, both individually and via collaborative networks and initiatives^1,7,17,18^. Conservation of landraces of these crops—or at least their eco-geographically distinguishable groups—appears to be considerably further advanced than equivalent protection for crop wild relatives (Extended Data Fig. 9)^19,20^.

However, the findings also reveal that ex situ conservation gaps in terms of uncollected geographic and environmental variation across the distributions of landrace groups of these crops persist. Our quantitative and spatial results can aid in priority setting across these crops, their landrace groups and geographic regions, contributing to conservation targeting, planning and action. Further prioritization may be applied based on known or perceived threats related to economic, agricultural, technological, demographic, climatic and political change^1^. Recent decades of progress in clarifying and, in some cases, expediting the terms and conditions of genetic resources sampling and exchange^21,22^ bolster anticipation that such gaps can be filled through international collaboration. With further concerted efforts to collect crop landraces of these and other crops, a high degree of representation of their diversity in genebanks appears to be feasible, and, thus, the fulfilment of the international targets of the CBD^11^ and SDGs^12^ regarding their ex situ conservation also seems achievable. Conducted periodically over time, the gap analysis offers a more holistic approach to assess the state of landrace conservation than simply reporting changes in counts of accessions held in genebanks^23^ and, thus, may also represent a useful complement to the current indicators for these targets.

The landrace group classification and modelling processes described here demonstrate the potential to associate genetic, morphological, physiological, chemical, nomenclatural and other characteristics of cultivated plant populations with environmental and socioeconomic predictors within the regions of origin and diversity of crop taxa. These processes can be performed across a spectrum of infraspecific groups and geographic scales, depending on available knowledge and occurrence and characterization information. While our processes are based on openly available data and tools that undergo continual updating, they involve several limitations.

First, our methods are vulnerable to deficiencies in the quality, completeness and availability of occurrence and infraspecific grouping information. Many cultivated plants are insufficiently sampled, potentially due to a historical emphasis on wild rather than farming landscapes within biodiversity initiatives and persisting disconnects between biodiversity conservation and agricultural research communities^24^. Robust landrace classifications based on genetic structure, geography and other attributes also require further resolution for many crops.

The major biodiversity and conservation repository databases that we utilize here do not yet represent all pertinent national and subnational institutions worldwide; those institutions that do participate may not report all holdings and locality and characterization information is incomplete for many existing records^13,20^. Some additional information is probably present in other, smaller online databases or in offline or undigitized datasets. These gaps increase the uncertainty in our results, possibly leading to underestimations of the true degree of ex situ landrace conservation. On the other hand, the accessibility and long-term security of many such low-visibility collections are often equally uncertain^13,19^. Several processes would strengthen the conservation and potential usefulness of these genetic resources and the accuracy of analyses such as ours: the generation of characterization information and knowledge about infraspecific groups, improvements in the quality and completeness of existing occurrence information and better availability of landrace samples and their associated data, including safety duplication to better ensure long-term persistence.

Second, because our modelling method is based on statistical relationships between occurrences and environmental and socioeconomic predictor variables, it is also sensitive to the quality and comprehensiveness of these predictor datasets. Factors lacking predictor information or acting at finer scales than currently available data reflect will not be well incorporated into modelling processes. These may include environmental factors—both abiotic, such as soil characteristics or supplemental irrigation in small plots, and biotic, such as pathogen pressures or pollinator distributions—and socioeconomic drivers such as farm sizes, agronomic practices and seed system dynamics. Further, the models are unlikely to account for relatively recent disappearances of landraces unless such losses are associated with available predictors. The increasing generation of land-use-change information^25^ may partially resolve this challenge. In all cases, further development of high-resolution predictor datasets with global scope will improve modelling. Deeper understanding of the wide range of factors affecting farmer choices regarding landrace cultivation, including apparent stochasticity^26^, may also be important to improved modelling, while the limits to predicting distributions of populations whose ranges are driven by human preferences as much as environmental factors must be acknowledged.

Third, while geographic and ecological variation within predicted native ranges of plants has been shown to be an effective surrogate for direct measures of genetic diversity^27,28^, the modelling and conservation metrics used here may not fully reflect the distributions of and gaps in genetic variation within crop landraces. Further, our standardized method may not take into account the differences between crop species in genetic diversity within and among their populations, which may be influenced by reproductive biology, such as by outcrossing versus inbreeding species and by the mode of pollination; by mode of propagation, such as by seed versus clonally; and by other ecological and cultural factors^3^. Moreover, our conservation gap analysis methodology is based on the assumption that the existence of an ex situ accession from a site indicates that the targeted landrace group has been adequately sampled there. In reality, landrace distinctions at finer resolution than their modelled groupings may be ignored and, thus, not fully conserved. Previous field collecting may also not have comprehensively sampled populations at the resolution needed for all conservation, plant breeding or other research aims. This drawback may be particularly applicable to landraces that are typically genetically heterogeneous and, thus, may require large sample sizes to represent their diversity and, in particular, rare alleles. Finally, because in situ crop diversity constantly changes, developing novel variation from gene flow, recombination and mutation^6^, valuable new forms may have arisen in previously collected areas. Further sampling for ex situ conservation may, therefore, be warranted within or near previously collected sites.

The combination of these vulnerabilities reinforces the importance of field reconnaissance and of partnering with Indigenous and traditional agrarian communities and associated organizations to inform further collecting activities. In this sense, our results are best considered as support tools, useful for guiding rather than prescribing taxonomic and geographic priorities^13^. Additional essential steps include ensuring adherence to international, national and local sampling and exchange policies^21,22^; assessing field work risks, particularly in regions affected by war and civil strife^29^; and determining the most appropriate timing to maximize the harvest of viable seeds and other propagules. The capacity of pertinent genebanks to receive, adequately maintain and distribute landrace diversity must be preconfirmed^1,7^, and the logistics of getting collected material into relevant genebanks in a timely fashion must be established.

Further development and mobilization of landrace modelling and conservation gap analysis would ideally assess a wider range of crops, including fruits and vegetables, nuts and other groups of importance to human nutrition and agricultural livelihoods^30^. It is probable that many other crops, especially those that have not received primary focus in international or national genetic resources conservation and crop improvement efforts over the past half-century, are less well represented in ex situ conservation repositories^1,10^; thankfully, erosion of the in situ genetic diversity of these crops may be less severe thus far than in major staples^1^.

Geographic expansion of the analyses beyond historical regions of diversity^9,14,15^ may also aid in identifying novel variation, although further assessment of the correlation between landrace groups and spatial predictors in such regions will be necessary. To more fully address the scope of international conservation targets for landraces, these analyses must also assess the state of their in situ (on-farm) conservation; this task presents substantial challenges because emphasis in this context falls on the conditions and processes that foster landrace diversity rather than on the persistence of particular populations^1,2,31^. Given further development and expansion of the methods and scope, and the combination of the results with parallel analyses of crop wild relatives^19,20^ and other socioeconomically and culturally valuable plants^32^, a significantly improved understanding of distributions, protection status and conservation gaps across the major forms of crop diversity prioritized by the CBD and the SDGs should be achievable.

## Methods

### Crops and their landrace study areas

Food crops whose genetic resources are researched and conserved by CGIAR international agricultural research centres or by the CePaCT of the SPC were included in this study. Crop landrace distributions were modelled and conservation analyses conducted within recognized primary and, for some crops, secondary regions of diversity, where these crops were domesticated and/or have been cultivated for very long periods, and where they are, thus, expected to feature high genetic diversity and adaptation to local environmental and cultural factors (Supplementary Tables 1 and 2)^9,13^. These regions were identified through literature review (Supplementary Information) and confirmed by crop experts.

### Occurrence data

Our crop landrace group distribution modelling and conservation gap analysis rely on occurrence data, including coordinates of locations where landraces were previously collected for ex situ conservation and reference sightings. For ex situ conservation records, occurrences marked as landraces were retrieved from two major online databases: the Genesys Plant Genetic Resources portal^33^ and the World Information and Early Warning System on Plant Genetic Resources for Food and Agriculture (WIEWS) of the Food and Agriculture Organization of the United Nations^34^. Occurrences were also obtained directly from individual international genebank information systems: AfricaRice, the International Transit Centre and Musa Germplasm Information System of Bioversity International^35^, CePaCT, International Center for Tropical Agriculture (CIAT), International Maize and Wheat Improvement Center (CIMMYT), International Potato Center (CIP), International Center for Agricultural Research in the Dry Areas (ICARDA), International Crops Research Institute for the Semi-arid Tropics (ICRISAT), International Institute of Tropical Agriculture (IITA) and International Rice Research Institute (IRRI), as well as from the United States Department of Agriculture (USDA) Genetic Resources Information Network (GRIN)–Global^36^ and the Comisión Nacional para el Conocimiento y Uso de la Biodiversidad (CONABIO)^37^. Occurrences were compiled from the Global Biodiversity Information Facility (GBIF), with ‘living specimen’ records classified as ex situ conservation records and the remaining serving as reference sightings for use in distribution modelling. Reference occurrences were also drawn from published literature (Supplementary Information). Duplicated observations within or between data sources were eliminated, with a preference to utilize the most original data. Coordinates were corrected or removed when latitude and longitude were equal to zero or inverted, located in water bodies or in the wrong country or had poor resolution (<2 decimal places). Occurrences were clipped to study areas per crop. The complete occurrence dataset is available in Supplementary Dataset 2.

### Spatial predictors

We compiled and calculated spatially explicit gridded information for 50 potential environmental and cultural predictors of landrace distributions, including climatic, topographic, evolutionary history and socioeconomic variables (Supplementary Table 3)^13^. For climate data, we gathered or derived 39 variables from WorldClim version 2 (ref. ^38^) and Environmental Rasters for Ecological Modeling (ENVIREM)^39^. We included elevation from the Shuttle Radar Topography Mission (SRTM) dataset of the CGIAR–Consortium on Geospatial Information portal^40,41^. Two crop evolutionary history proxies were included: distance to human settlements before the year ad 1500 (ref. ^42^) and distance to known wild progenitor populations^13^. The eight socioeconomic variables included population density^43^, distance to navigable rivers^44^, percentage of the area under irrigation^45^, population accessibility^46,47^, geographic distributions of ethnic or cultural groups^48^ and crop harvested area, production quantity and yield^49^. All predictor data were scaled to 2.5-arc-minute resolution with World Geodetic System (WGS) 84 as a datum. Extended descriptions of the sources and their justification for inclusion are provided in Ramirez-Villegas et al.^13^. The complete spatial predictor dataset is available in Supplementary Dataset 3.

### Crop landrace group classifications

Crop landraces are cultivated plant populations managed by Indigenous or traditional farmers through cultivation, selection and diffusion^1^. They are typically genetically heterogeneous, although some types, such as clonally propagated populations, may be relatively homogeneous. They have recognizable characteristics, identities and geographic origins are in an ongoing process of adaptation to their local environments and societal conditions^1,2,31^. For most crops, landraces number in the thousands, with major global staple cereals such as rice and wheat potentially represented by hundreds of thousands of landraces^50,51^, although precise numbers and consensus regarding differentiations among landraces within crops have not been established. Given the diversity of landraces and the complexity of environmental and cultural drivers differentiating them, our method seeks a compromise between, on the one hand, acknowledgement of this diversity and, on the other, the feasibility and performance of distribution modelling and conservation gap analysis.

For each crop, we, therefore, conducted an extensive literature review to identify recognized infraspecific groups with distinct morphological, physiological, chemical, genetic, nomenclatural or other characteristics that could be tested for environmental and cultural associations (Supplementary Table 1 and Supplementary Information). The nature of these groups varied by crop and included genepools, races, genetic clusters and geographic or environmental groupings. Crops often had more than one proposed grouping or classification.

We then built and tested classification models to determine how well the proposed groups could be predicted and distinguished based on spatial predictors, drawing from the occurrence database and training datasets compiled from the literature review. A random forest^52^, a support vector machine^53^, the *K*-nearest neighbour (KNN) algorithm^54^ and artificial neural networks^55^ were used to determine classification performance. The response variable was the group to which a given occurrence was assigned, whereas the explanatory variables were the spatial predictors. Models were combined into an ensemble using the mode—that is, the most frequent predicted value among the models—and tested using 15-fold cross-validation with 80% training and 20% testing. We accepted a given classification if each of its components was predicted with an average cross-validated accuracy of at least 80%. In the case of multiple classification proposals per crop, we selected the one with the best overall performance. Finally, we used the trained models to predict the corresponding group for occurrences missing such information. All landrace groups for all crops are provided in Supplementary Table 2, with the best-performing groups identified.

### Crop landrace group distribution modelling

To predict the probability of geographic occurrence for each landrace group within each crop, we generated MaxEnt models^56,57^ using the ‘maxnet’ R package^58^. Group-specific spatial predictors were selected using a combination of the variance inflation factor (VIF) and a principal component analysis (PCA) to control for excessive model complexity and variable collinearity^59^. We removed variables that did not contribute significantly to the variance in the PCA, defined as contributing less than 15% to the first component, and we further discarded variables with a VIF > 10 (ref. ^60^). The predictors and whether they were selected for the modelling of each landrace group are presented in Supplementary Table 4.

We generated a random sample of pseudo-absences as background points in areas that (1) were within the same ecological land units^61^ as the occurrence points, (2) were deemed potentially suitable according to a support vector machine classifier that uses all occurrences and predictor variables and (3) were farther than 5 km from any occurrence^62^. The number of pseudo-absences generated per crop group was ten times its number of unique occurrences.

MaxEnt models were fitted through five-fold (*K* = 5) cross-validation with 80% training and 20% testing. For each fold, we calculated the area under the receiving operating characteristic curve (AUC), sensitivity, specificity and Cohen’s kappa as measures of model performance. To create a single prediction that represents the probability of occurrence for the landrace group, we computed the median across *K* models. Geographic areas in the form of pixels with probability values above the maximum sum of sensitivity and specificity were treated as the final area of predicted presence^13^.

### Ex situ conservation status and gaps

Three separate but complementary metrics were developed to compare the geographic and environmental diversity in current ex situ conservation collections to the total geographic and environmental variation across the crop landrace group distribution model and, thus, to identify and quantify ex situ conservation gaps^13^.

A connectivity gap score (*S*_CON_) was calculated for each 2.5-arc-minute pixel within the distribution model by drawing a triangle^63,64^ around each pixel using the three closest genebank accession occurrence locations as vertices and then deriving normalized values for the pixel based on distance to the triangle centroid and vertices^13^. The *S*_CON_ of a pixel is high—closer to 1 on a scale of 0–1—when its corresponding triangle is large, when the pixel is close to the centroid of the triangle or when the distance to the vertices is large. A high *S*_CON_ represents a greater probability of the pixel location being a gap in existing ex situ collections.

An accessibility gap score (*S*_ACC_) was calculated for each 2.5-arc-minute pixel in the distribution model by computing travel time from each pixel to its nearest genebank accession occurrence location based both on distance and the speed of travel, defined by a friction surface^13,45^. Travel time scores were normalized by dividing pixel values by the longest travel time within the distribution model, with the final score ranging from 0 to 1. A high *S*_ACC_ value for a pixel reflects long travel times from existing genebank collection occurrences and, thus, represents a higher probability of the pixel location being a gap in existing ex situ collections.

An environmental gap score (*S*_ENV_) was calculated for each 2.5-arc-minute pixel in the distribution model by conducting a hierarchical clustering analysis using Ward’s method with all the predictor variables from the distribution modelling. The Mahalanobis distance between each pixel and the environmentally closest genebank accession occurrence location was then computed^13^. Environmental distance scores were normalized between 0 and 1. A high *S*_ENV_ value for a pixel reflects a large distance to areas with similar environments where landraces have previously been collected for genebank conservation and, thus, represents a higher probability of the pixel location being a gap in existing ex situ collections.

Spatial ex situ conservation gaps were determined from the conservation gap scores using a cross-validation procedure to derive a threshold for each score. We created synthetic gaps by removing existing genebank occurrences in five randomly chosen circular areas with a 100 km radius within the distribution model. We then tested whether these artificial gaps could be predicted by our gap analysis, identifying the threshold value of each score that would maximize the prediction of these synthetic gaps. Performance for each of the five gap areas was assessed using AUC, sensitivity and specificity. The average cross-area threshold value was calculated for each score to discern pixels with a high likelihood of finding ex situ conservation gaps and that, thus, were higher priority for further field sampling. These were pixels with combined gap scores above the threshold, assigned a value of 1, as opposed to the relatively well-conserved areas below the threshold, which were assigned a value of 0.

The three binary conservation gap scores were then mapped in combination, resulting in pixels across the distribution model with gap values ranging from 0 to 3. Pixels with a value of 0 display no connectivity, accessibility or environmental gaps and are considered well represented ex situ. Pixels with a value of 1 indicate a conservation gap in connectivity, accessibility or the environment; we consider these ‘low-confidence’ gaps. Pixels with a value of 2 indicate gaps in two metrics or ‘medium-confidence’ gaps, and values of 3 indicate gaps across all metrics or ‘high-confidence’ gaps. High-confidence gap areas are displayed on crop-conservation-gap maps (Fig. 2b and Supplementary Information) and conservation hotspot maps across crops (Fig. 4 and Extended Data Figs. 5–8).

The representation of crop landrace groups in current ex situ conservation collections was calculated based on the final 1–3 value conservation-gap maps. The complement of the proportion of the modelled distribution considered as a potential conservation gap by any single gap score represents the minimum estimate of current representation; the complement of the proportion considered by all three scores as a gap, which is to say high-confidence gap areas, represents the maximum estimate (Supplementary Tables 1 and 2).

While distribution modelling and conservation gap analyses were conducted at the crop landrace group level and results are presented in full in the Supplementary Information, for ease of comparison of results across crops, and to avoid bias towards crops with many landrace groups, we also calculated summary results at the crop level. Crops that had been assessed with geographic differentiations, including maize in Africa and Latin America and yams in the New World and the Old World, were also combined. For spatial results, the pixels in crop landrace group models were summed—that is, constituent landrace group models were combined. The minimum and maximum current conservation representation estimations at the crop level were then calculated based on combined spatial models.

### GBIF occurrence downloads

The following occurrence downloads from the Global Biodiversity Information Facility (GBIF; https://www.gbif.org/, 2017−2021) were used: 10.15468/dl.rrntfr, 10.15468/dl.2f2v4h, 10.15468/dl.2ywlb7, 10.15468/dl.lnfelh, 10.15468/dl.ryrmfj, 10.15468/dl.8adf61, 10.15468/dl.nff5ys, 10.15468/dl.erxs6e, 10.15468/dl.vbfgho, 10.15468/dl.mjjk3x, 10.15468/dl.uppz1n, 10.15468/dl.938bgm, 10.15468/dl.hr87hm, 10.15468/dl.k1va80, 10.15468/dl.coqpu2, 10.15468/dl.lkoo9u, 10.15468/dl.e998mp, 10.15468/dl.vfbmm7, 10.15468/dl.tnp478, 10.15468/dl.6zxsea, 10.15468/dl.0lray8, 10.15468/dl.5sjgsw, 10.15468/dl.wkju6h, 10.15468/dl.7xzfvc, 10.15468/dl.autlf5, 10.15468/dl.fe2amw, 10.15468/dl.2zblvz, 10.15468/dl.ddplkj, 10.15468/dl.jbzejg, 10.15468/dl.ej5bha, 10.15468/dl.905pxd, 10.15468/dl.pim1vs, 10.15468/dl.vdridc, 10.15468/dl.b43gyv, 10.15468/dl.nnw3z7, 10.15468/dl.bnt9jc, 10.15468/dl.f5x2cg, 10.15468/dl.ub7zbg, 10.15468/dl.sggf2v, 10.15468/dl.ath5ve, 10.15468/dl.23k3ug, 10.15468/dl.cym376, 10.15468/dl.53bwzk, 10.15468/dl.fsad7h and 10.15468/dl.fm6p7z.

### Reporting Summary

Further information on research design is available in the Nature Research Reporting Summary linked to this article.

## Supplementary information

Supplementary Information Supplementary Methods and References.

Reporting Summary

Supplementary Tables Supplementary Table 1: Crops and their study areas, assessed landrace groups and ex situ conservation coverage results. Supplementary Table 2: All crop landrace groups and their study areas, classification performance, modelling metrics and ex situ conservation coverage results. Supplementary Table 3: List of spatial predictors and their sources. Supplementary Table 4: Spatial predictors and whether they were employed for classification and modelling for each crop landrace group.

## Data Availability

Occurrence data, including spatial predictor variable results (at 2.5-arc-minute resolution) for each occurrence (Supplementary Dataset 2) and the global spatial predictor dataset (2.5-arc-minute resolution, all 50 variables) (Supplementary Dataset 3) are available at 10.7910/DVN/J8WAPH. Source data are provided with this paper.

## Source data

Source Data Fig. 1 Raster.
Source Data Fig. 2 Raster.
Source Data Fig. 3 Excel data.
Source Data Fig. 4 Raster.
Source Data Extended Data Fig. 1 Raster.
Source Data Extended Data Fig. 2 Raster.
Source Data Extended Data Fig. 3 Raster.
Source Data Extended Data Fig. 4 Raster.
Source Data Extended Data Fig. 5 Raster.
Source Data Extended Data Fig. 6 Raster.
Source Data Extended Data Fig. 7 Raster.
Source Data Extended Data Fig. 8 Raster.
Source Data Extended Data Fig. 9 Excel data.

## Code availability

Code for the crop landrace group classification testing, distribution modelling and conservation gap analysis is available at https://github.com/CIAT-DAPA/gap_analysis_landraces.
