# Supplementary material for: State of ex situ conservation of landrace groups of 25 major crops
Source: Nat Plants. 2022 May 9;8(5):491–9. doi: 10.1038/s41477-022-01144-8 (PMC9122826; doi:10.1038/s41477-022-01144-8)
Supplement: Supplementary file 1 — Supplementary Methods and References. [file 41477_2022_1144_MOESM1_ESM.pdf]

nature portfolio

https://doi.org/10.1038/s41477-022-01144-8

# Supplementary information

# State of ex situ conservation of landrace groups of 25 major crops

In the format provided by the authors and unedited

# Supplementary Information

## Supplementary Methods and References

### *Crop landrace groups*

For each crop, we conducted a literature review to identify recognized infraspecific groups with distinct genetic, morphological, physiological, chemical, nomenclatural, or other characteristics that could be tested for environmental and cultural associations. These groups varied by crop and included genepools, races, genetic clusters, and geographic or environmental groupings. Crops often had more than one proposed grouping or classification. For each crop, the landrace groups we explored are described below.

*Cereals*

*Barley*

Cultivated barley (*Hordeum vulgare* L. or *Hordeum vulgare* L. subsp. *vulgare*) was domesticated from the wild progenitor *Hordeum vulgare* L. subsp. *spontaneum* (K. Koch) Thell., a species with a broad native distribution including Egypt, Libya, Armenia, Azerbaijan, China, Kyrgyzstan, Turkmenistan, Uzbekistan, Afghanistan, Cyprus, Iran, Iraq, Israel, Jordan, Lebanon, Syria, Turkey, India, Pakistan, and Greece (USDA ARS NPGS GRIN Global taxonomy 2021). Two-rowed types of barley, which have two rows of kernels with shattering spikes--that is, wild barleys--have been classified as *H. vulgare* subsp. *spontaneum* = *Hordeum spontaneum* K. Koch.), whereas two-rowed barleys with nonshattering spikes--that is, domesticated barleys--have been classified as *H. vulgare* subsp. *vulgare* (= *Hordeum distichum* L.). Six-rowed barleys with nonshattering spikes are also classified as *H. vulgare* subsp. *vulgare* ( = *Hordeum hexastichum* L.). There are also known populations of six-rowed barley with shattering spikes, which have also been classified as *H. vulgare* subsp. *vulgare* (= *Hordrum agriocrithon* Åberg.), because this is considered a weedy/feral taxon caused by introgressions between domesticated and wild types. Drawing on further cytological and molecular evidence, most recent classifications treat all these forms as a single species, *H. vulgare* L. [1], because head type differences are known to be driven by single-gene mutations [2,3].

Barley was domesticated over 10,000 years ago in the Fertile Crescent. By 4200 BCE it was known in Eastern Finland, and from 1500–850 BCE on the Korean Peninsula [4]. Tibet has been posited as a secondary center of diversity and of domestication [5] but is unlikely to have been a primary area of domestication [6]. Wild barley may have three distinct distributions that may coincide with the three centers of origin of the crop: the Fertile Crescent, Central Asia, and China in the Tibetan Plateau [7]. Orabi et al. (2007) [8] posited independent domestication in eastern

Africa, while Morocco has also been identified as a potential area of domestication. While distinct genetic variation both in the crop and its wild relatives occurs in all these regions except Ethiopia and Morocco, where wild barley is not known, the leading hypothesis remains a monophyletic origin and domestication in the Fertile Crescent [9].

Major distinguishing characters differentiating barley landrace types include whether the type has two or six kernel rows, as described above [1,2] ; whether the kernel is covered or hulled, on one hand, or naked or hulless, on the other; and whether the type is sown in spring or winter [10]. Other, often related, characteristics may include daylength responsiveness; protein, especially hordein, type or content; compact versus lax spike form; long versus trifurcate awn length and branching;  developed versus reduced lateral spikelet form; long versus shortened spike length; seed protein (aleurone) color; and various genetic markers [2,11,12]. Based on available information and occurrence data, we tested the three main character groups (row number, kernel covering, and spring/winter).

The main testing response data included the ICARDA genebank database and Russell et al. (2016) [3]; for the latter we obtained data directly from one of the authors, B. Kilian. The ICARDA dataset contained passport data about occurrence and locality as well as information about barley characteristics including growth class, kernel row number, and kernel covering. The Russell et al. (2016) [3] dataset included passport and genetic structure data. The singular kernel covering type classifier provided the greatest accuracy (average accuracy of 98%; 100% of the occurrences well classified in *covered grains*) and was thus used for the combined landrace gap analysis (Supplementary Dataset 1 [Supplementary Table 2]).

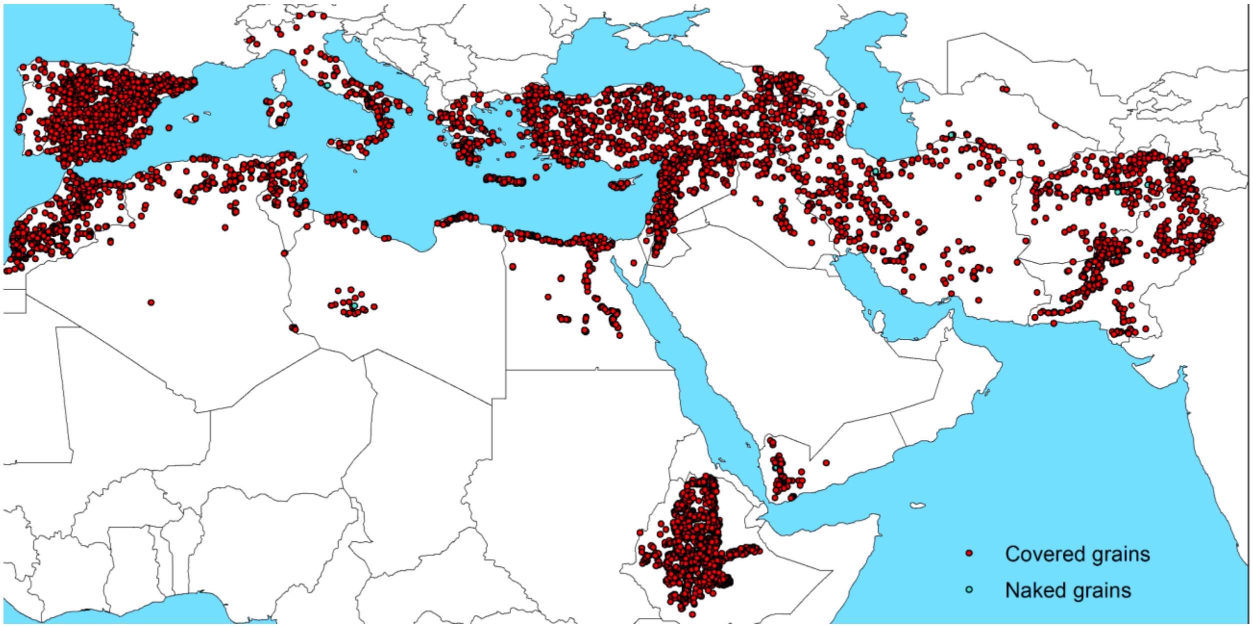


**Supplementary Fig. 1a:** All occurrences within the study region of barley landrace groups.

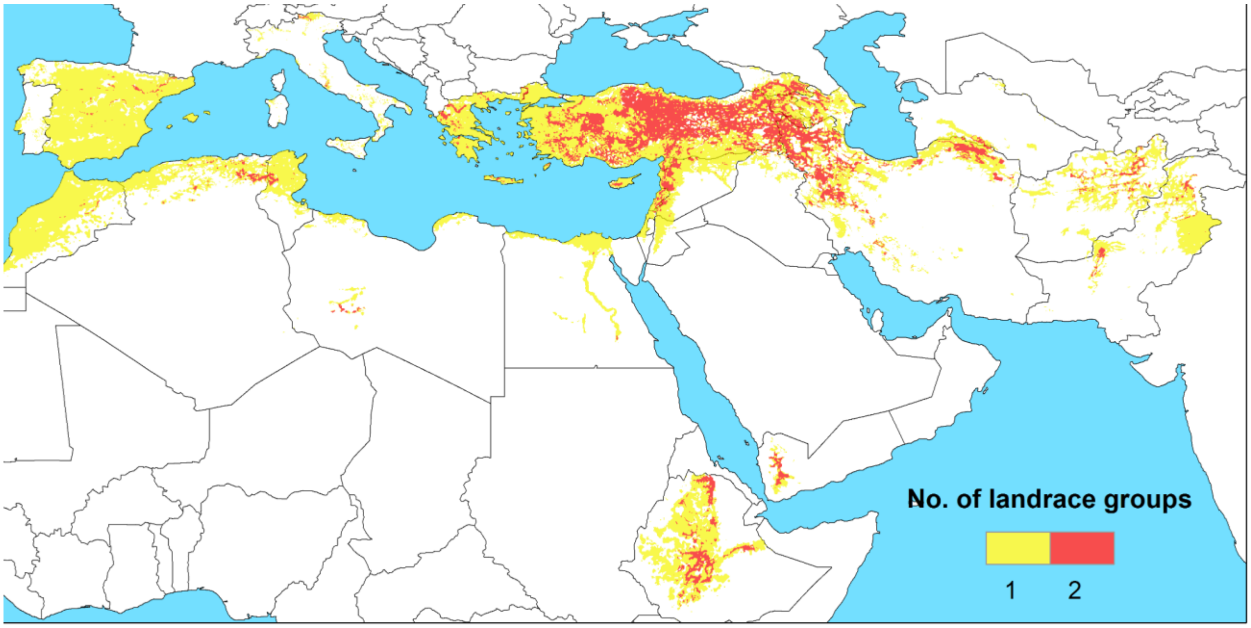


**Supplementary Fig. 1b:** Predicted distributions of barley landrace groups.

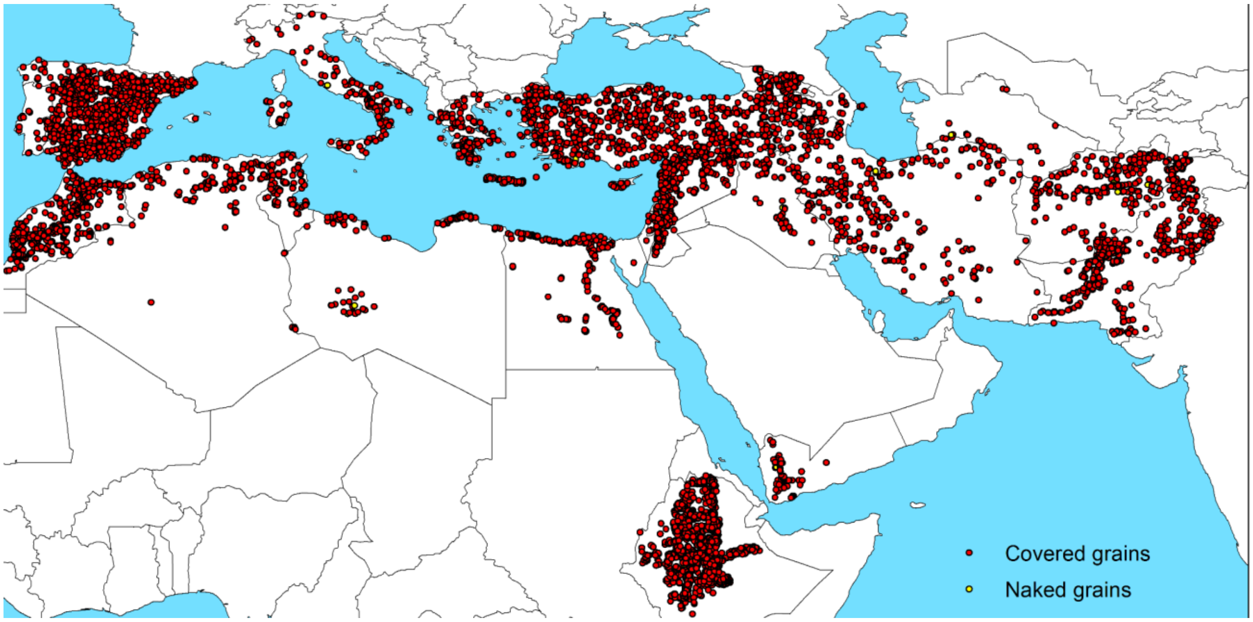

**Supplementary Fig. 1c:** Existing *ex situ* collection occurrences of barley landrace groups.

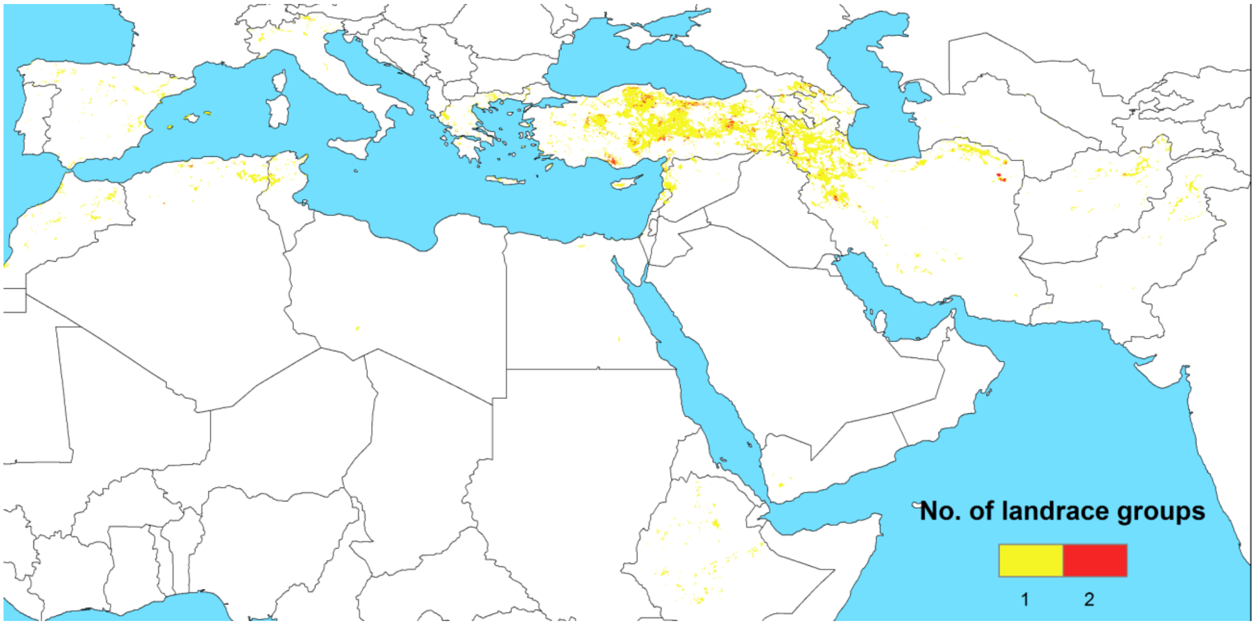

**Supplementary Fig. 1d:** Geographic gaps in the *ex situ* conservation of barley landrace groups.

*Finger millet*

Our literature review indicated that finger millet (*Eleusine coracana* [L]) Gaertn.) is cultivated in eastern and southern Africa and across most of southern Asia, usually for food and making beer [13]. The *Eleusine* genus is predominantly African, with seven of its nine species occurring on the continent [14]. *E. coracana* is generally proposed to be derived from *E. indica* (L.) Gaertn. It was likely domesticated around 5,000 years BP in eastern Africa and introduced to India 3,000 years ago. It is possible to contrast infraspecific (racial) evolution of the crop between African and Indian types.

*E. coracana* (L.) Gaertn. subsp. *coracana* includes all the cultivated finger millets, and most of their characteristics are similar. Their inflorescence shape, however, is widely variable and correlates to geographical distribution, which allowed Hilu and de Wet (1976) [13] to recognize three races of cultivated finger millet: the African highland race, the African lowland race, and the Indian race. On the contrary, Hussaini, et al. (1977) [15] identified twelve groups based on a principal component analysis of inflorescence and vegetative traits. De Wet et al. (1984) [16] performed a comparative morphological study of the ICRISAT collection, coupled with a discriminant function analysis of quantitative characteristics, and identified four cultivated races with eleven distinct cultivated complexes: race *coracana,* race *elongata, plana,* and race *vulgaris.* The authors argue that racial evolution started in Africa before the crop was introduced to India.

Based on available data, the structure suggested by De Wet et al. (1984) [16] was used to generate classification models from the ICRISAT data; predict unclassified occurrences in the data from Genesys, GBIF, and the USDA; and develop the gap analysis for cultivated finger millet across its geographical distribution area. The machine learning models reached an average accuracy of 64%, classifying 94% of the occurrences belonging to race *vulgaris*, 21.42% of the occurrences belonging to race *elongata,* 16.2% of the occurrences belonging to race *plana*, but none of the occurrences belonging to race *compacta.*

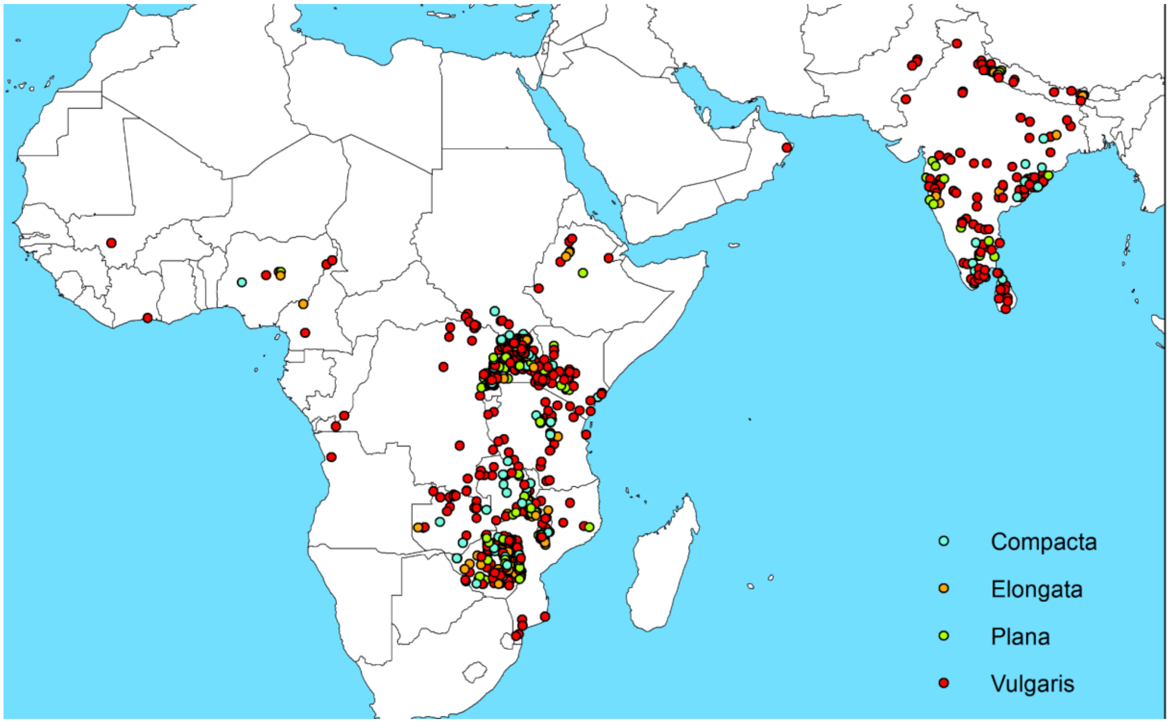


**Supplementary Fig. 2a:** All occurrences within the study region of finger millet landrace groups.

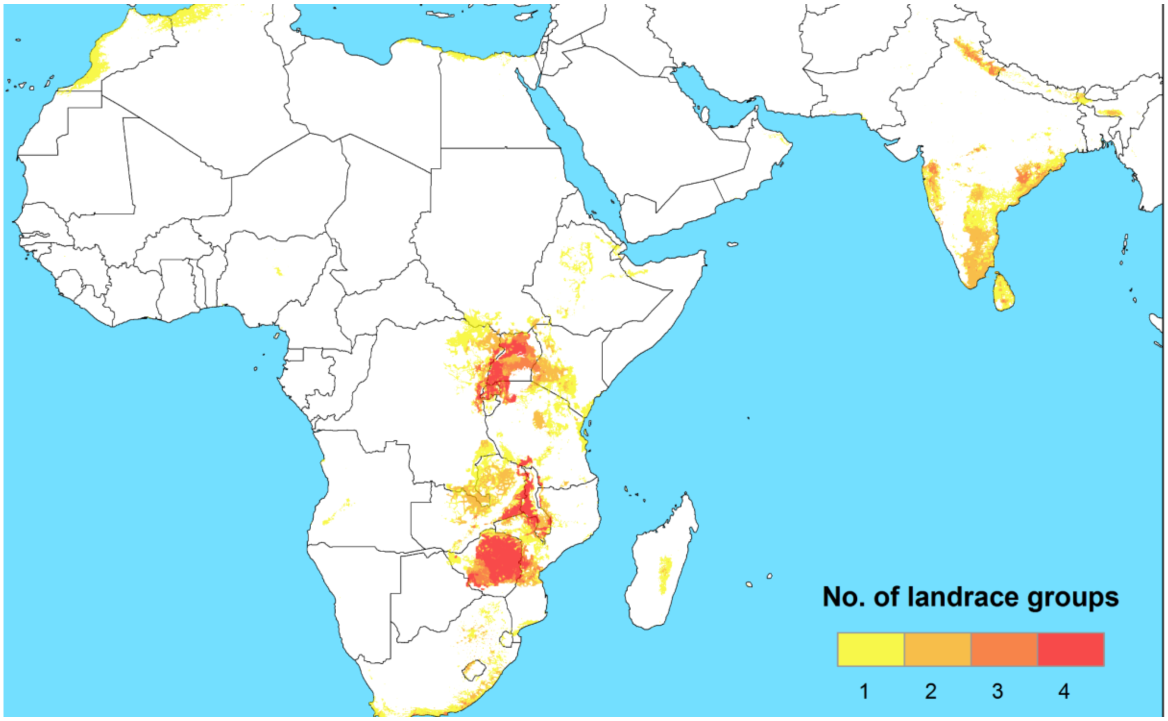


**Supplementary Fig. 2b:** Predicted distributions of finger millet landrace groups.

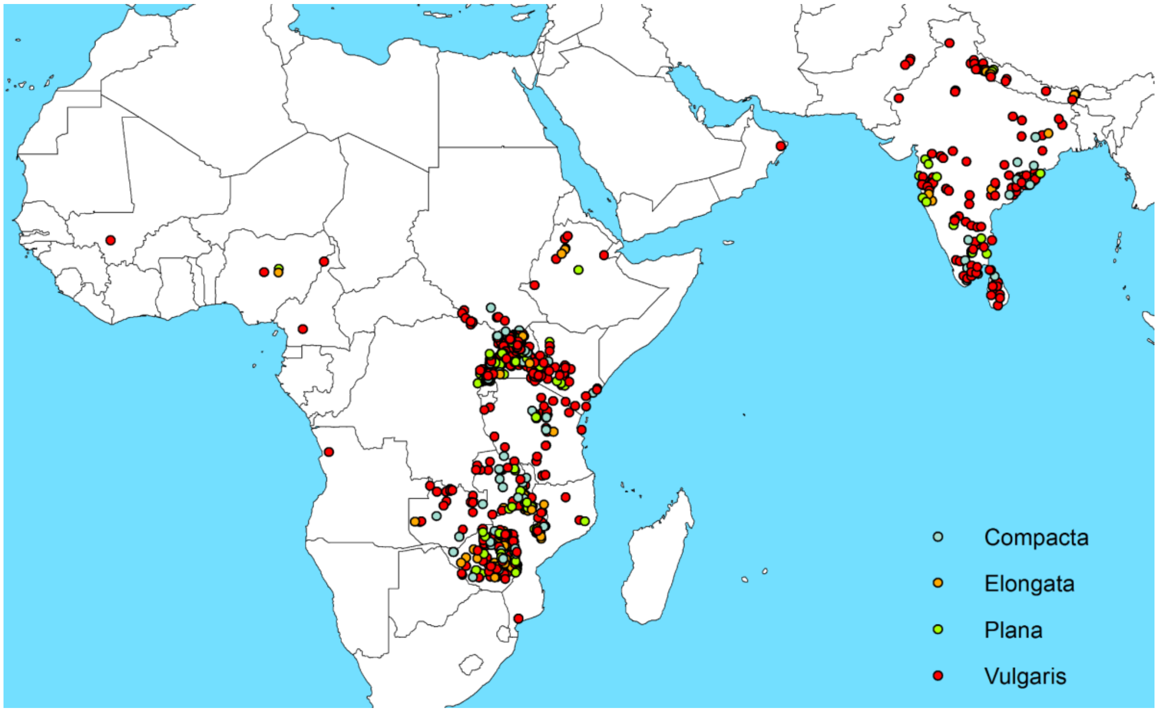


**Supplementary Fig. 2c:** Existing *ex situ* collection occurrences of finger millet landrace groups.

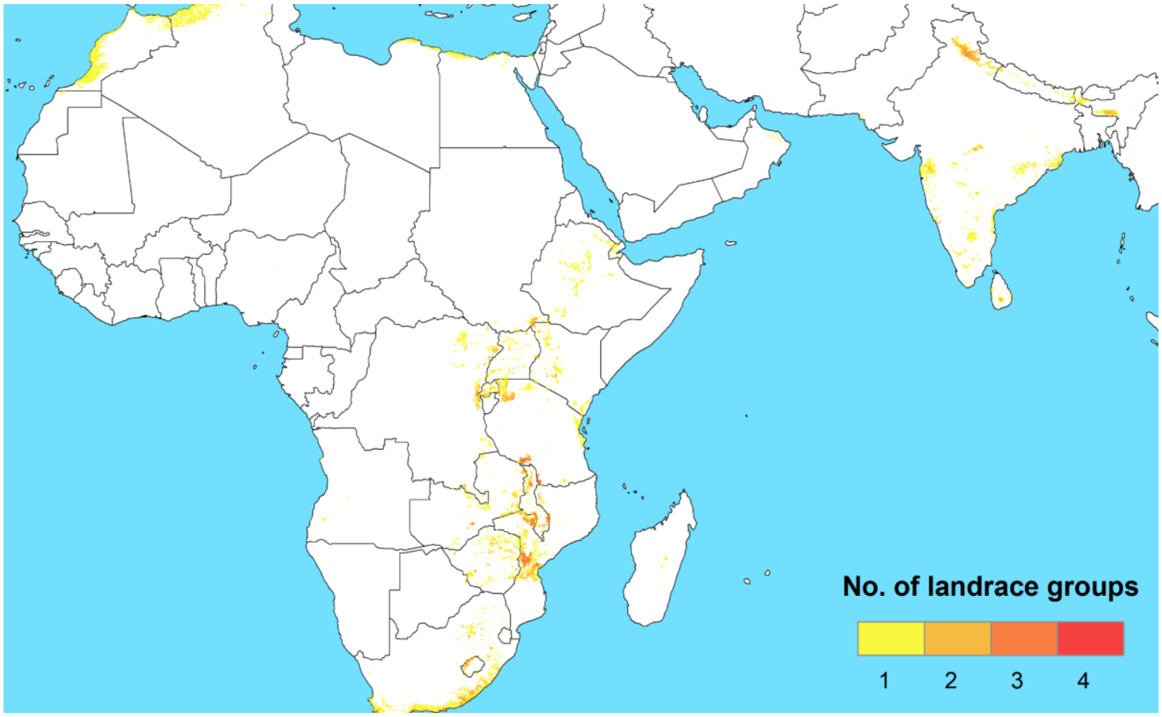


**Supplementary Fig. 2d:** Geographic gaps in the *ex situ* conservation of finger millet landrace groups.

*Maize*

Our literature review indicated that maize (*Zea mays* L. subsp. *mays*) was domesticated about 9,000 years ago in the Balsas River region of western Mexico from its wild progenitor teosinte (*Zea mays* L. subsp. *parviglumis* H. H. Iltis & Doebley) [17-19]. The oldest surviving maize types are from the Mexican highlands, where maize diversified before moving into the lowlands. Maize then spread through migration and trade routes across the Americas along two major paths. One path proceeds through western and northern Mexico into the southwestern U.S. and then into the eastern U.S. and Canada. A second path leads out of the highlands through the western and southern lowlands of Mexico into Guatemala, the Caribbean Islands, the lowlands of South America, and finally the Andes Mountains. This dispersal process has produced a wide variety of phenotypic traits associated with local environmental and cultural conditions [19].

Diverse studies have tested different groupings of maize landrace structures in the Americas using genetic data and defining the groups typically by their geographic location, instead of using a taxonomic hierarchy [17-20]. Based on published passport data--accession identifiers, geographic coordinates, and the tested groups--from these four studies, the response of these grouping structures was proven using climate and socioeconomic variables in a classification analysis. The response variable corresponds to the groups published in each paper, and the predictor variables correspond to the list of predictors previously described (Supplementary Dataset 1 [Supplementary Table 3]).

Based on the classification analysis, the genetic structure best represented by climate and socioeconomic conditions corresponds to the groups from van Heerwaarden et al. (2011) [18], with adaptations:

- The Mesoamerican lowlands
- The Mexican highlands
- The South American lowlands
- Other groups in South America
- The U.S.
- Western Mexico
- The Andean highlands

The adapted landrace structure and the trained classification model from van Heerwaarden et al. (2011) [18] were used to predict the unclassified accessions and develop the landrace gap analysis for maize in the Americas. The machine learning models reached an average accuracy of 74.1% , classifying the 86.7% of the occurrences belonging to the Andean highlands; 53.3% of the occurrences belonging to the Mexican highlands; 71.4% of the occurrences belonging to the Mesoamerican lowlands; 68.9% of the occurrences belonging to the South American lowlands;

90% of the occurrences belonging to other groups in South America; 80% of the occurrences belonging to the U.S; and 68.42% of the occurrences belonging to the Western Mexico.

Few studies (but see Mir et al. [2013] [20]) investigate infraspecific structure of maize in Africa by taxonomy or genomics. Therefore, a cluster analysis using environmental variables was carried out over all the accessions. Using environmental and socioeconomic data, a PCA plus a hierarchical clustering on principal components was developed to obtain the optimal number of groups. As a result, four environmental clusters were identified. The biplot figure displays the grouping of the accessions and the influence upon them of the environmental and socioeconomic variables.

The environmental clusters in Africa may be described as follows:

- Group 1: Dry with high temperatures
- Group 2: High-altitude
- Group 3: Ample precipitation during the coldest trimester
- Group 4: Ample precipitation during the driest month

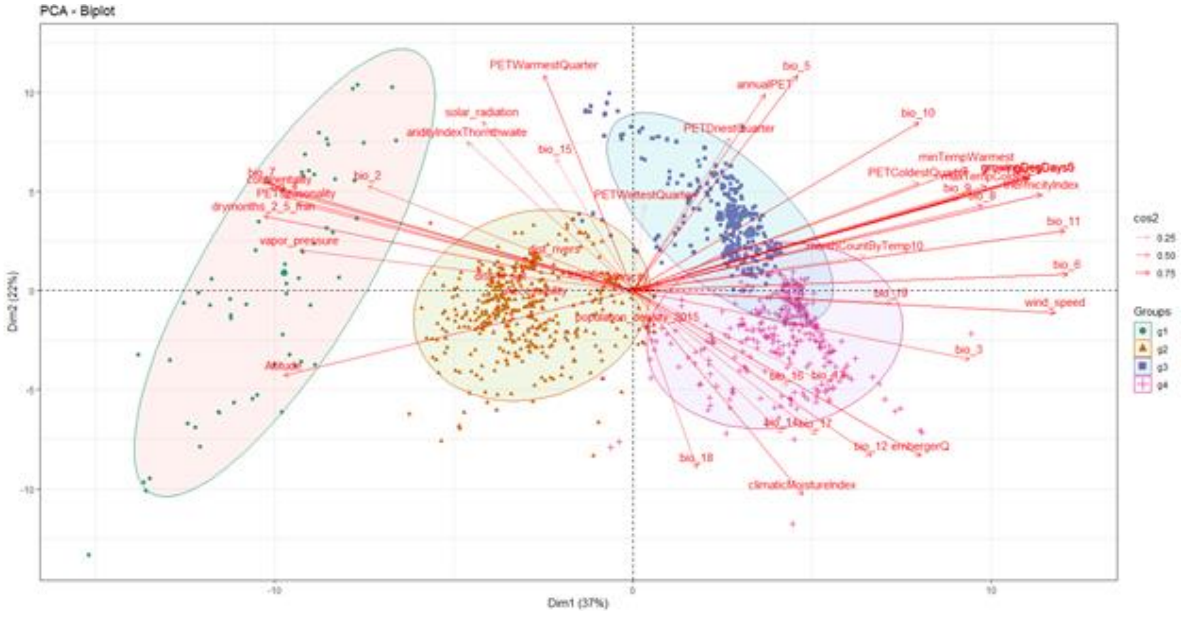


The machine learning models performed with the four groups, reached an average accuracy of 97.9%, classifying all the occurrences belonging to the Group 1; 99.2% of the occurrences belonging to the Group 2; 96,9% of the occurrences belonging to the Group 3; and 96.9% of the occurrences belonging to the Group 4.

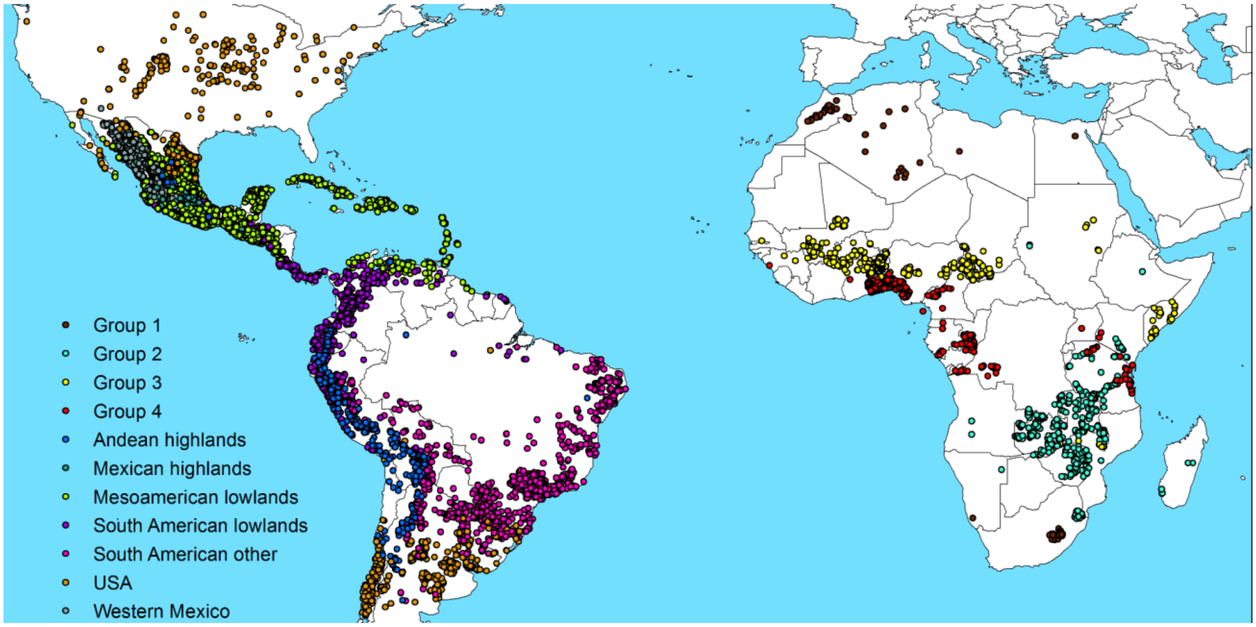


**Supplementary Fig. 3a:** All occurrences within the study region of maize landrace groups.

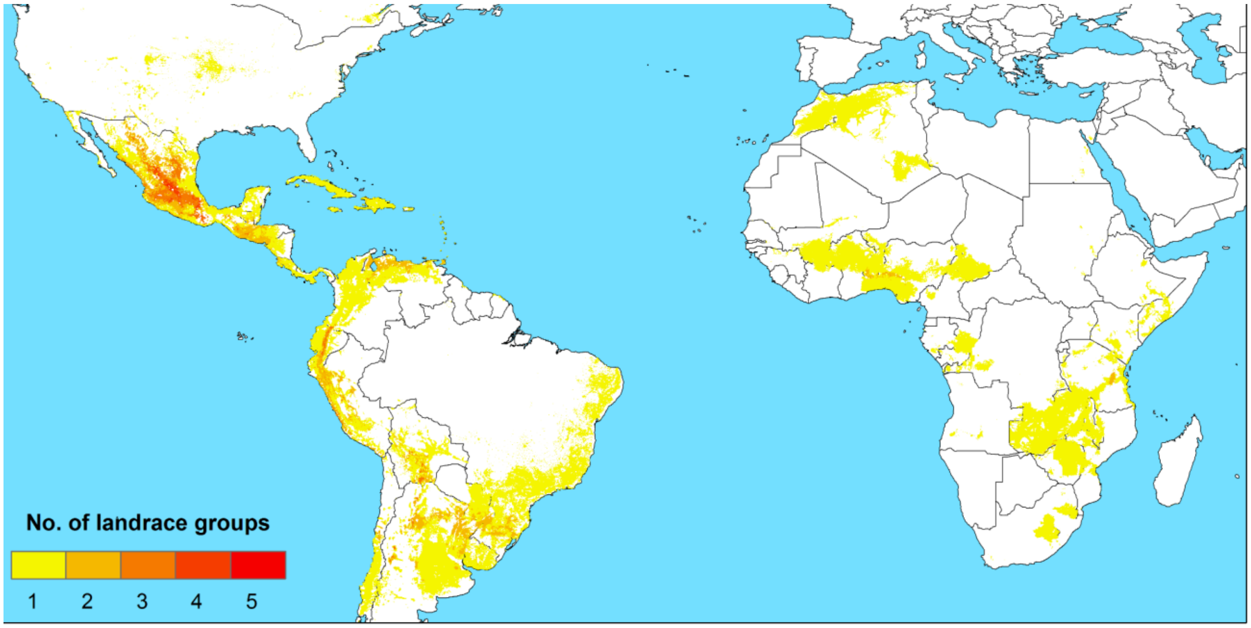


**Supplementary Fig. 3b:** Predicted distributions of maize landrace groups.

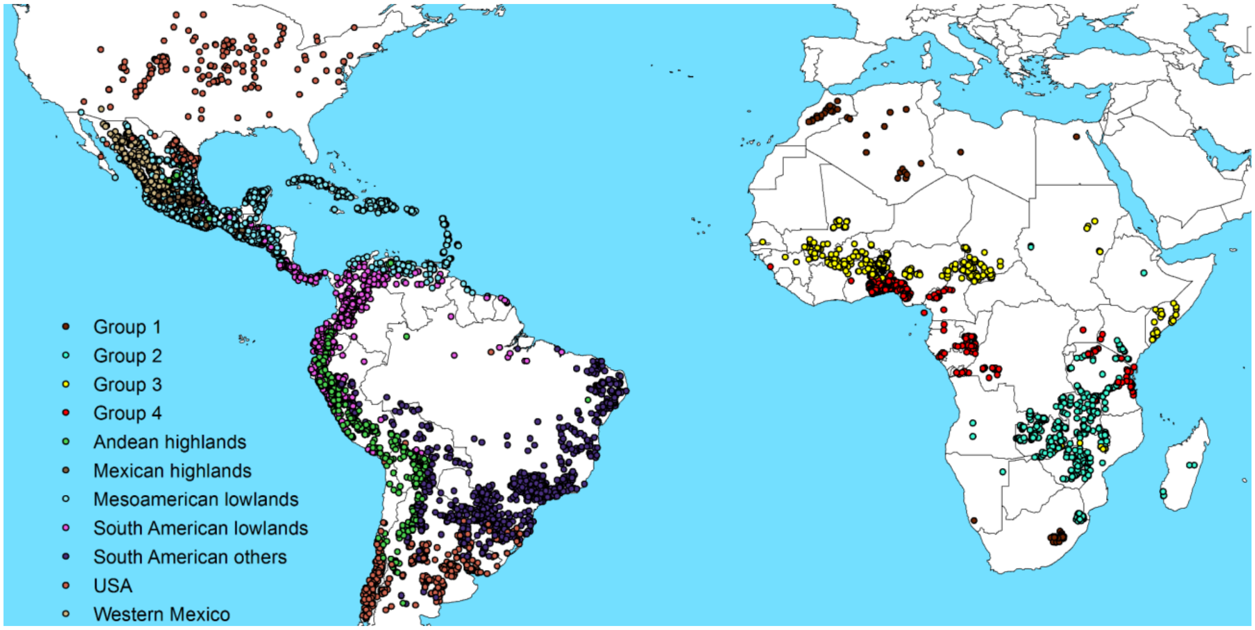


**Supplementary Fig. 3c:** Existing *ex situ* collection occurrences of maize landrace groups.

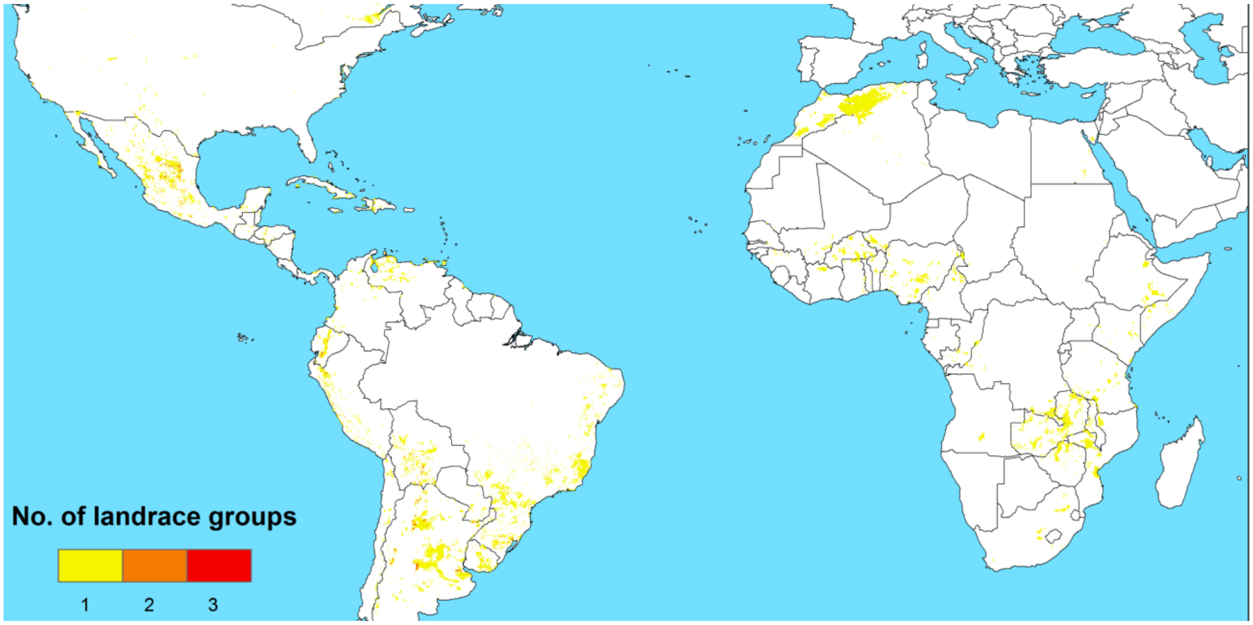


**Supplementary Fig. 3d:** Geographic gaps in the *ex situ* conservation of maize landrace groups.

*Pearl millet*

Pearl millet (*Cenchrus americanus* (L.) Morrone, formerly *Pennisetum glaucum* (L.) R. Br.) is a staple food for people from the semiarid regions of Africa and Asia with a very high photosynthetic efficiency, short growing period (duration), and a high degree of tolerance to heat and drought [21]. Domestication appears to have occurred independently in several regions from Mauritania to western Sudan [22-26].

The ICRISAT genebank has characterized and evaluated all of its cultivated accessions for 23 morpho-agronomic characteristics following the descriptors for pearl millet [27]. Characterization data from ICRISAT was used to perform a cluster analysis in order to understand how occurrences can be grouped, with particular attention to inflorescence and seed characteristics [28-30]. The variables that contributed the most to define discernable clusters were as follows included panicle length (cm) after the rainy season, panicle length (cm) during the rainy season, panicle exertion (cm), plant height (cm) during the rainy season, and plant height (cm) after the rainy season. Five main clusters were identified.

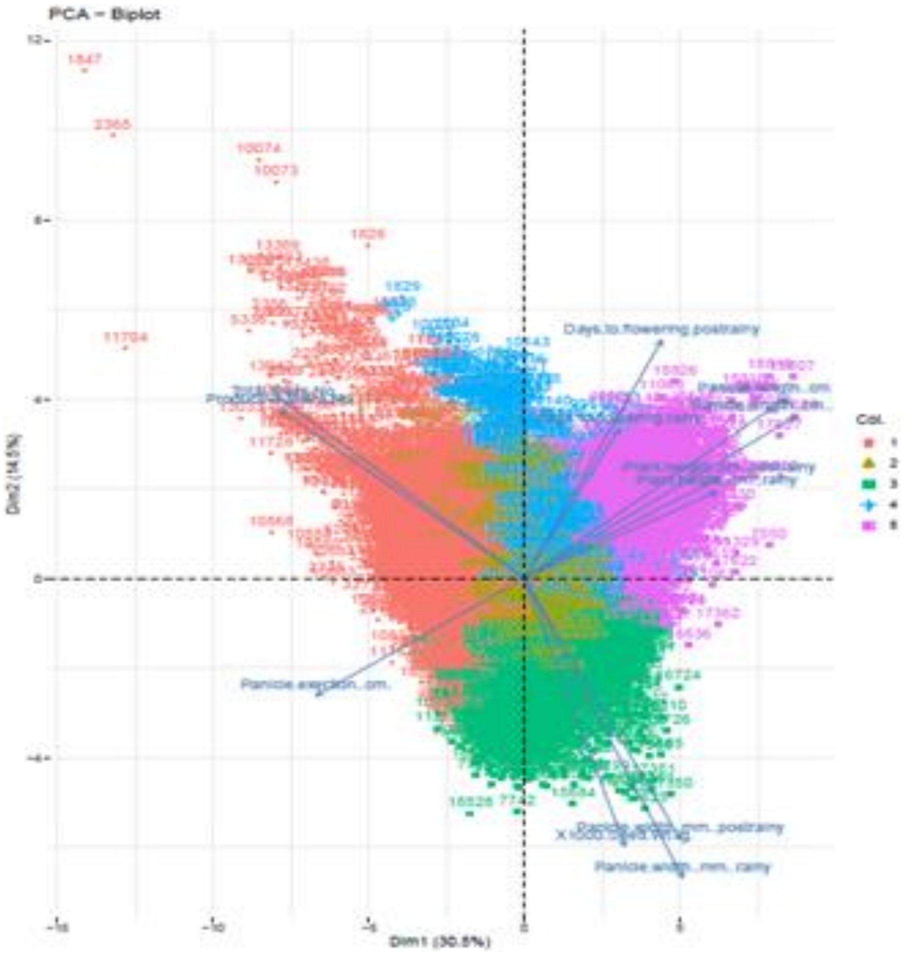

As the biplot shows, occurrences belonging to group 1 are described mostly by the larger number of tillers and productive tillers; also, their panicle is narrower even after the rainy season. Their seeds are the smallest, and their plants are relatively short. Finally, their panicle exertion--the length of the peduncle emerged from the flag leaf sheath--is larger. Occurrences belonging to group 2, meanwhile, are characterized by medium seed size, but most take more days to flower in the rainy season, and their plants are the largest during the rainy season. Occurrences belonging to group 3, on the other hand, have the largest panicle width and bigger seed size, but they have a smaller number of tillers and productive tillers. Occurrences belonging to group 4 take more days to flower in the post-rainy season. Lastly, occurrences belonging to group 5 have the tallest plants after the rainy season and the second-tallest during the rainy season. Most have smaller numbers of tillers and a shorter panicle exertion. Their panicle length is the largest during and after the rainy season.

We generated classification models using the five groups described above, and based on the correlation between characterization and bioclimatic data, we predicted the unclassified data. The machine learning models reached an average accuracy of 55%: 89.9% for group 1; 38.1% for group 2; 23.2% for group 3; 14.8% for group 4; and 33.6% for group 5.

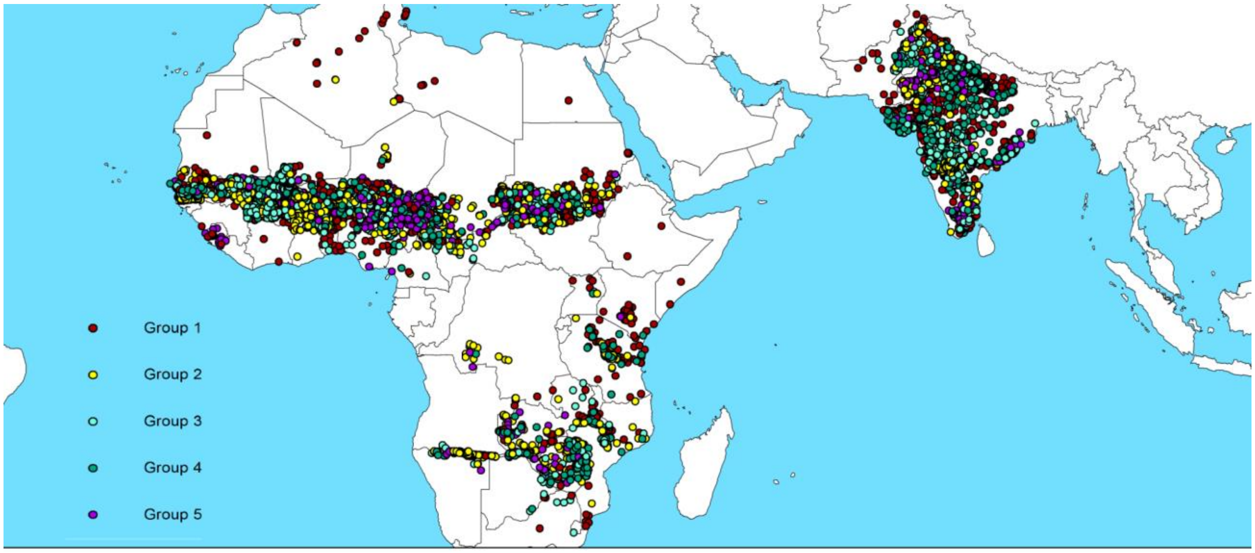

**Supplementary Fig. 4a:** All occurrences within the study region of pearl millet landrace groups.

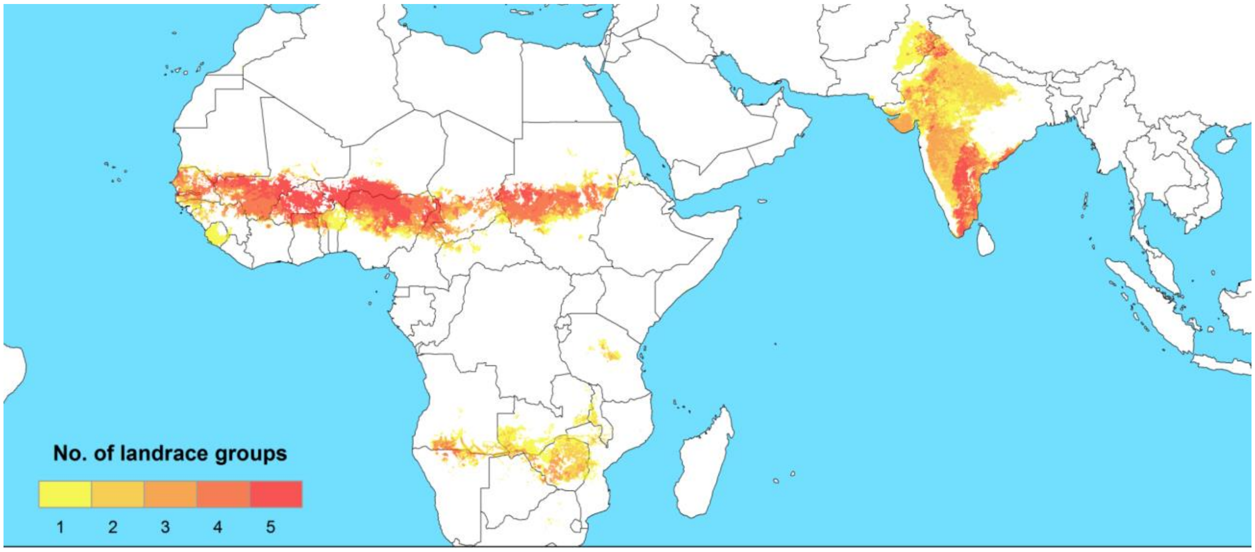

**Supplementary Fig. 4b:** Predicted distributions of pearl millet landrace groups.

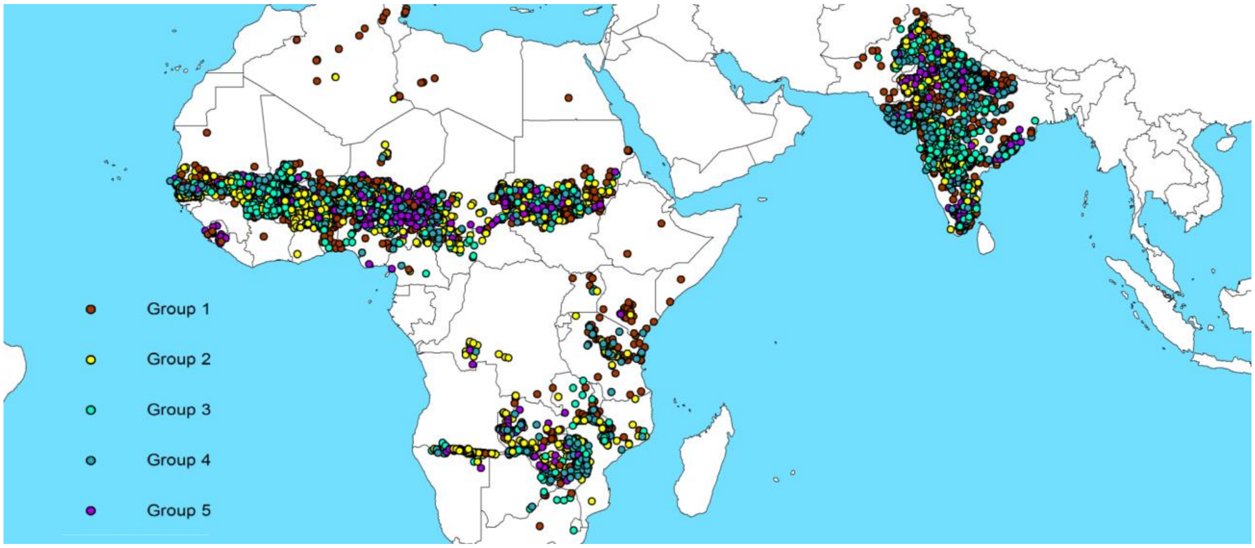

**Supplementary Fig. 4c:** Existing *ex situ* collection occurrences of pearl millet landrace groups.

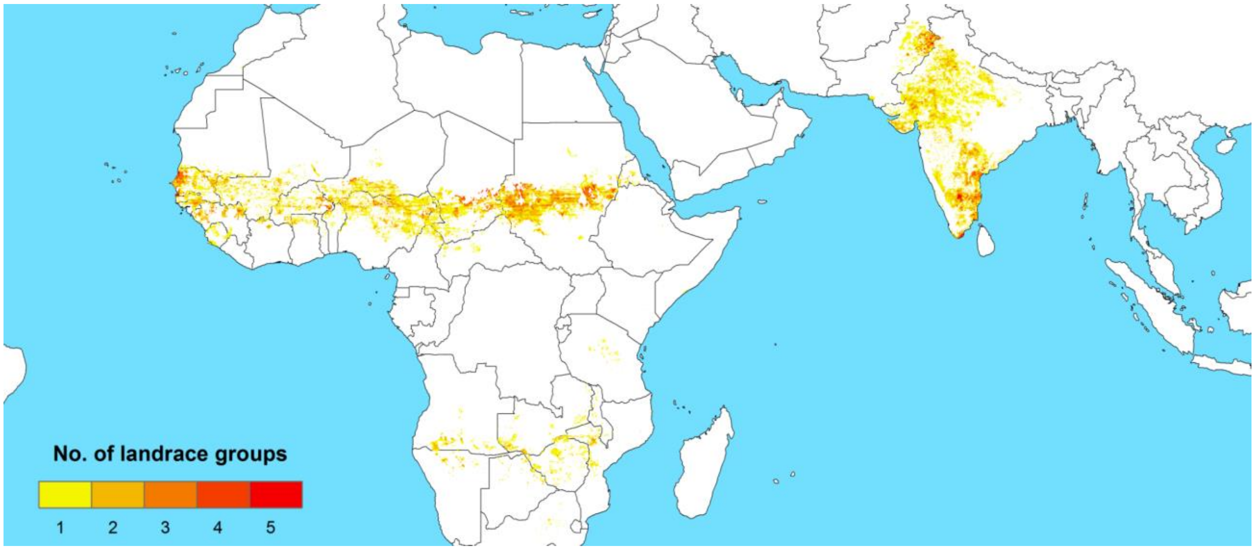

**Supplementary Fig. 4d:** Geographic gaps in the *ex situ* conservation of pearl millet landrace groups.

*African rice (*glaberrima*)*

African rice (*Oryza glaberrima* Steud.) was domesticated from the wild rice species *Oryza barthii* in West Africa approximately 3,000 years BP in the inner Niger Delta region in Mali [31]. Several studies have assessed the genetic diversity of *O. glaberrima* and have shown clear and marked differences between the analyzed accessions related to their geographic origin [32]. Ndjiondjop et al. (2018) [33] assessed the genetic variation, relatedness, and population structure of the *O. glaberrima* collection available at the AfricaRice institute through a diversity arrays technology-based sequencing (DArTseq) methodology. The authors found 5 clusters--K1, K2, K3, K4, and K5--that capture most of the genetic variation of the whole *O. glaberrima* collection conserved at the AfricaRice genebank. We based our training data on this classification system along with specific accession numbers gathered from published passport data.

We tested the environmental and socioeconomic signatures for DArTseq clusters K2, K4, and K5, combining groups K1 and K3 into a mixed group ("others") due to the low number of occurrences. Our attained average classification accuracy was 63.0% for K2, 69.2% for K4, 79.3% for K5, and 81.0% for the K1 and K3 mixed group, indicating that these groups have distinct environmental and socioeconomic signatures.

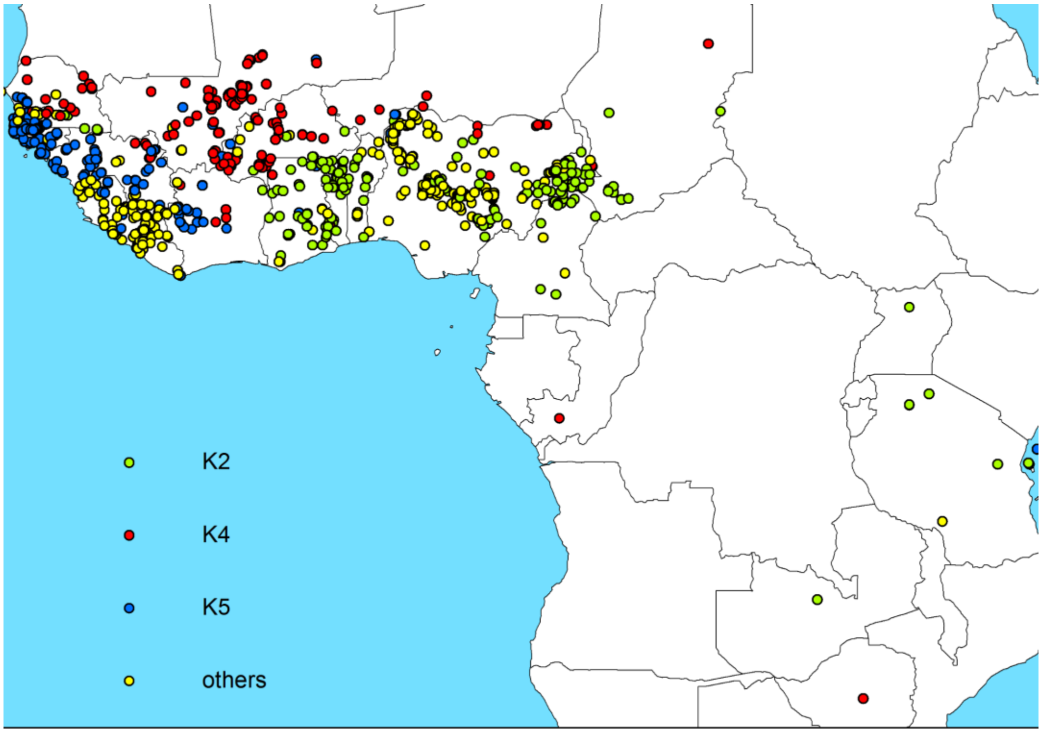


**Supplementary Fig. 5a:** All occurrences within the study region of African rice landrace groups.

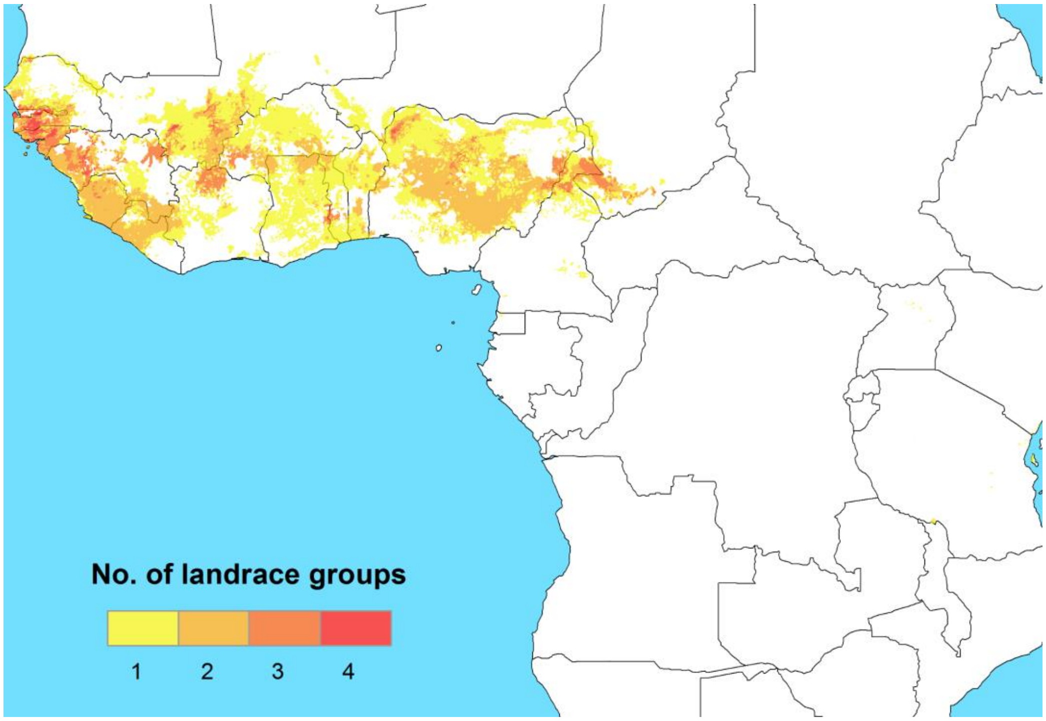


**Supplementary Fig. 5b:** Predicted distributions of African rice landrace groups.

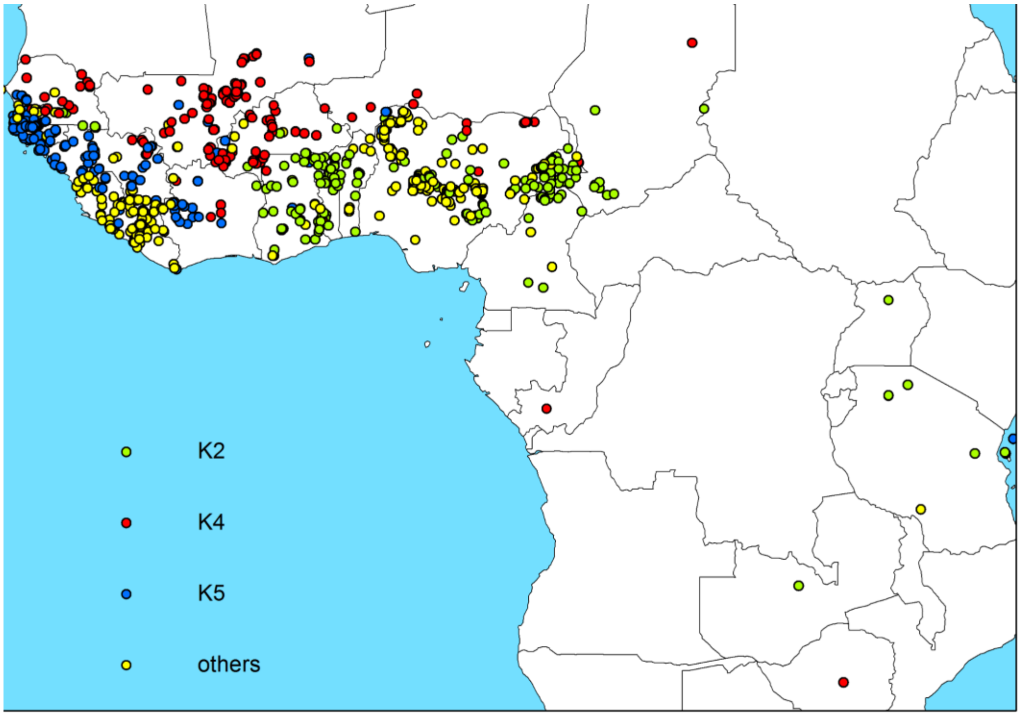


**Supplementary Fig. 5c**: Existing *ex situ* collection occurrences of African rice landrace groups.

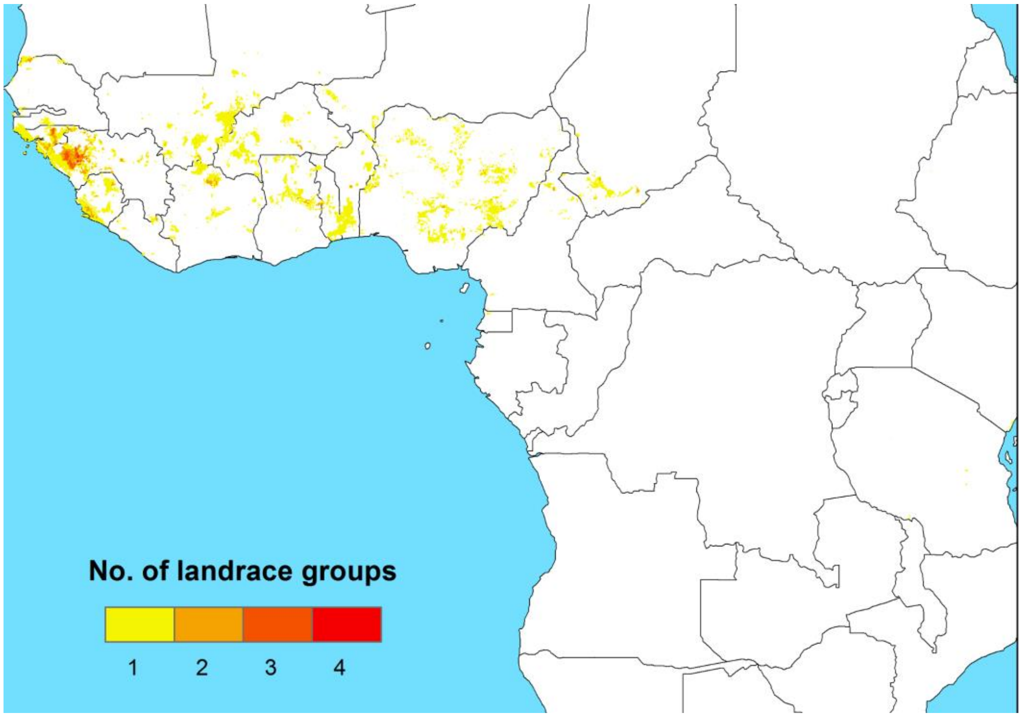


**Supplementary Fig. 5d**: Geographic gaps in the *ex situ* conservation of African rice landrace groups.

*Asian rice*

Asian rice (*Oryza sativa* L.) was domesticated from *O. rufipogon* Griff. through two separate domestication events. First, *O. sativa* group *japonica* was domesticated ~7,000 years ago in the Yangtze Basin of southern China. *Oryza sativa* group *indica* was subsequently domesticated in the Ganges plains of India ∼4,500 years ago [34].

Garris et al. (2005) [35], genotyped a sample of 234 accessions of rice using 169 nuclear simple sequence repeats and two chloroplast loci. The data were analyzed to resolve the genetic structure and interpret the evolutionary relationships between groups. Five distinct major groups were detected: indica, aus, aromatic, temperate japonica, and tropical japonica rices, although lower levels of differentiation were detected in the pairwise comparisons of temperate and tropical japonica. Based on this classification and using passport data from research literature [36,37], we tested the environmental and socioeconomic signatures for indica, japonica (together), aus, and aromatic rices. Our average classification accuracy was 70.8% (69.0% for aus, 73.9% for indica, 65.6% for japonica, and 33.0% for aromatic rice), indicating that these groups have distinct environmental and socioeconomic signatures.

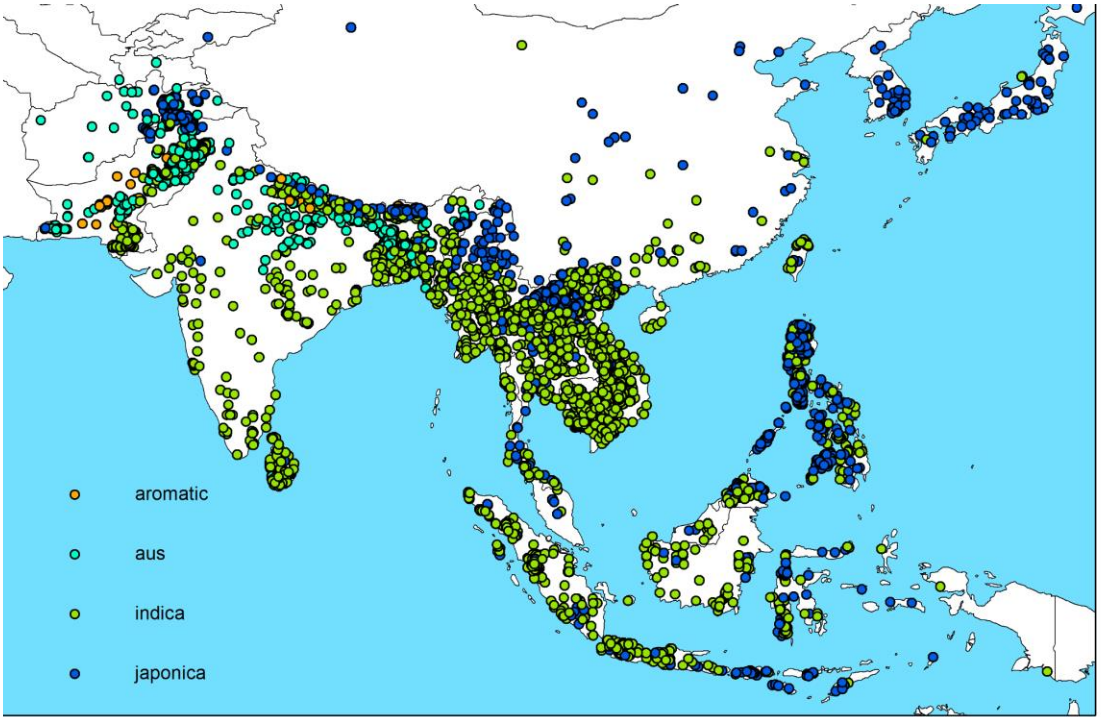

**Supplementary Fig. 6a**: All occurrences within the study region of Asian rice landrace groups.

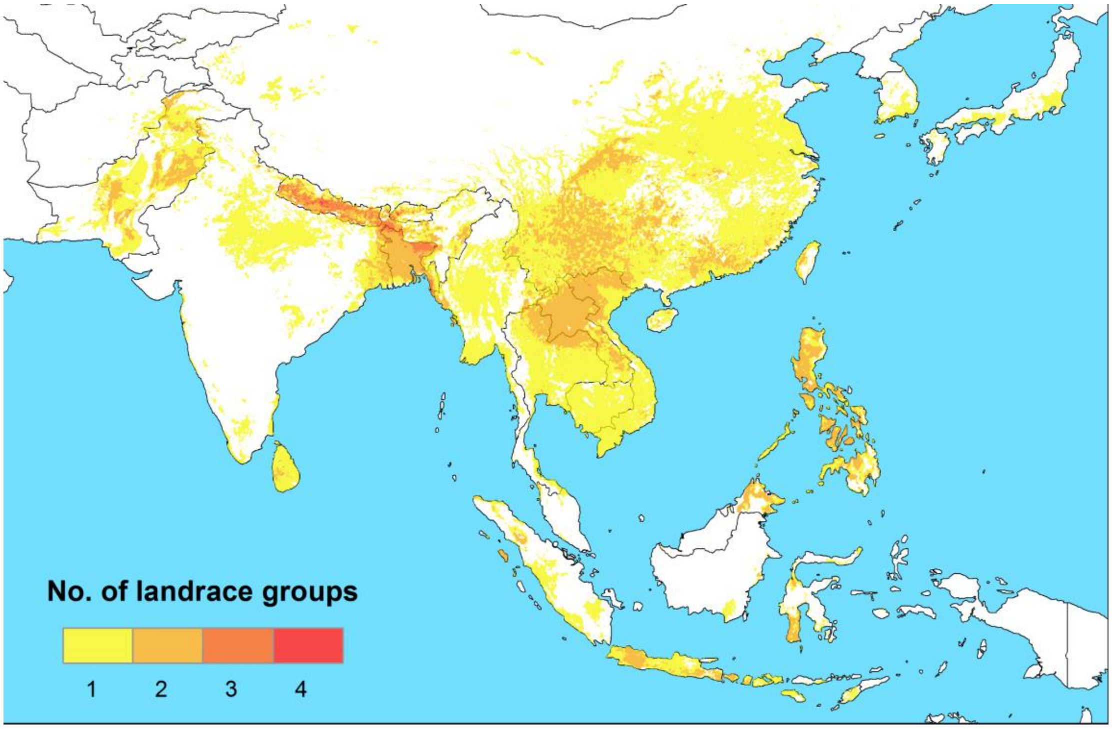

**Supplementary Fig. 6b**: Predicted distributions of Asian rice landrace groups.

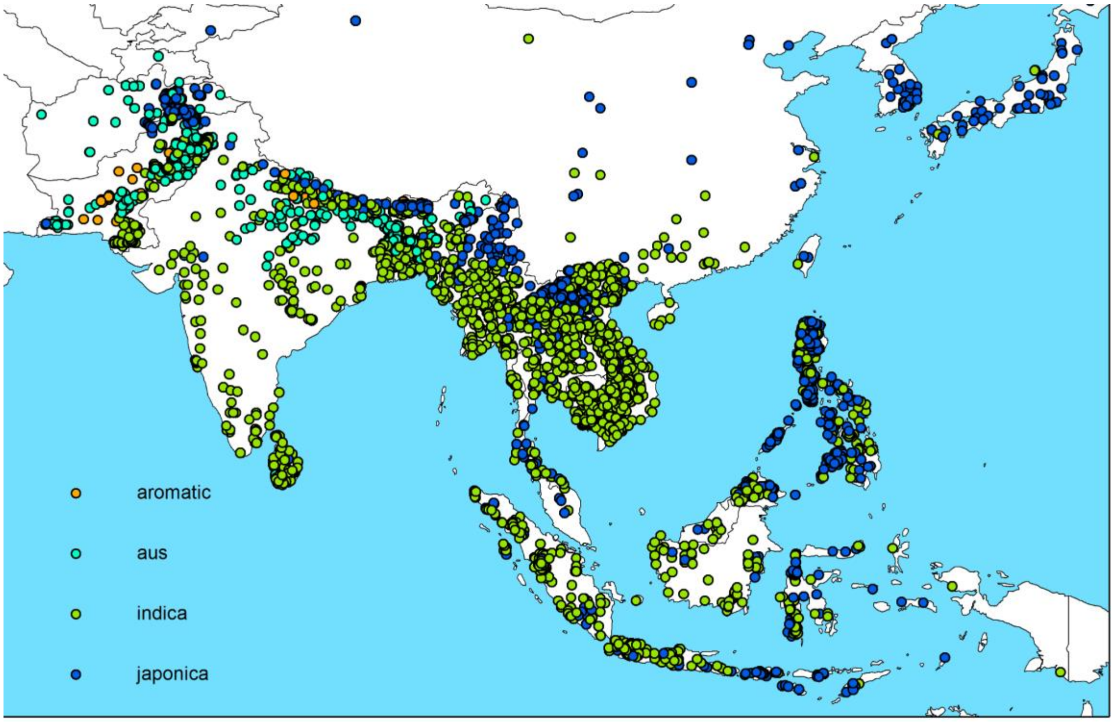


**Supplementary Fig. 6c**: Existing *ex situ* collection occurrences of Asian rice landrace groups.

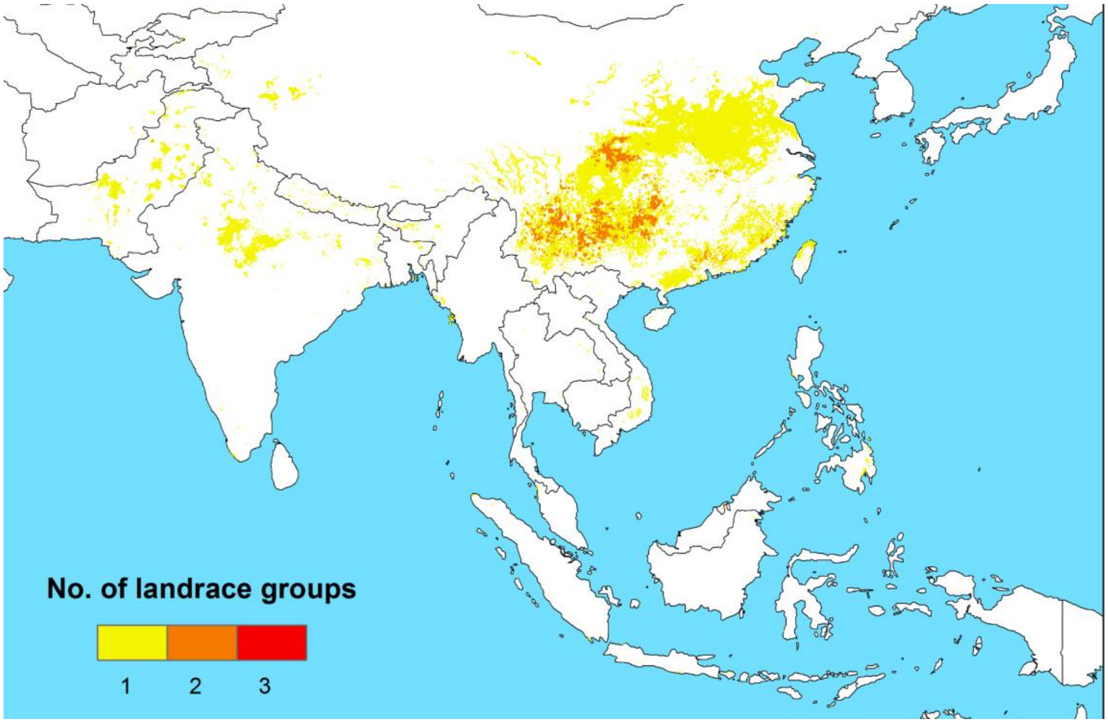


**Supplementary Fig. 6d**: Geographic gaps in the *ex situ* conservation of Asian rice landrace groups.

*Sorghum*

*Sorghum bicolor* (L.) Moench. is an important crop in semiarid and arid regions because it can sustain high yields where precipitation is scant, so it has become a major cereal crop for diverse peoples living in these ecological regions in Sub-Saharan Africa and South Asia. Sorghum is considered to have been domesticated from *Sorghum bicolor* subsp. *verticilliflorum* (Steud.) de Wet ex Wiersema & J. Dahlb. in East Africa around 5,000 years ago [39]. The crop was then dispersed across Africa, South Asia, the Middle East, and East Asia. Sorghum samples from these regions display a wide range of morphological and physiological traits useful for crop improvement [40,41].

Harlan et al. (1972) [41] identified five basic infraspecific groups ("races") of sorghum according to different morphological characteristics of the mature heads, panicles, and spikelets of the crop: bicolor, guinea, caudatum, kafir, and durra. Due to natural hybridization between races, ten intermediate races may also be recognized.

We generated classification models from ICRISAT genebank accession data labeled with the five main races and predicted unclassified accessions. Occurrences labeled with the intermediate races were not predicted due to the small quantity of data for each one. The accuracy of our models was 80.8% on average (31.3% for bicolor, 89.2% for guinea, 73.5% for caudatum, 60% for kafir, and 82.9% for durra).

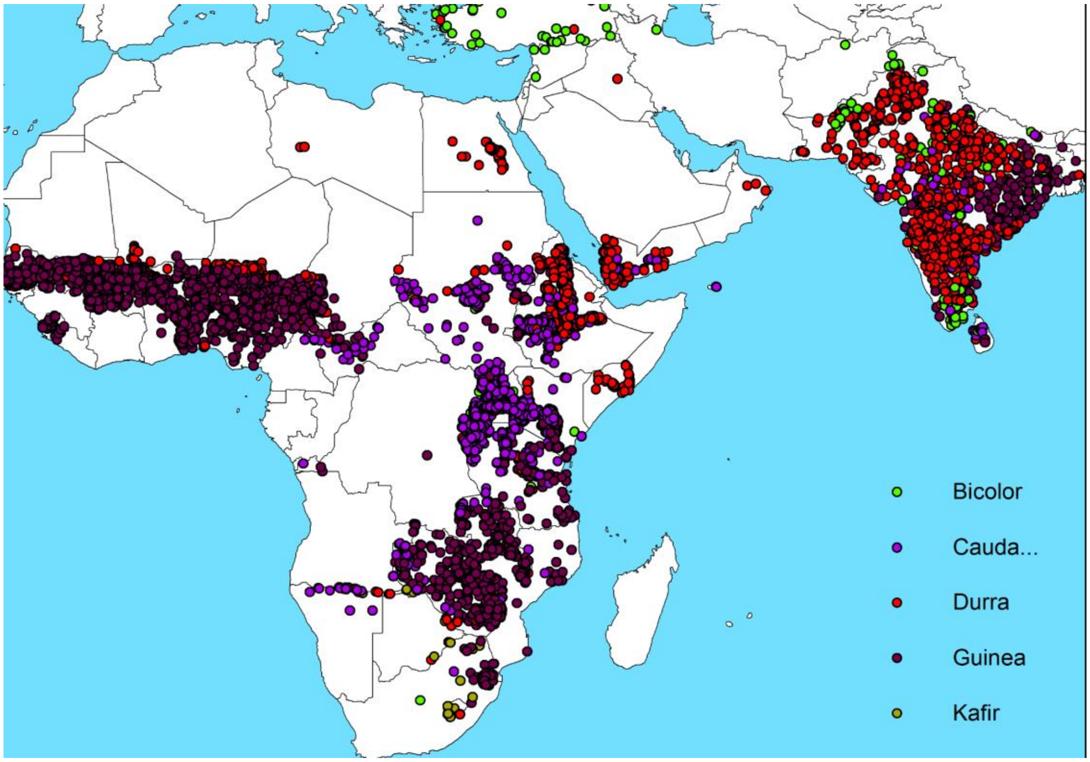


**Supplementary Fig. 7a:** All occurrences within the study region of sorghum landrace groups.

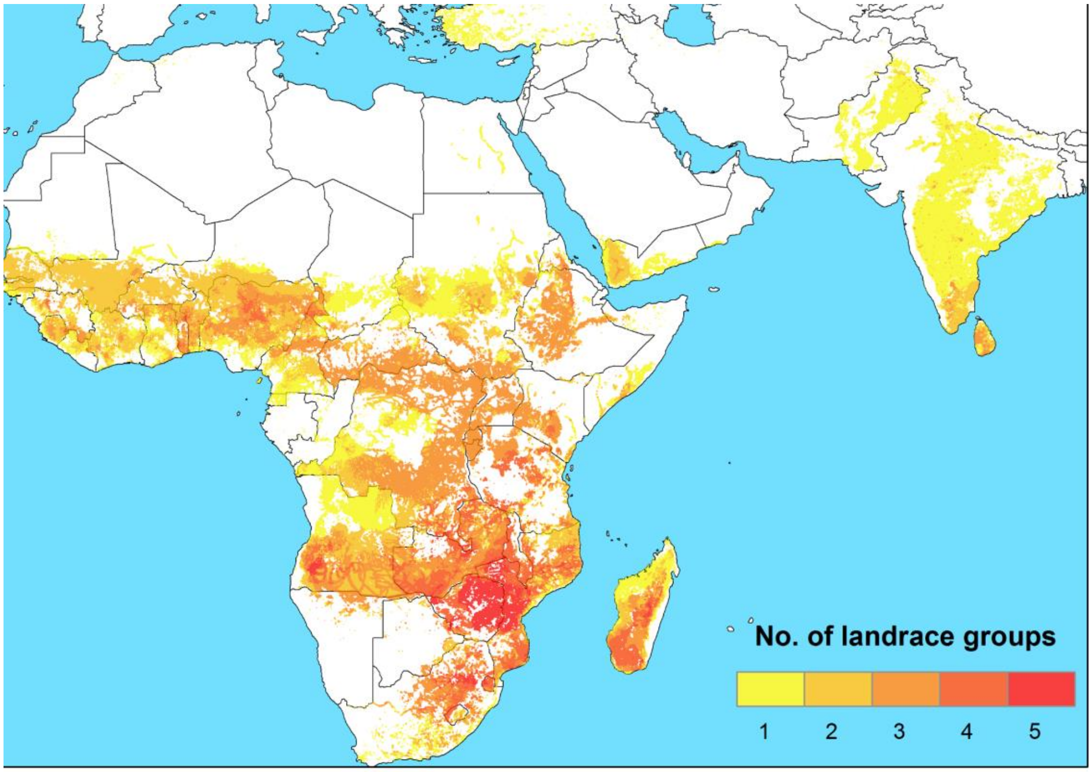


**Supplementary Fig. 7b:** Predicted distributions of sorghum landrace groups.

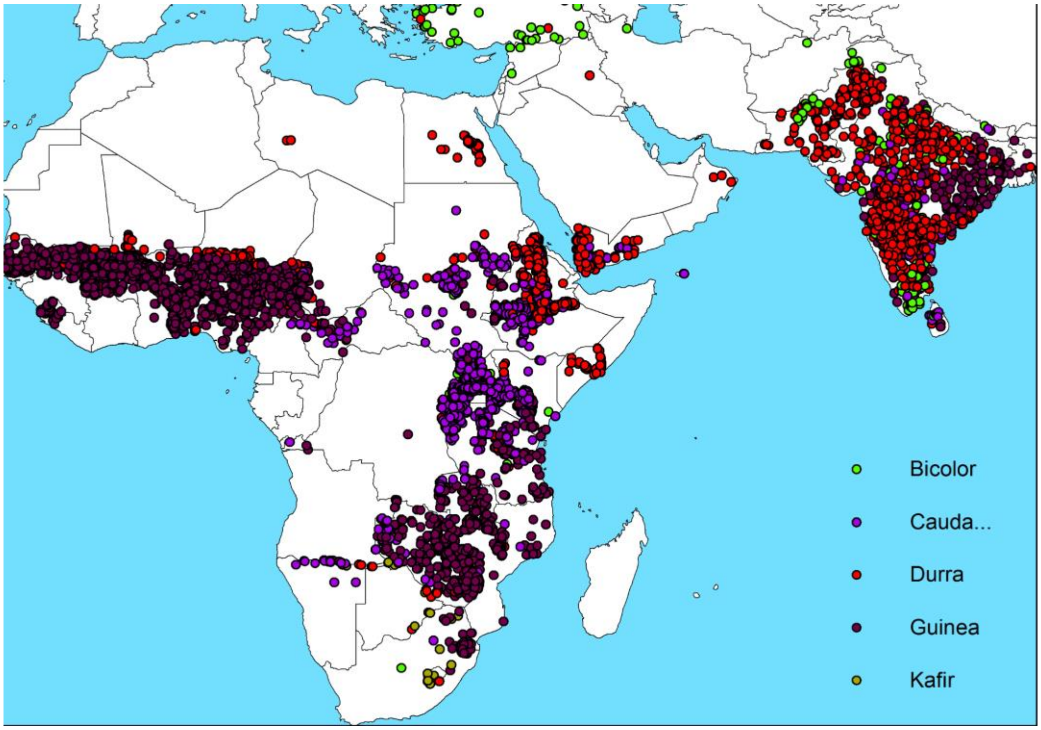


**Supplementary Fig. 7c:** Existing *ex situ* collection occurrences of sorghum landrace groups.

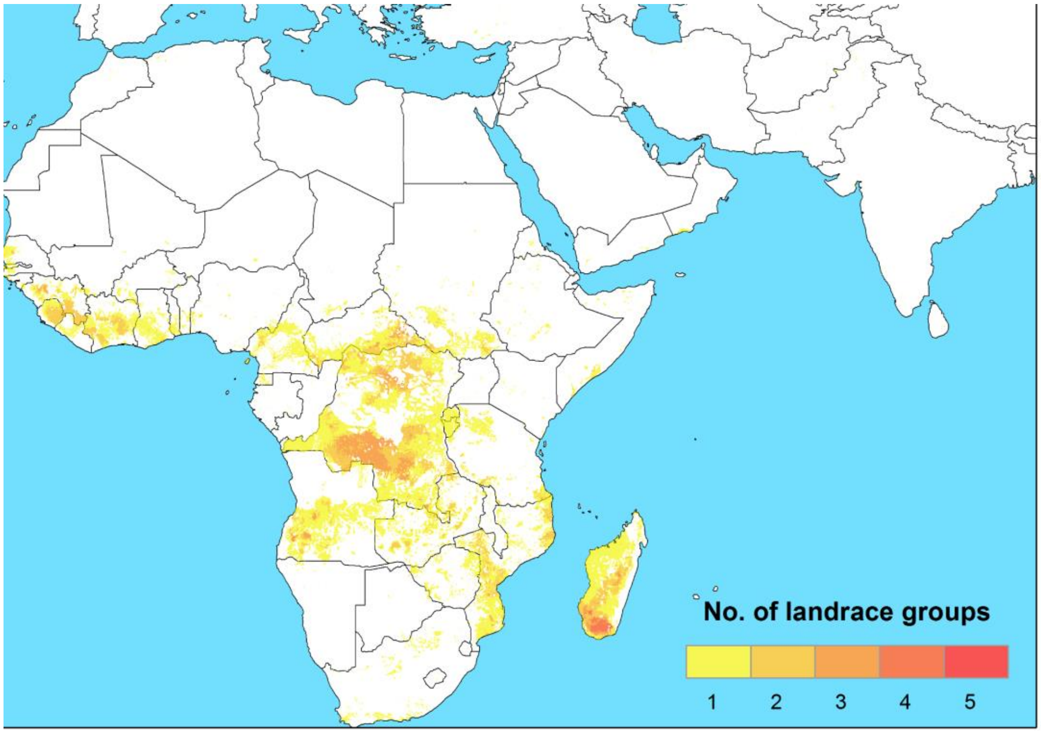


**Supplementary Fig. 7d**: Geographic gaps in the *ex situ* conservation of sorghum landrace groups.

*Bread and durum wheat*

Wheat was domesticated more than 10,000 years ago in the Fertile Crescent and subsequently in West Asia [42-44]. Tetraploid durum wheat (T*riticum turgidum* L. subsp. *durum* [Desf.] van Slageren) was domesticated from its wild progenitor (*T. turgidum* L. subsp. *dicoccon* (Schrank) Thell.). Bread wheat (*T. aestivum* L.) was then domesticated following a polyploidisation event between the domesticated tetraploid *T. turgidum* subsp. *dicoccon* and the wild diploid relative *Aegilops tauschii Coss*, approximately in northern Iran.

Because infraspecific taxonomic structures for durum and bread wheat are not widely agreed or implemented in occurrence data, each crop was analyzed for infraspecific structure. Sansaloni et al. (2020) [45] assigned bread wheat accessions from the CIMMYT genebank database into 11 genetic groups. The environmental responses of the identified genetic clusters were assessed using published passport data and climate and socioeconomic variables. For durum wheat, Kehel et al. (unpublished data) tested a range of different genetic clusters using the ICARDA genebank database. The genetic representation that best responded to environmental signals was the following: group 1 (Ethiopia); group 2 (Syria and Lebanon) group 3 (North Africa); and group 4 (Turkey).

We also tested groups based on environmental characters (bioclimatic and socioeconomic data), using a principal component analysis and a hierarchical clustering of components to obtain the optimal number of groups. From this analysis, three environmental clusters of each wheat crop were identified. The biplot figures below show the grouping of the accessions and the influence upon them from environmental and socioeconomic variables.

Bread wheat

- Group 1: Lowest mean temperature during the coldest quarter, highest precipitation during the driest quarter, and lowest maximum temperature during the coldest month.
- Group 2: Lowest precipitation during the warmest quarter, longest distance to rivers, and the most irrigation.
- Group 3: Highest precipitation.

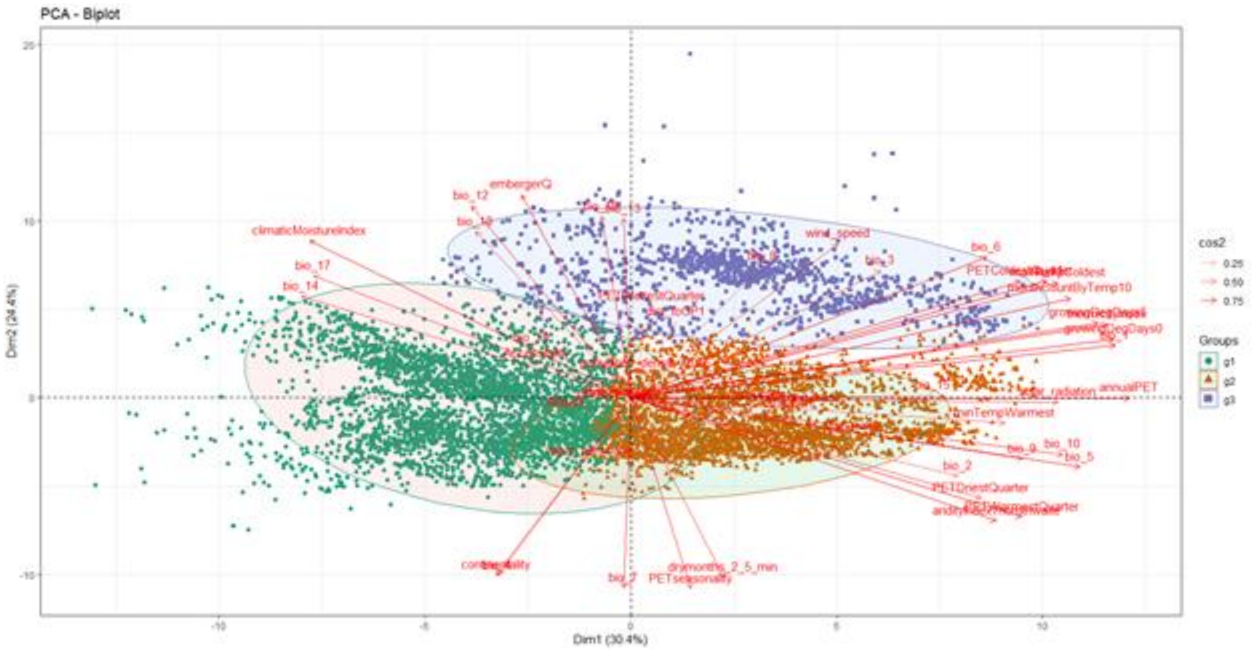


Durum wheat

- Group 1: Highest precipitation during the driest month, highest precipitation during the driest quarter, and lowest minimum temperature during the coldest month.
- Group 2: Lowest precipitation during the driest quarter, and the most irrigation.
- Group 3: Highest locations and the most precipitation.

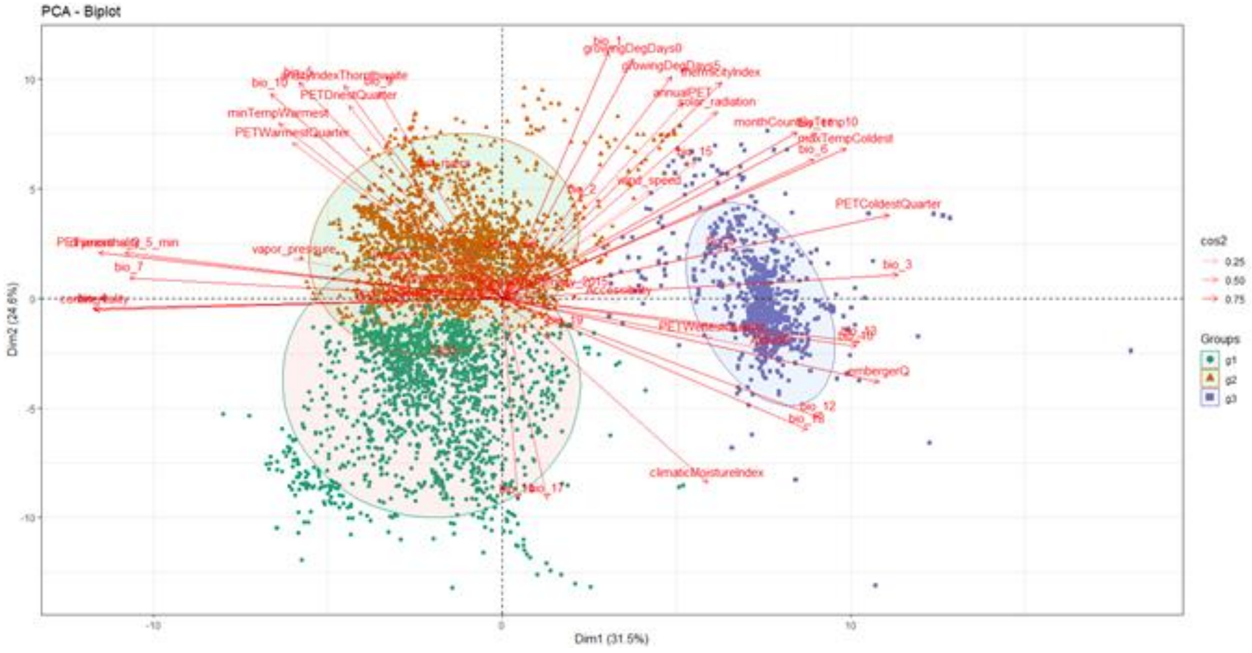

Testing the accuracy of the groupings, the environmental groups were more accurately classified than the genetic groups. Therefore, the environmental groups were the final landrace structures used to perform the modeling and conservation gap analysis.

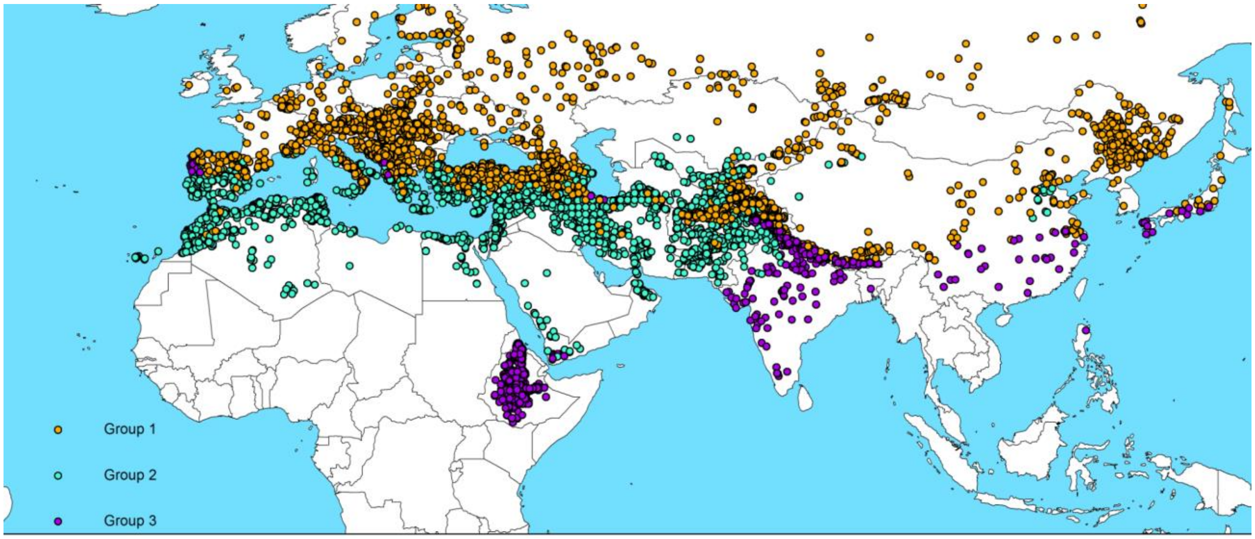


**Supplementary Fig. 8a:** All occurrences within the study region of bread wheat landrace groups.

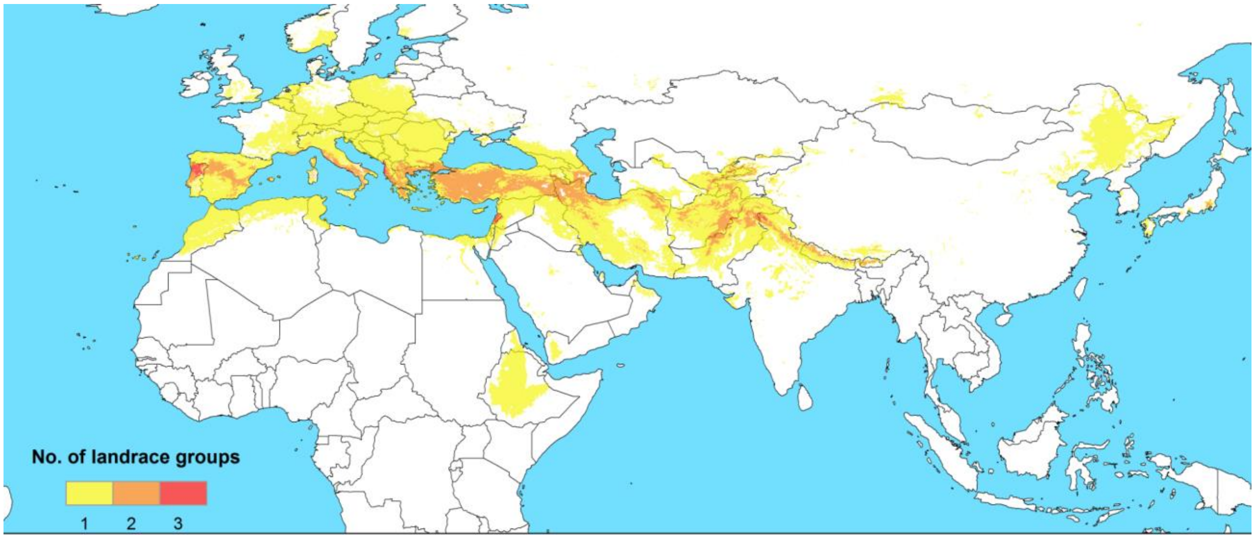


**Supplementary Fig. 8b:** Predicted distributions of bread wheat landrace groups.

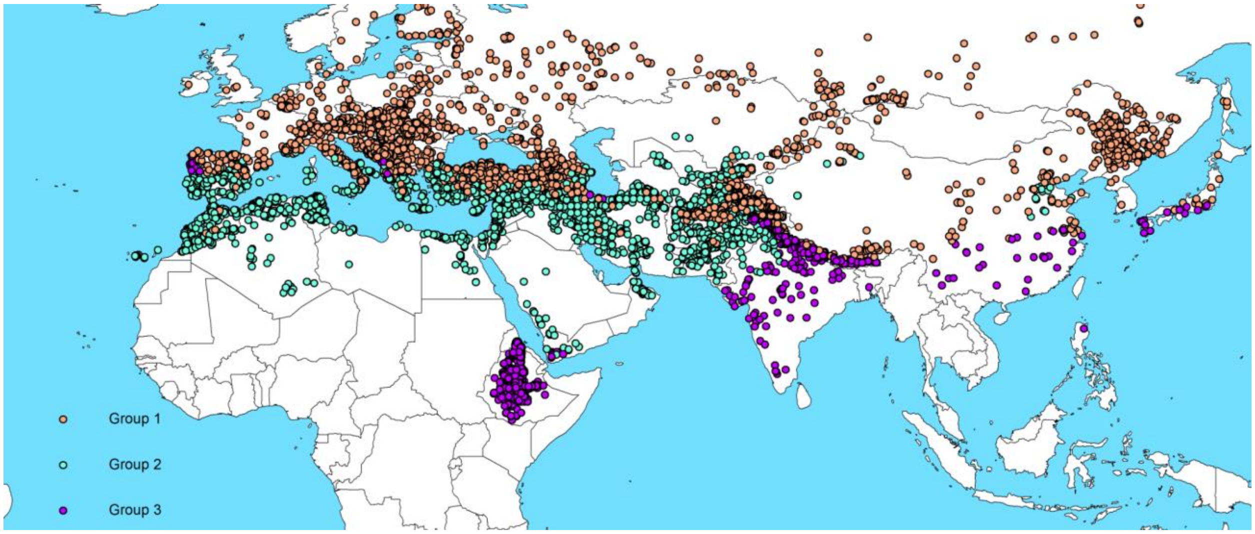

**Supplementary Fig. 8c:** Existing *ex situ* collection occurrences of bread wheat landrace groups.

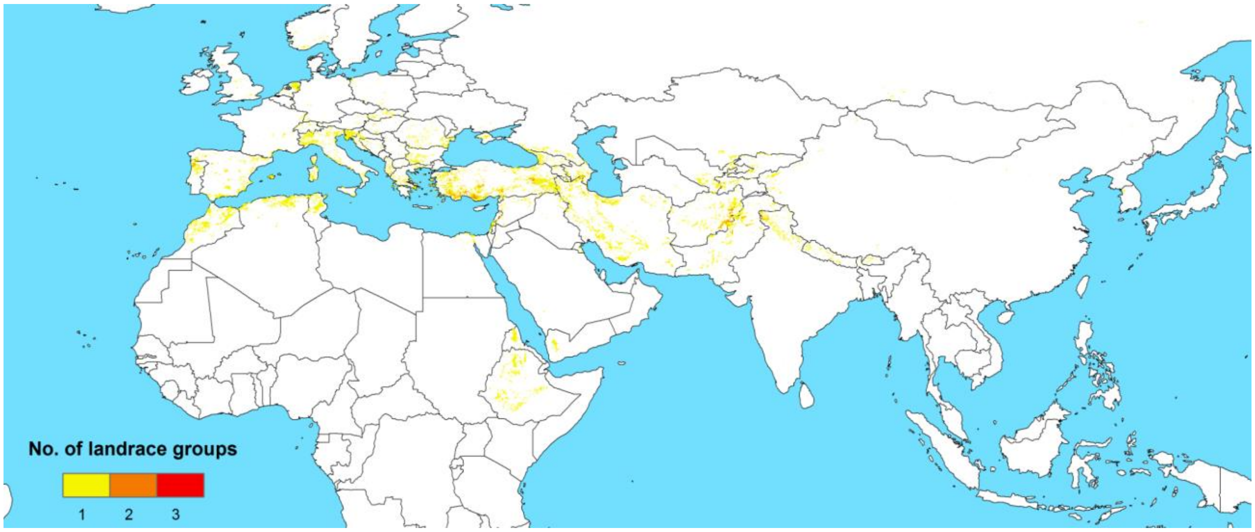

**Supplementary Fig. 8d**: Geographic gaps in the *ex situ* conservation of bread wheat landrace groups.

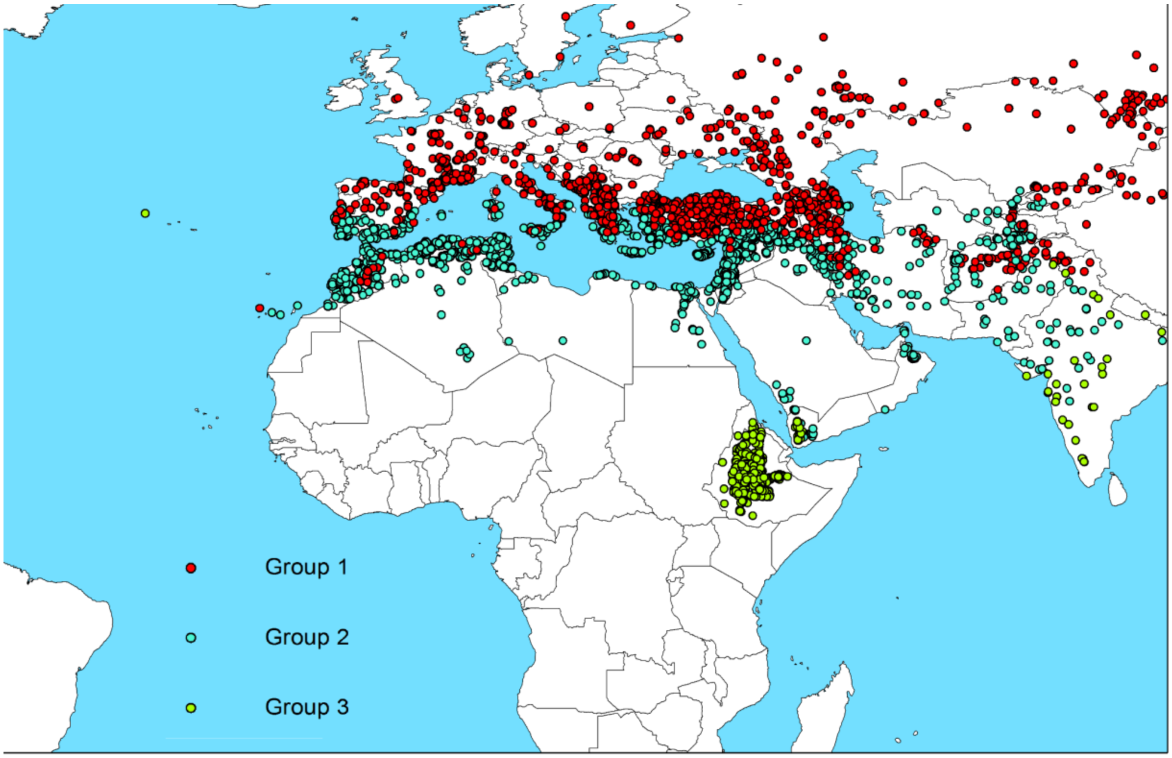


**Supplementary Fig. 9a:** All occurrences within the study region of durum wheat landrace groups.

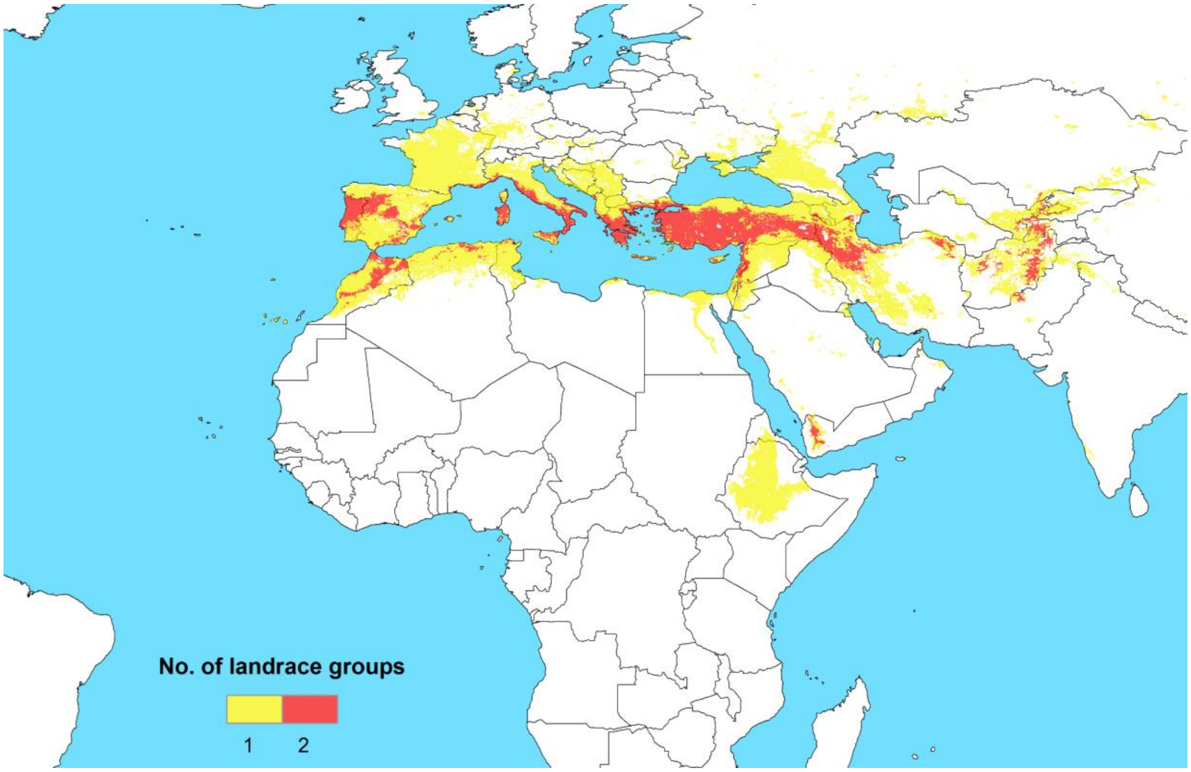


**Supplementary Fig. 9b**: Predicted distributions of durum wheat landrace groups.

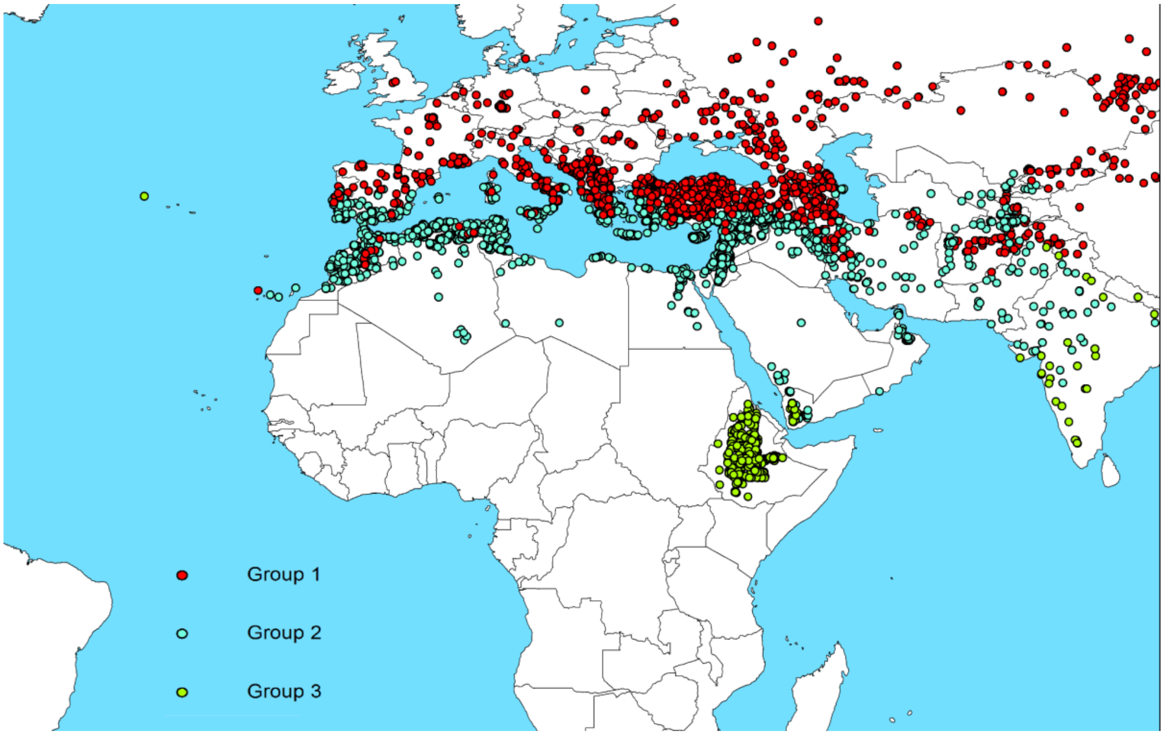

**Supplementary Fig. 9c:** Existing *ex situ* collection occurrences of durum wheat landrace groups.

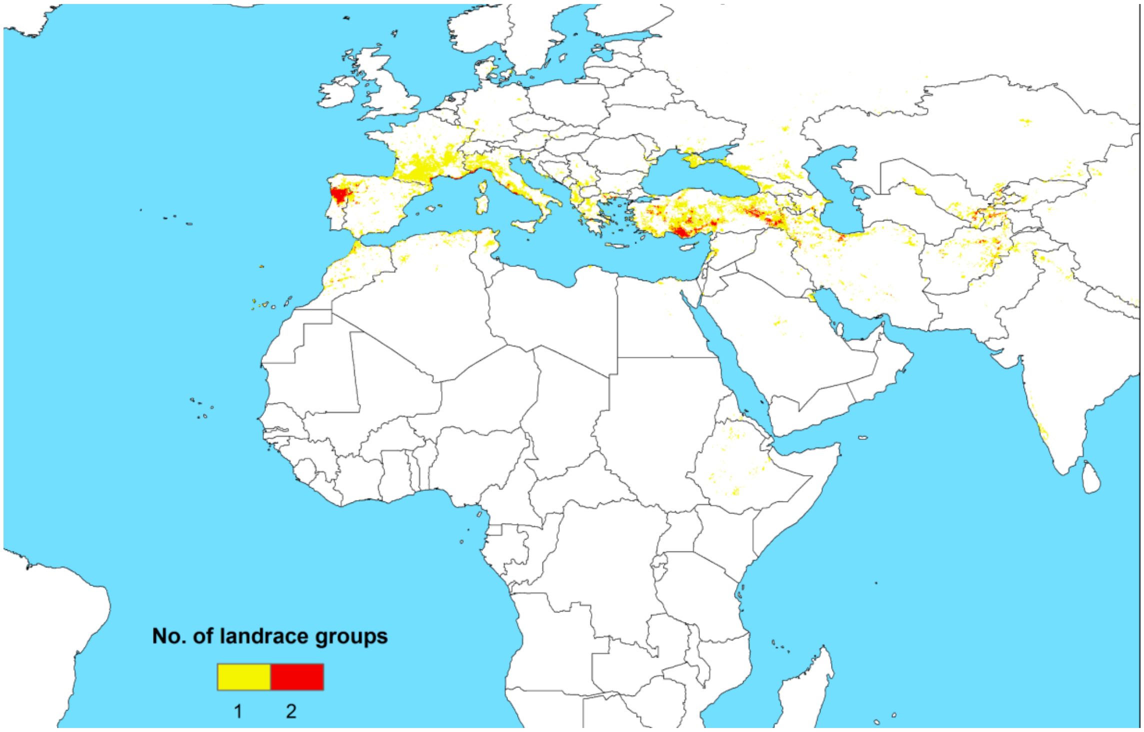

**Supplementary Fig. 9d**: Geographic gaps in the *ex situ* conservation of durum wheat landrace groups.

*Pulses*

*Chickpea*

There are two major cultivated/commercial types of chickpea (*Cicer arietinum* L.), which differ based on seed morphology: desi and kabuli [46]. Desi seeds are angular and dark-colored, while kabuli seeds are "owl-shaped" and beige-colored. Desi types also typically have pink flowers, while white flowers typically characterize kabuli types.

Beyond these major groups, our literature review indicated that the diversity of chickpeas has not been comprehensively characterized based on the rather limited availability of detailed phenotypic information for key adaptive traits. We therefore used information about seed shape and flower color to classify groups as follows:

- If the occurrence's seed is angularly shaped and its flower is pink, then the occurrence is *desi*.
- If the occurrence's seed is owl-shaped and its flower is beige, orange, white, or whitish pink then, the occurrence is *kabuli*.

Classification models were generated using the resulting groups in order to predict the unlabeled occurrences from other databases. Machine learning models classified the occurrences with an 83.2% accuracy, 80.97% for desi and 85.7% for kabuli.

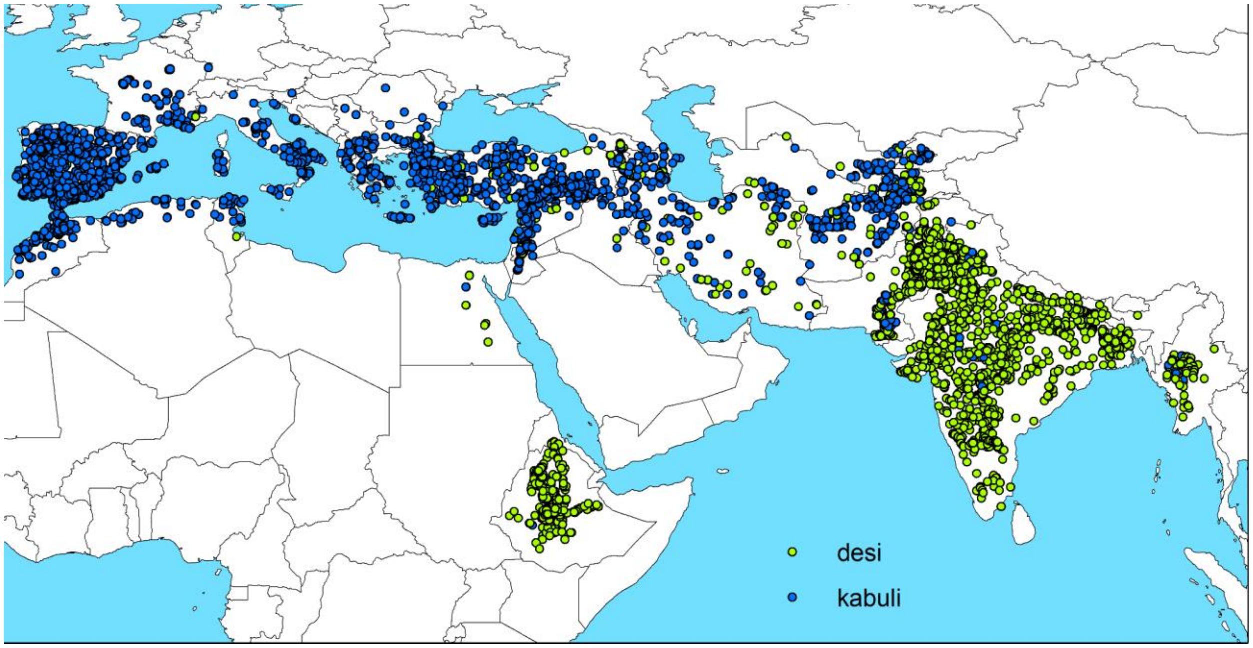


**Supplementary Fig. 10a:** All occurrences within the study region of chickpea landrace groups.

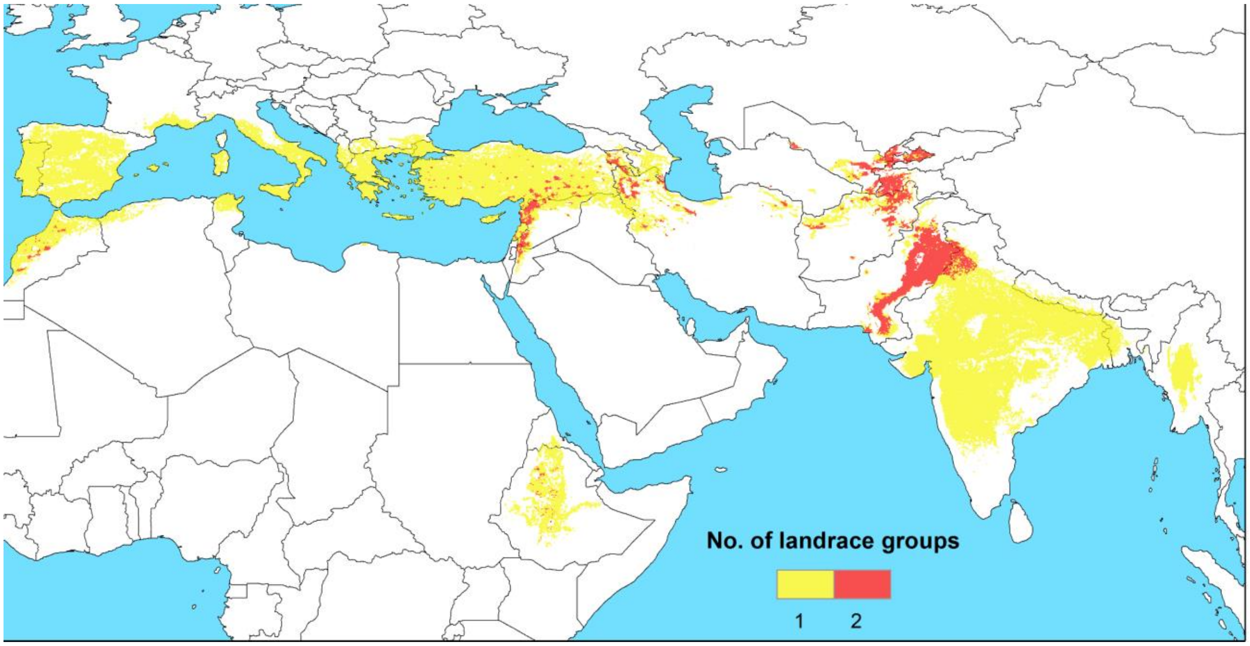


**Supplementary Fig. 10b**: Predicted distributions of chickpea landrace groups.

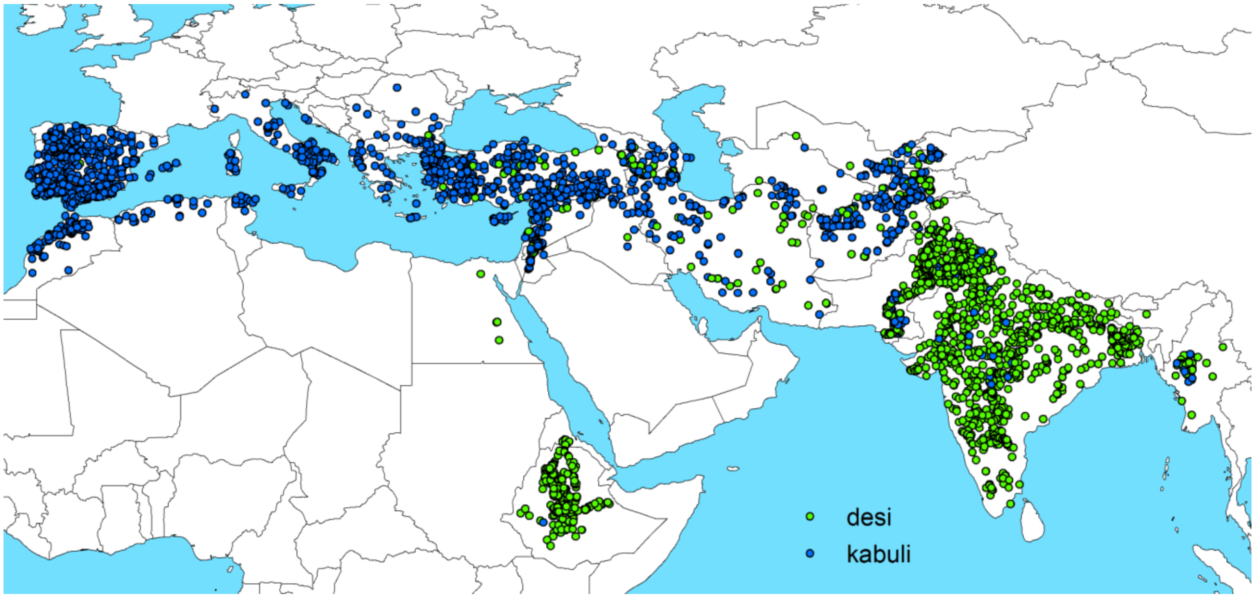


**Supplementary Fig. 10c:** Existing ex situ collection occurrences of chickpea landrace groups.

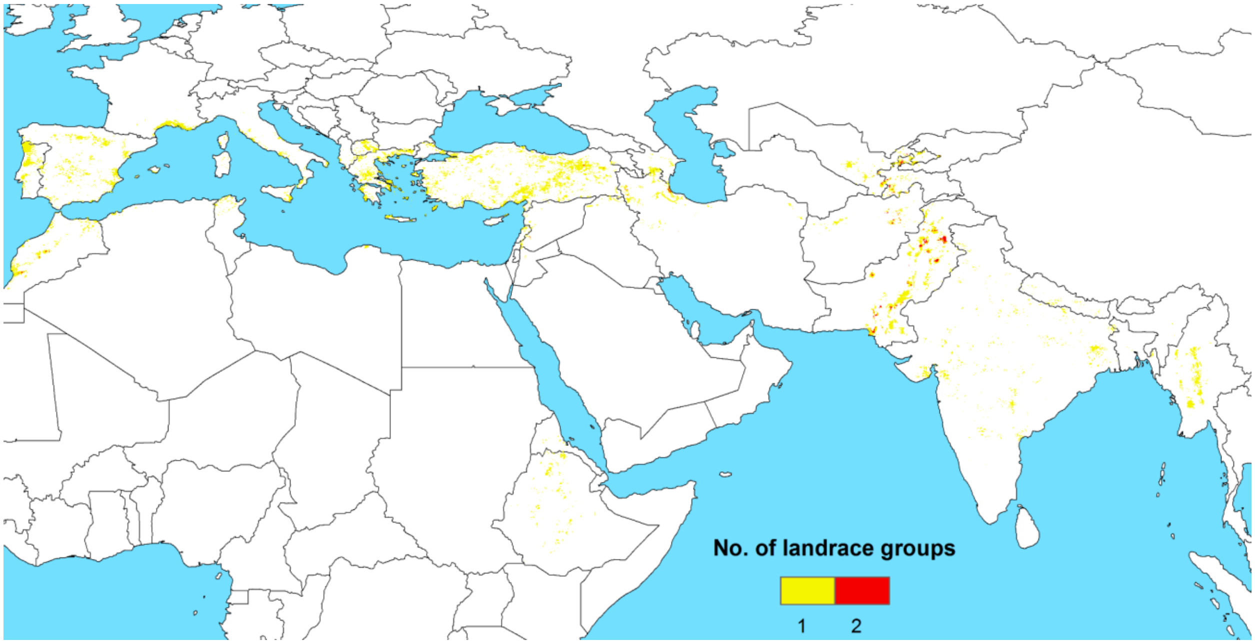


**Supplementary Fig. 10d**: Geographic gaps in the *ex situ* conservation of chickpea landrace groups.

*Common bean*

A single major infraspecific classification system is widely accepted for common bean (*Phaseolus vulgaris* L.) landraces [47]. This system, first proposed by Singh et al. (1991) [48], classifies beans into two genepools: Andean and Mesoamerican. The Andean genepool, derived from a domestication event thought to have occurred in the vicinity of Peru, Chile, and Bolivia, is composed of typically larger-seeded genotypes. The Mesoamerican genepool--derived from a domestication event involving the same wild progenitor species, *Phaseolus vulgaris* L. var. *aborigineus* (Burkart) Baudet, but different populations in Mexico and Central America--is typically composed of smaller-seeded genotypes [48,49]. These genepools are further classified into races according to morphological and genetic information. The Andean genepool is divided into the races Chile, Nueva Granada, and Peru, whereas the Mesoamerican genepool contains the races Guatemala, Durango–Jalisco, and Mesoamerica [48,50-54].

As records were not always already specified to genepool or race, we tested a variety of available characterization data associated with these divisions, including seed protein type; seed weight, color, shape, and brightness; and landrace names [50,51,55-79] . Based on published studies and the availability of accession-level data with geographic coordinates, we ultimately based our training data on genepool designations given in the CIAT accessions dataset along with specific accession numbers gathered from the literature we reviewed [47].

Our average, classification accuracy at the genepool level was 86%--88.3% for Andean and 85.0% for Mesoamerican landraces--indicating that these two genepools have distinct environmental and socioeconomic signatures, with Mesoamerican beans present in lower, drier, and hotter places than Andean beans. At the race level, the classification accuracy was considerably lower--58.5% as a mean across all races--and hence determined to be much less predictable. Based on these results, we concluded that the genepool level was the most appropriate for the distribution modeling and conservation gap analysis.

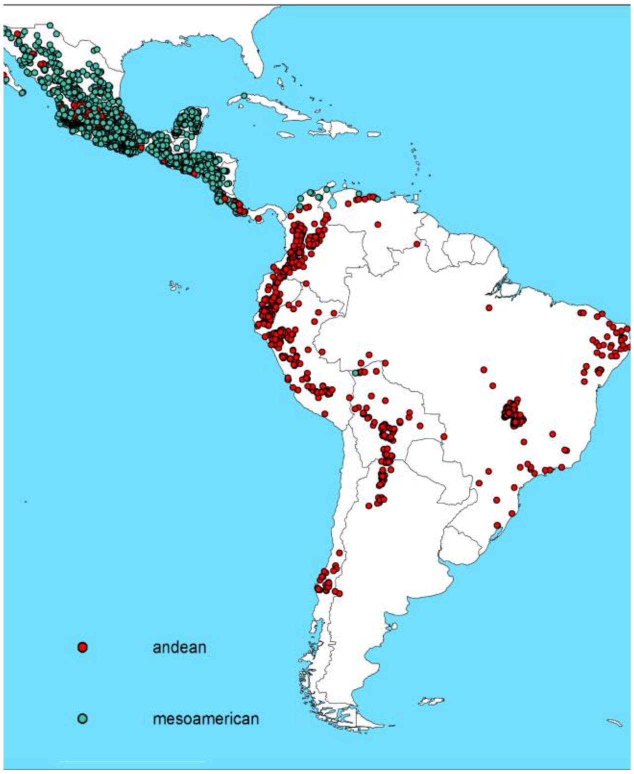


**Supplementary Fig. 11a:** All occurrences within the study region of common bean landrace groups.

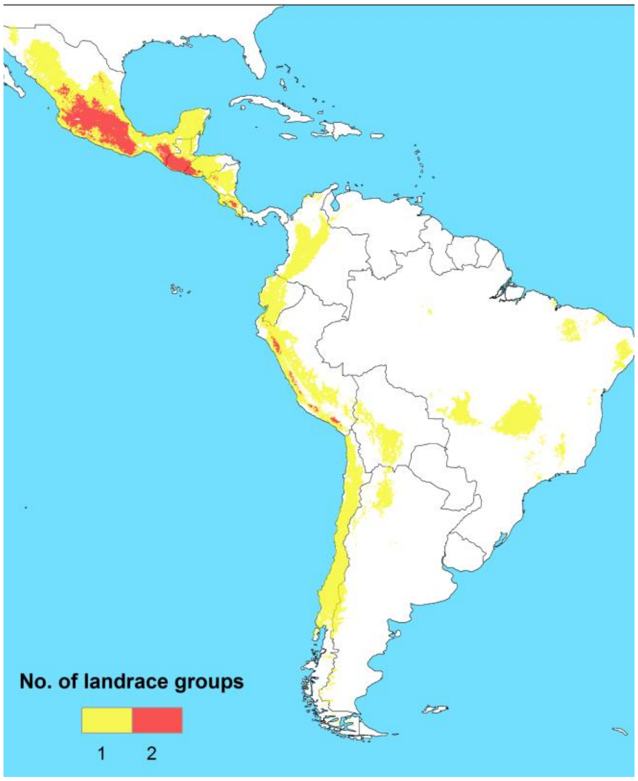


**Supplementary Fig. 11b**: Predicted distributions of common bean landrace groups.

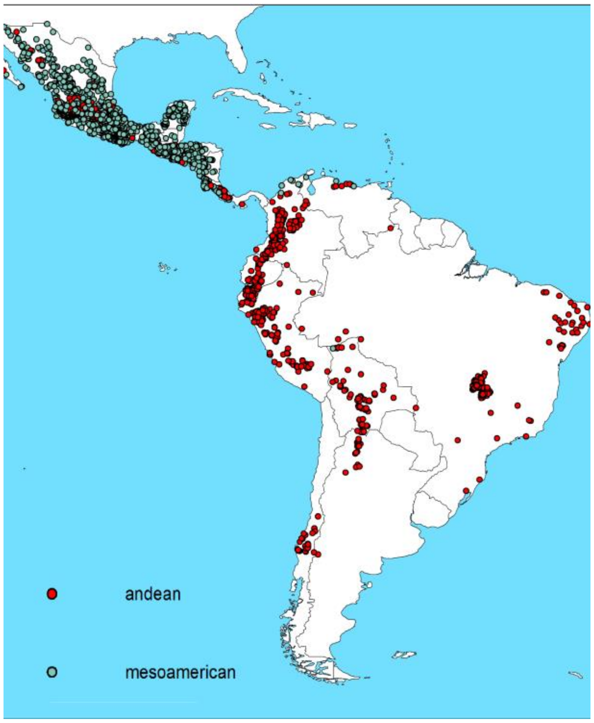


**Supplementary Fig. 11c**: Existing *ex situ* collection occurrences of common bean landrace groups.

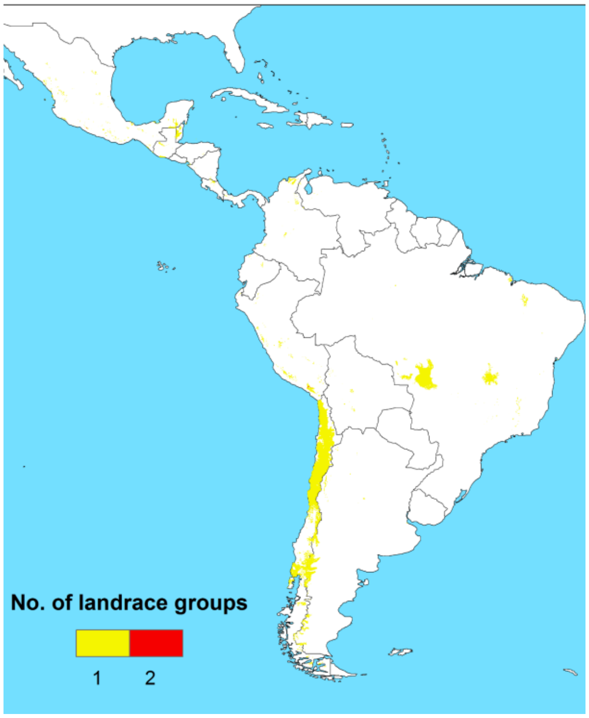


**Supplementary Fig. 11d**: Geographic gaps in the *ex situ* conservation of common bean landrace groups.

*Cowpea*

According to Pasquet (2000) [80], who classified cowpea types based on morphological, allozyme, and chloroplast DNA data, there are five cultivar-groups of cowpeas: textilis, sesquipedalis, melanophthalmus, biflora, and unguiculata. These can be grouped into two categories based on number of ovules: biflora and melanophthalmus have a low number of ovules, while unguiculata and sesquipedalis display a high number of ovules. Each group can be further divided into subgroups [80]. However, available occurrences rarely contained such labeling, constraining the ability to classify landraces based on these structures.

For this reason, and taking into account that most recent classifications were done on the basis of morphological characteristics, characterization data from IITA was used to perform a cluster analysis, in order to understand how the occurrences may be grouped according to seed characteristics. Two major clusters emerged. The variables that contributed the most to construct the groups included terminal leaflet length, number of locules per pod, terminal leaflet width, pod length, and days to the first ripe pod.

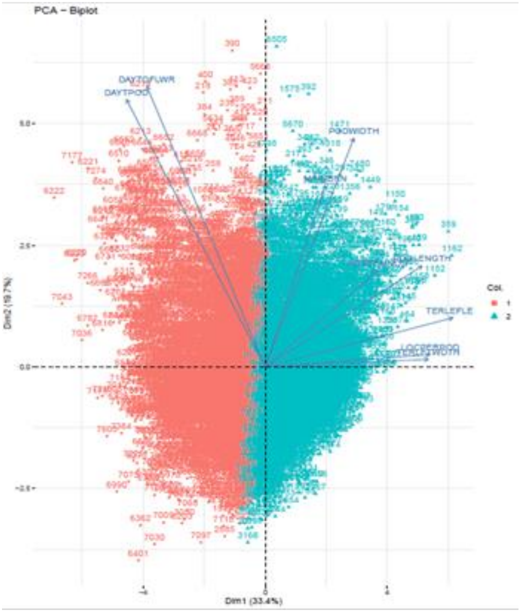


As the biplot shows, occurrences belonging to group 1 take more days from planting to the first ripe pod and from planting to the first flowering. Those characteristics describe most accessions in this group. Their pod sizes in terms of width and length and terminal leaflet sizes in terms of width and length are smaller. They usually have fewer locules per pod. On the other hand, occurrences belonging to group 2 have terminal leaflets of larger size and more locules per pod. Most have larger pods and more main stem nodes. The machine learning models classified an average of 79.6% of the occurrences into the correct group: 83.4% for group 1 and 74.8% for group 2.

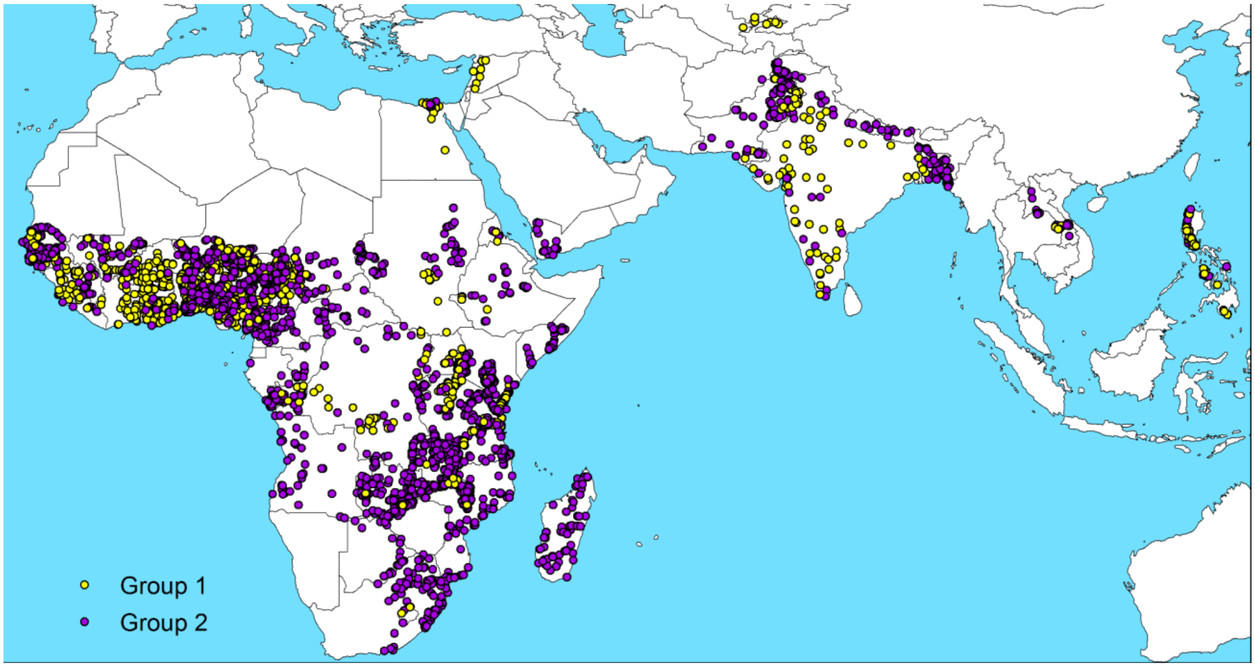


**Supplementary Fig. 12a**: All occurrences within the study region of cowpea landrace groups.

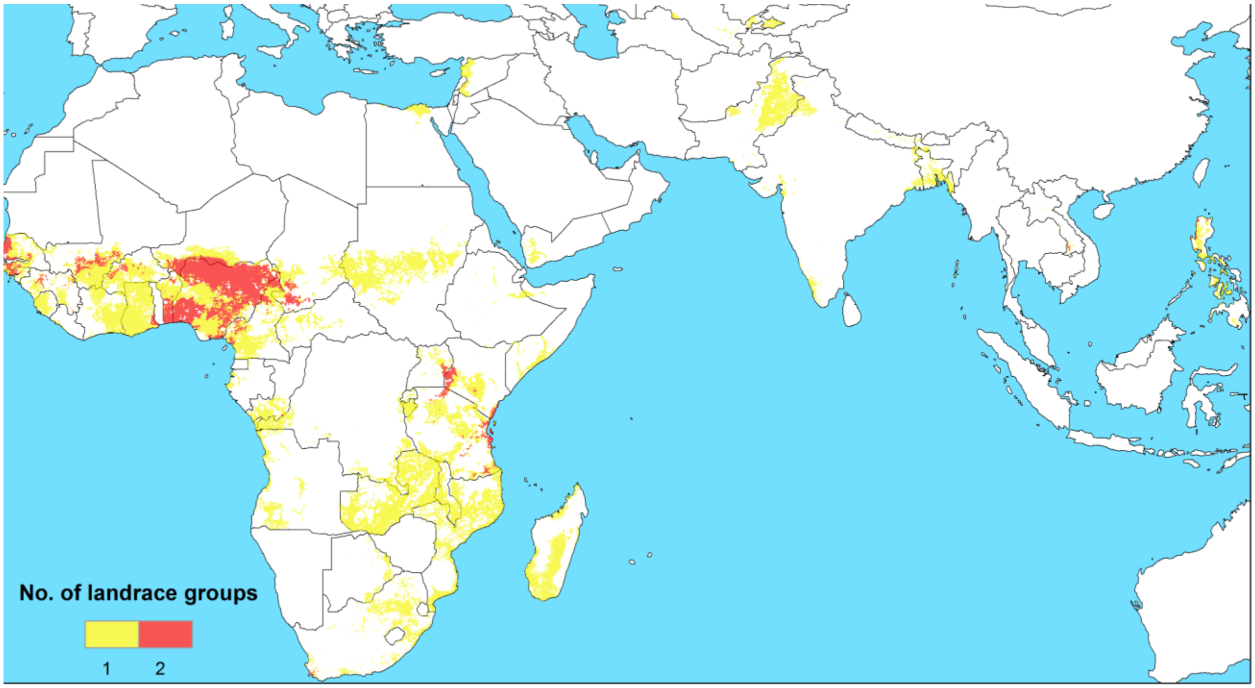


**Supplementary Fig. 12b**: Predicted distributions of cowpea landrace groups.

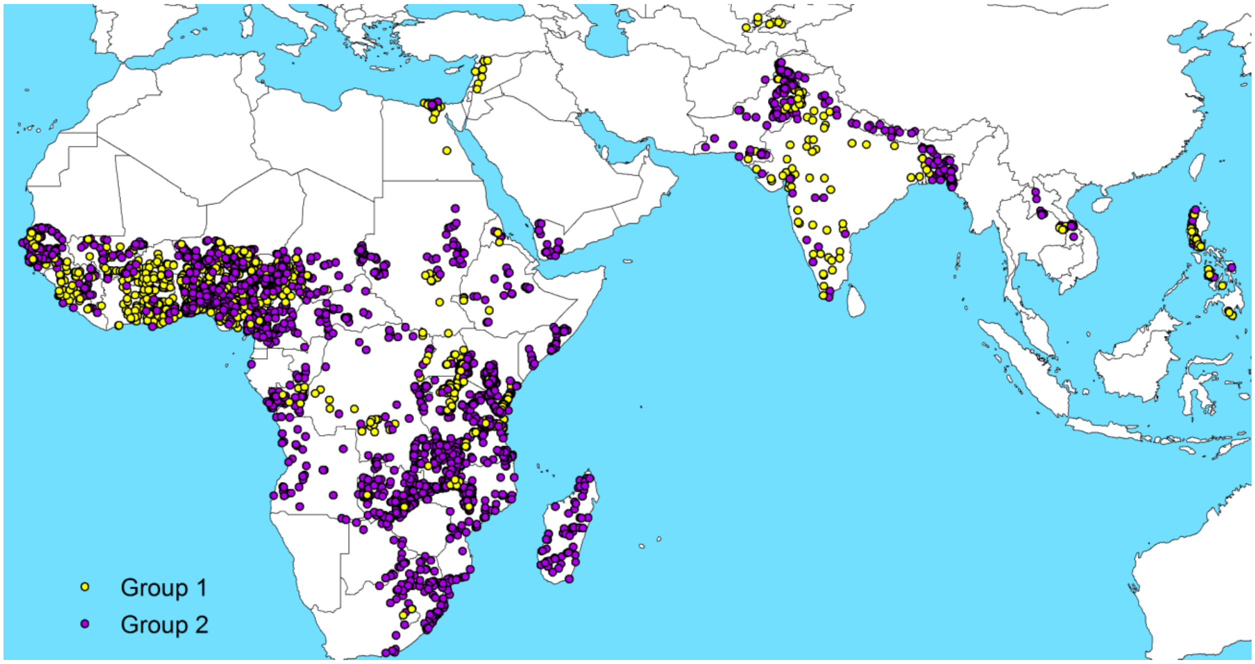


**Supplementary Fig. 12c**: Existing *ex situ* collection occurrences of cowpea landrace groups.

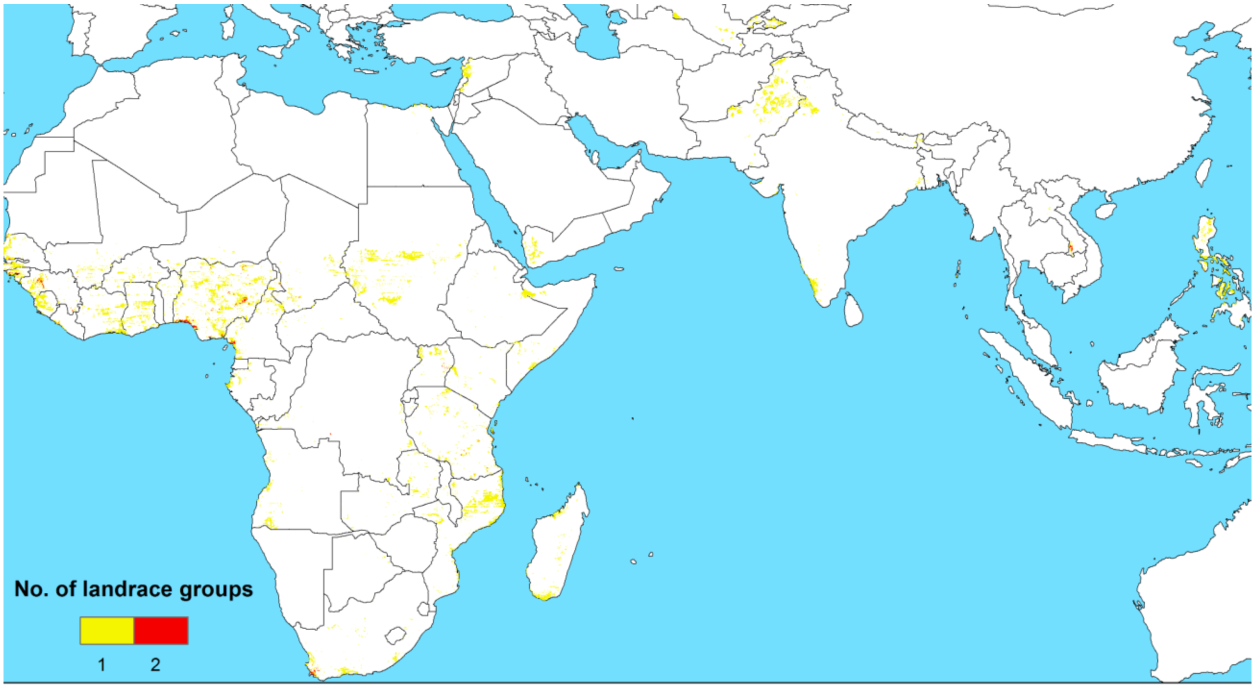


**Supplementary Fig. 12d**: Geographic gaps in the *ex situ* conservation of cowpea landrace groups.

*Faba bean*

Faba bean (*Vicia faba* L.), also called fava bean and broad bean, was domesticated around 8000 BCE somewhere between the eastern Mediterranean and Afghanistan and spread to Central Europe and Russia through Asia Minor [81]. A more precise origin of *Vicia faba* L. is still unknown, and no wild progenitor has been clearly identified [82]. The progenitor may be extinct, and its original habitat may no longer exist due to degradation and destruction [83].

Various studies have identified or proposed taxonomic classifications for faba beans based on morphological seed characteristics and ecogeographic conditions [84-86]. However, based on our literature search there is no strong consensus about these classifications. Further, and more of a constraint, these classifications were very rarely attached to available occurrence data. We therefore performed the modeling and conservation gap analysis for the pulse at the crop level.

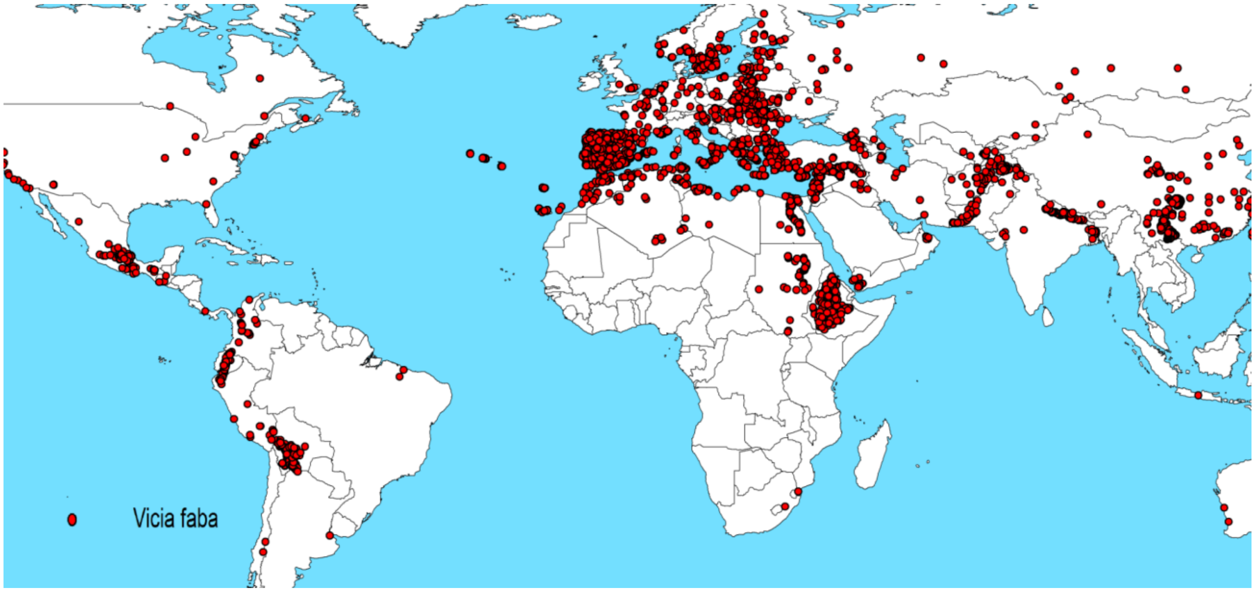


**Supplementary Fig. 13a:** All occurrences within the study region of faba bean landraces.

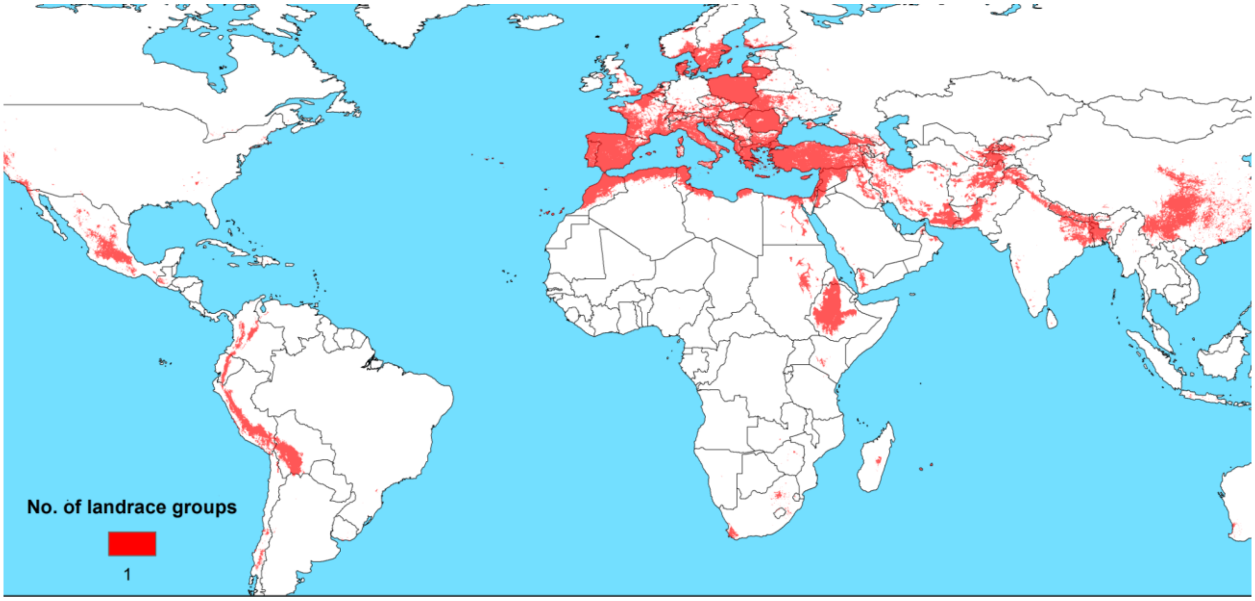


**Supplementary Fig. 13b**: Predicted distributions of faba bean landraces.

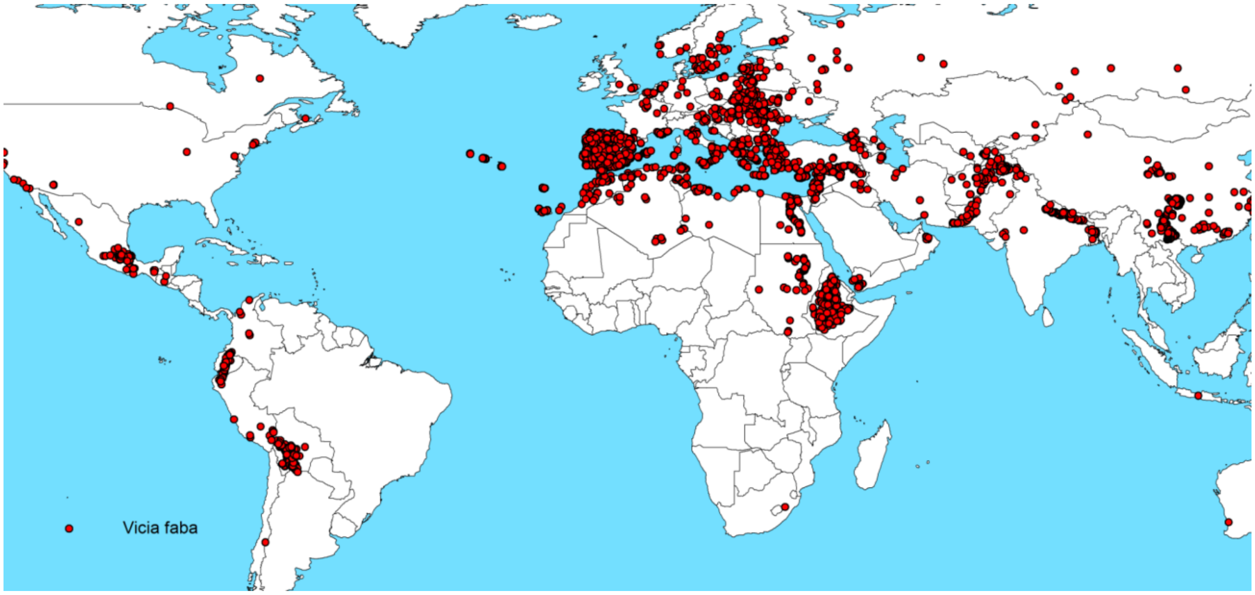


**Supplementary Fig. 13c**: Existing *ex situ* collection occurrences of faba bean landraces.

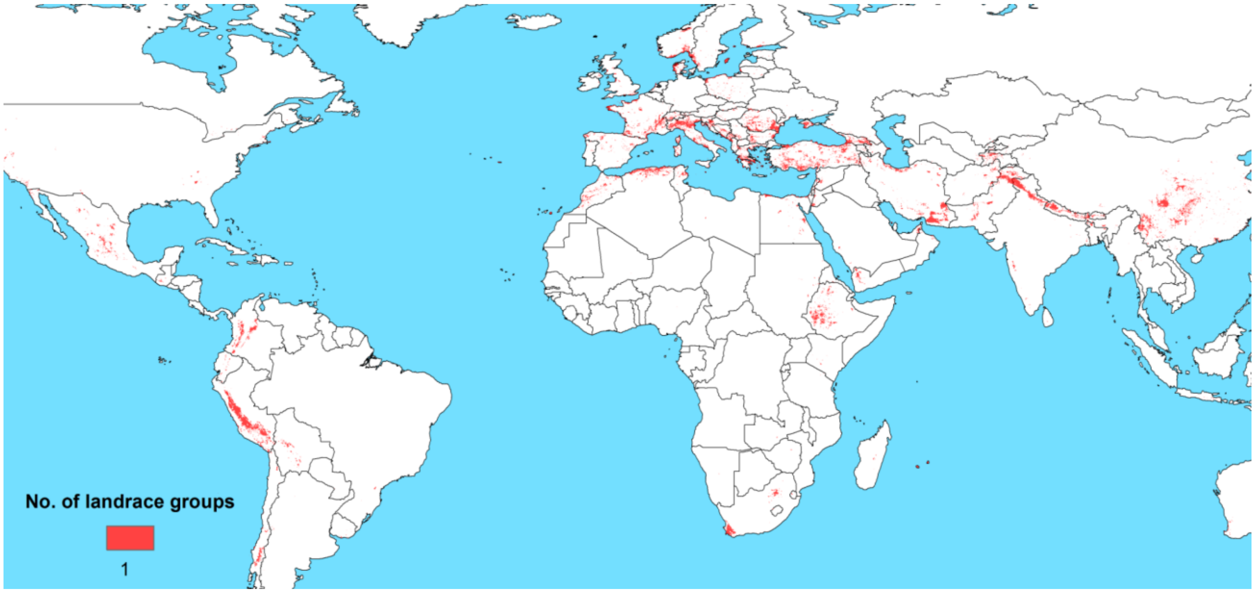


**Supplementary Fig. 13d**: Geographic gaps in the *ex situ* conservation of faba bean landraces.

*Grasspea*

Grasspea (*Lathyrus sativus* L.) is cultivated in the Mediterranean Basin and in temperate, as well as tropical, countries in Africa and West, South, and Central Asia. It's domestication likely began in the Balkan Peninsula during the Near Eastern agricultural expansion into the region.

Various studies of agro-morphological traits in grasspea have been conducted aimed at identifying and evaluating the level of genetic diversity among and within grasspea landraces around the world [87-91]. However, these classifications were very rarely attached to available occurrence data. We therefore performed the modeling and conservation gap analysis for the pulse at the crop level.

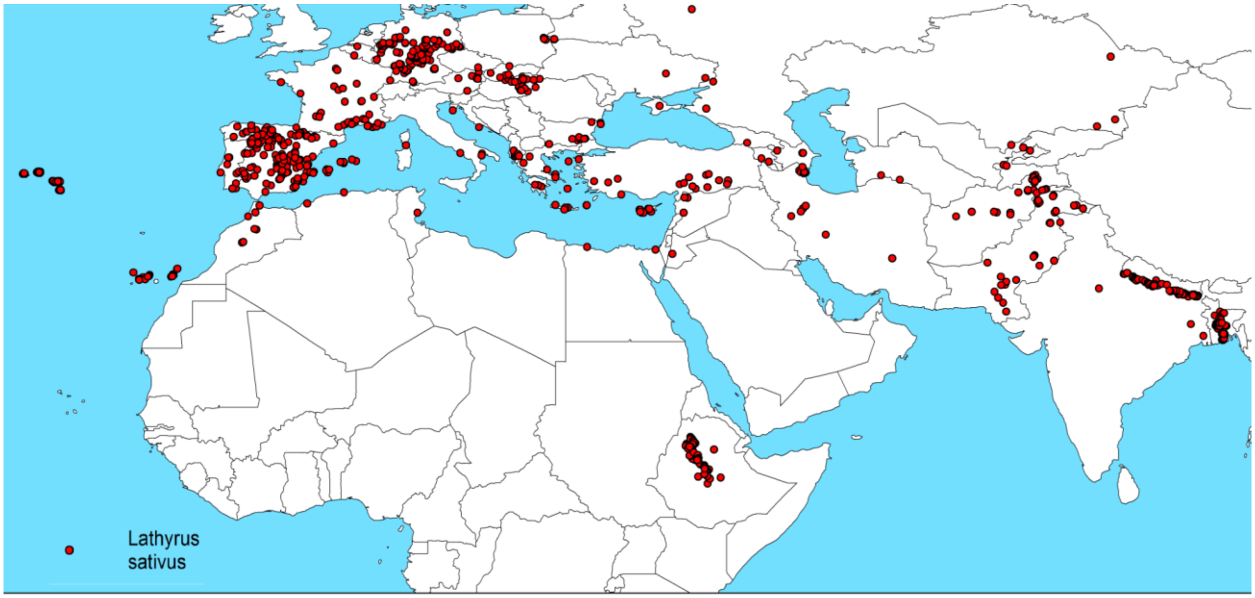


**Supplementary Fig. 14a:** All occurrences within the study region of grasspea landraces.

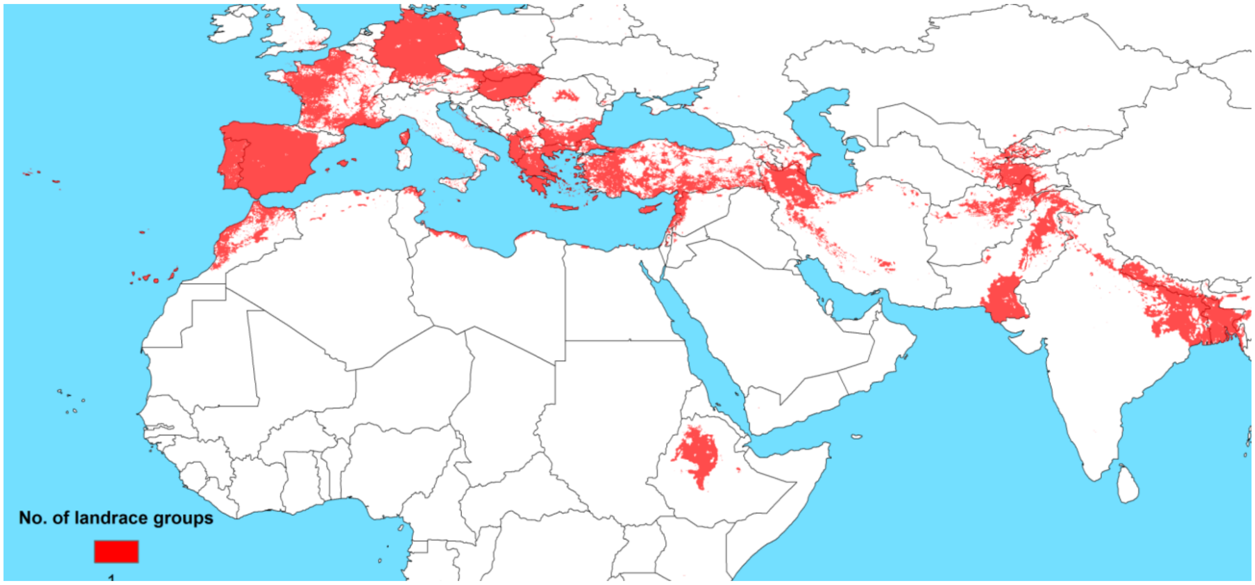


**Supplementary Fig. 14b**: Predicted distributions of grasspea landraces.

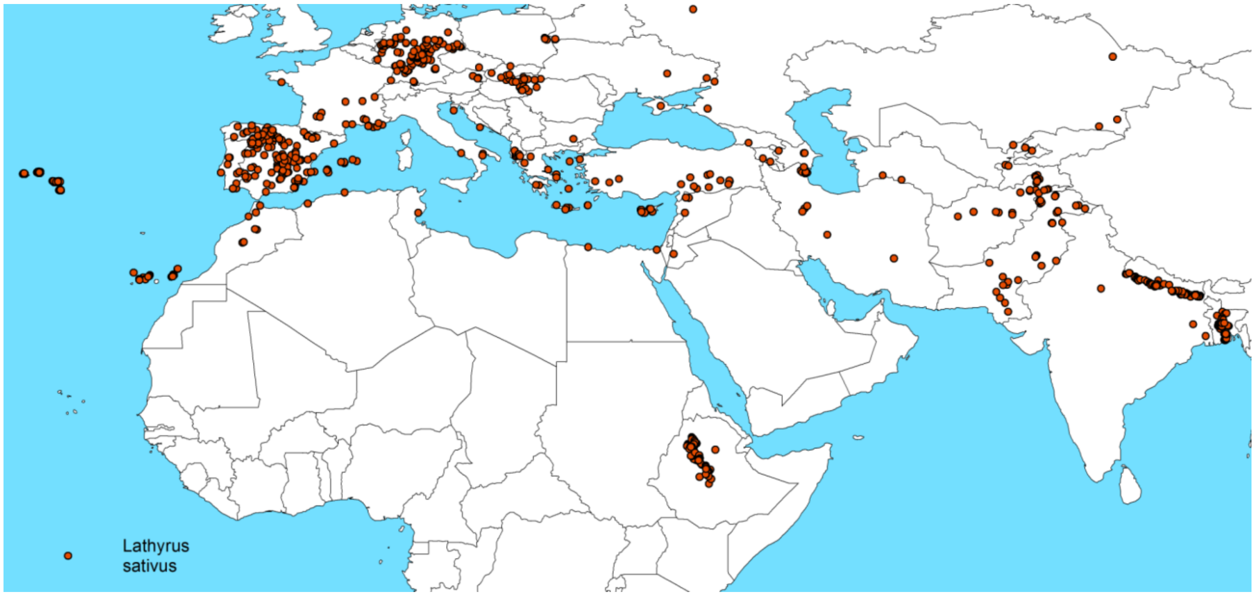


**Supplementary Fig. 14c**: Existing *ex situ* collection occurrences of grasspea landraces.

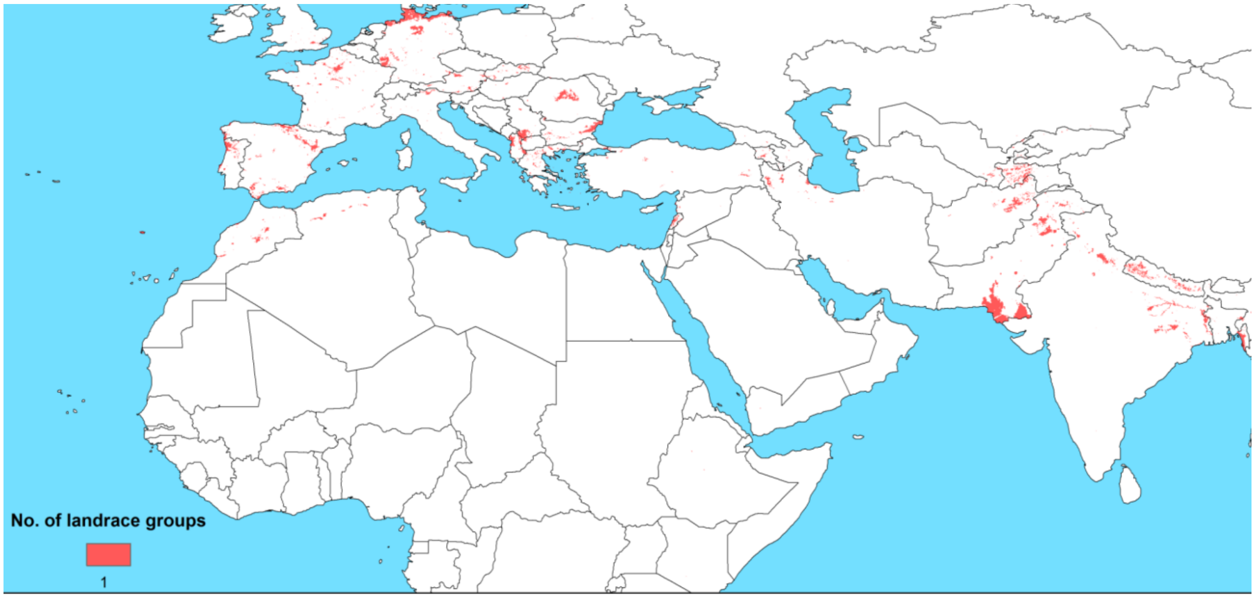


**Supplementary Fig. 14d**: Geographic gaps in the *ex situ* conservation of grasspea landraces.

*Groundnut*

Krapovickas and Gregory (1994) [92] in their classification divided groundnuts (*Arachis hypogaea* L.) into two subspecies: subsp. *hypogaea* and subsp. *fastigiata* Waldron. At the same time, they propose two and four botanical varieties within *hypogaea* and *fastigiata* Waldron, respectively.

*Arachis hypogaea* subsp. *hypogaea* is divided into varieties. *Hypogaea* is the predominant type and has a center of diversity in the vicinity of northwest Mato-Grosso in Brazil and in Bolivian and Amazonian geographic regions [93]. It is now also widely distributed in Africa and Asia. *Hirsuta* Köhler is another variety with a center of diversity in Peru, and has now spread across the Pacific to the Philippines, China, and India [94].

*Arachis hypogaea* subsp. *fastigiata* varieties are *fastigiata*, *vulgaris* C. Harz, *peruviana* Krapov. & W.C. Gregory, and *aequatoriana* Krapov. & W.C. Gregory. These varieties are distributed mainly across South America, but only *fastigiata* and *vulgaris* are widely cultivated, including in the Americas, Africa, and Asia.

However, in the data we gathered there were not enough occurrences of each variety within the subspecies; for instance, *peruviana*, *hirsuta*, and *vulgaris* each had fewer than 70 occurrences, and most of the occurrences came from *fastigiata* and *hypogaea* varieties. For this reason, we performed our analysis at the subspecies level across the distribution range of cultivated groundnuts. The machine learning models reached an accuracy of 76.1%: 85.67% for *fastigiata* and 61.79% for *hypogaea*.

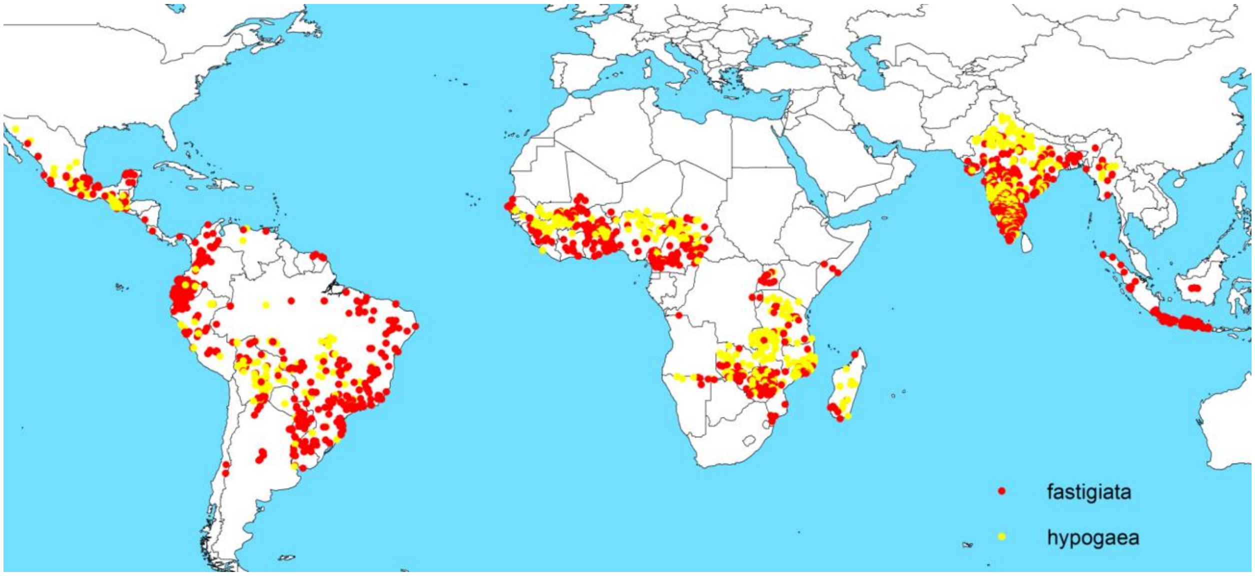


**Supplementary Fig. 15a**: All occurrences within the study region of groundnut landrace groups.

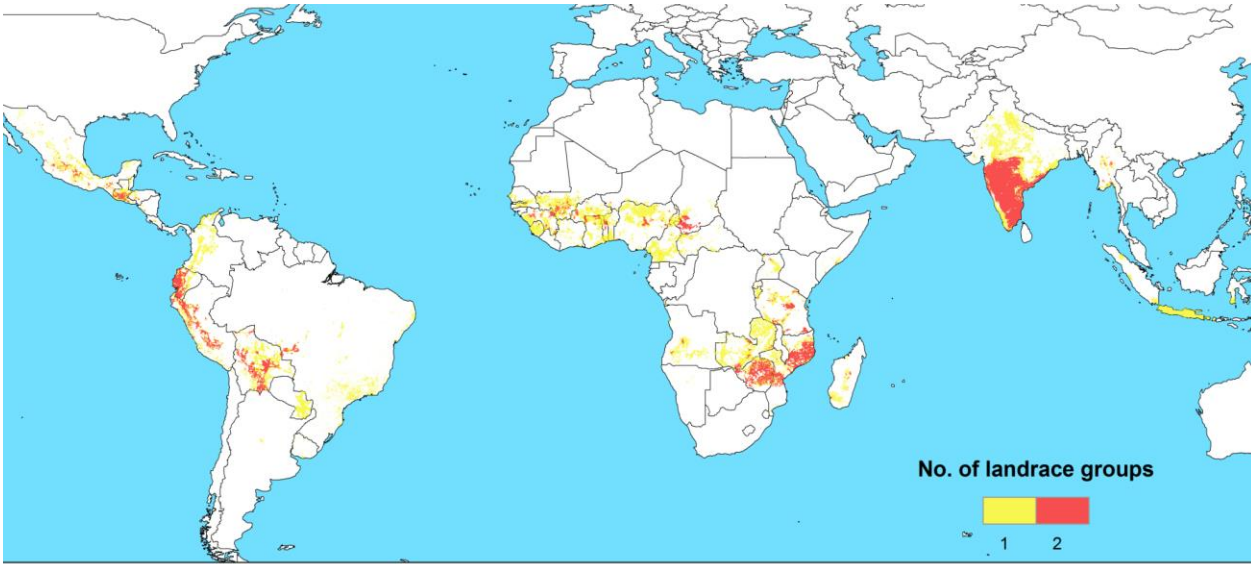


**Supplementary Fig. 15b**: Predicted distributions of groundnut landrace groups.

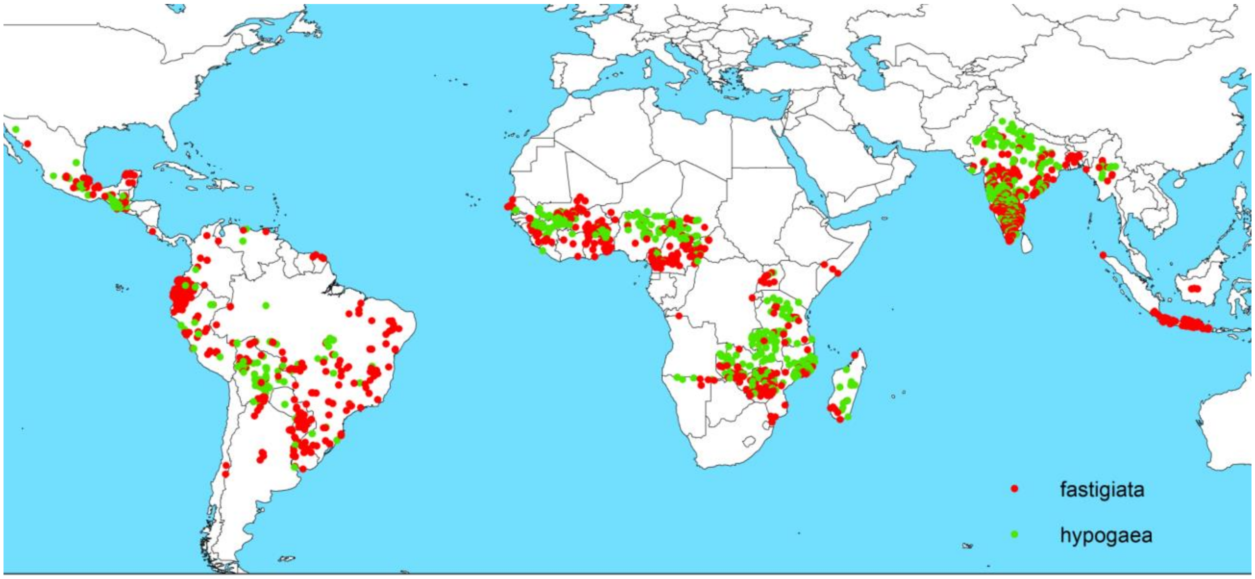


**Supplementary Fig. 15c**: Existing *ex situ* collection occurrences of groundnut landrace groups.

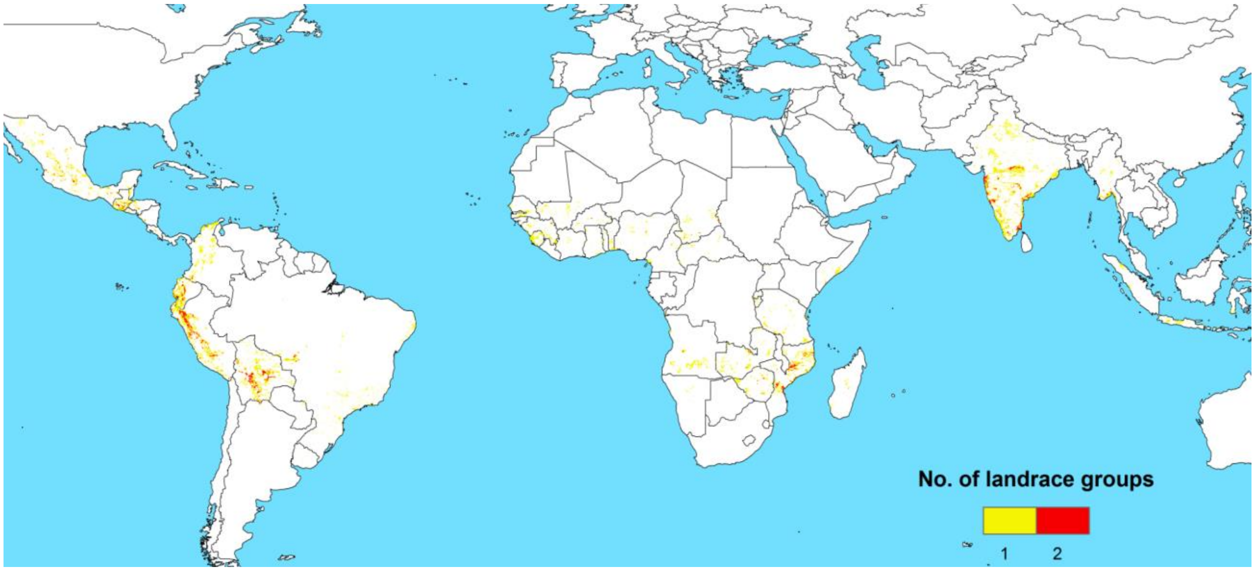


**Supplementary Fig. 15d**: Geographic gaps in the *ex situ* conservation of groundnut landrace groups.

*Lentil*

Our literature review indicated that *Lens culinaris* Medik. subsp. *culinaris* is the only cultivated species of lentils. Barulina (1930) [95] subdivided cultivated lentils into two types - *microsperma* and *macrosperma* - based on seed characteristics. *Macrosperma* is characterized by large seeds and yellow cotyledons, while *microsperma* has small seeds and orange-red or yellow cotyledons.

To complement this information, we used bioclimatic and socioeconomic variables to perform a cluster analysis in order to understand how the occurrences may be grouped by environment. Two main groupings emerged. The variables that contributed the most to construct the groups were as follows:

1. The maximum temperature in the coldest quarter.
2. The mean temperature of the coldest quarter.
3. The minimum temperature of the coldest month.
4. The thermicity index.
5. The number of months with a mean temperature greater than 10ºC.

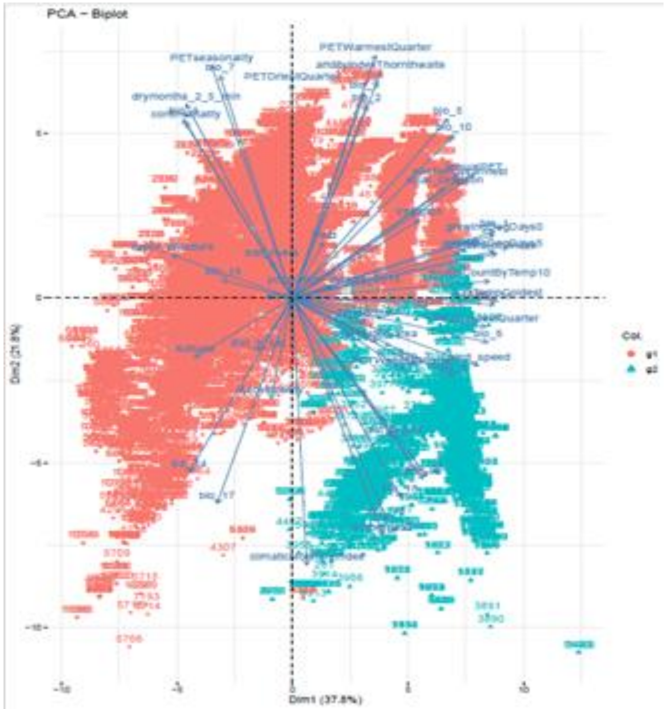


As the biplot shows, occurrences belonging to group 1 are closer to major cities, and they grow in areas with the following characteristics:

- The precipitation of the coldest quarter is more than double the precipitation in the areas where group 2 grows, and the mean temperature in the coldest quarter is lower;
- During the coldest months, the minimum temperature reaches below 0ºC and the maximum temperature is lower than in areas where group 2 grows;
- The maximum consecutive number of dry months with temperatures greater than 10ºC is larger, and during those months, the standard deviation and annual range of temperatures are higher than in areas where group 2 grows; and
- The intensity of the winter cold, known as the thermicity index, is lower than in areas where group 2 grows.

Occurrences belonging to group 2 grow in areas where the following conditions apply:

- The production and harvested areas are bigger than in areas where group 1 grows;
- The intensity of the winter cold, known as the thermicity index, is higher;
- The distance from these areas to areas where the primary genepool (crop wild relative) occurs is longer than for group 1;
- The crop is closer to larger human populations;
- The mean monthly potential evapotranspiration (PET) of the wettest quarter is more than double the value in areas where group 1 grows;
- The mean monthly PET of the coldest quarter is larger;
- The maximum temperature in the coldest quarter is higher; and
- The emberger's pluviothermic quotient is larger.

Cluster analyses based on seed weight and cotyledon color from the ICARDA dataset, largely aligned with the environmental clustering, although the environmental analysis produced stronger accuracy. The occurrences in our final environmental group 1 have mostly yellow cotyledon color and larger seeds, whereas occurrences in group 2 have mostly red or orange cotyledon color and smaller seeds.

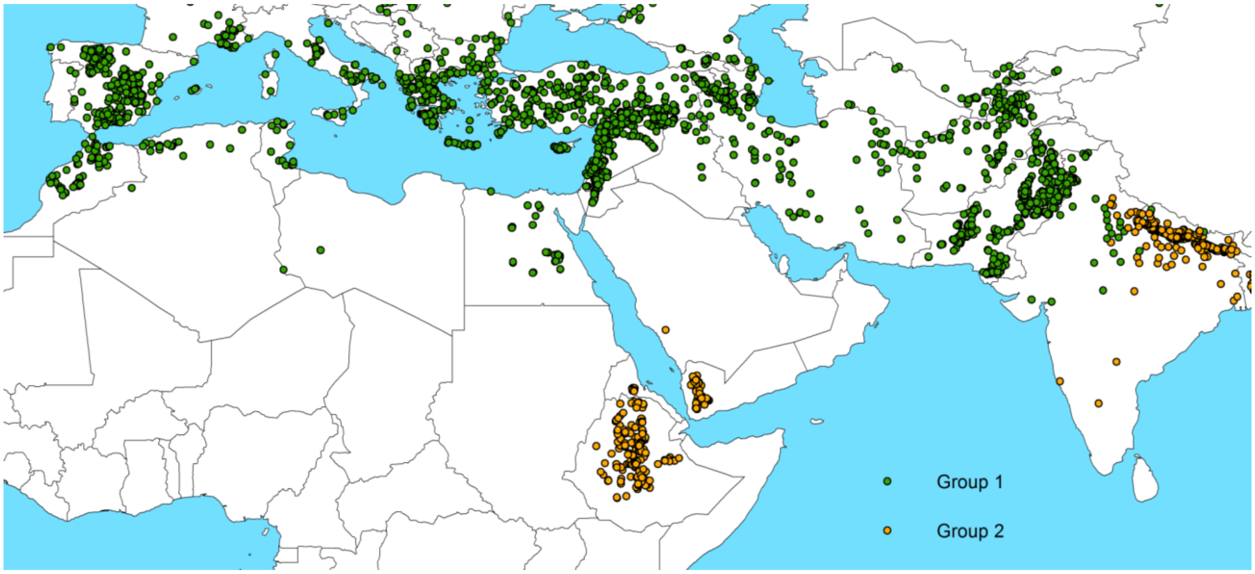

**Supplementary Fig. 16a**: All occurrences within the study region of lentil landrace groups.

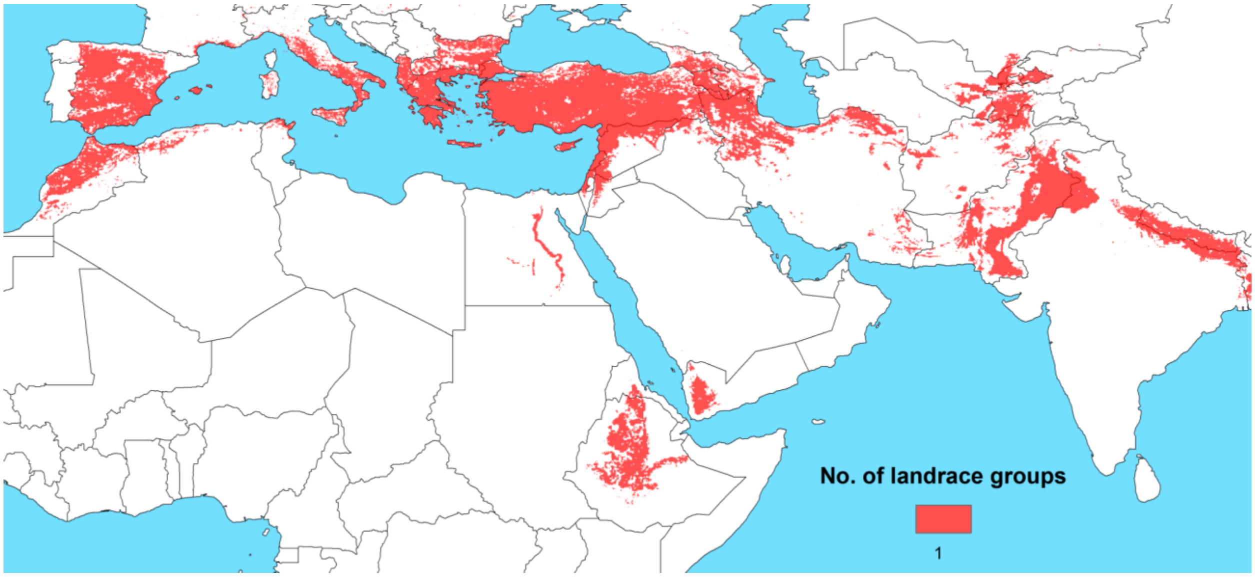

**Supplementary Fig. 16b**: Predicted distributions of lentil landrace groups.

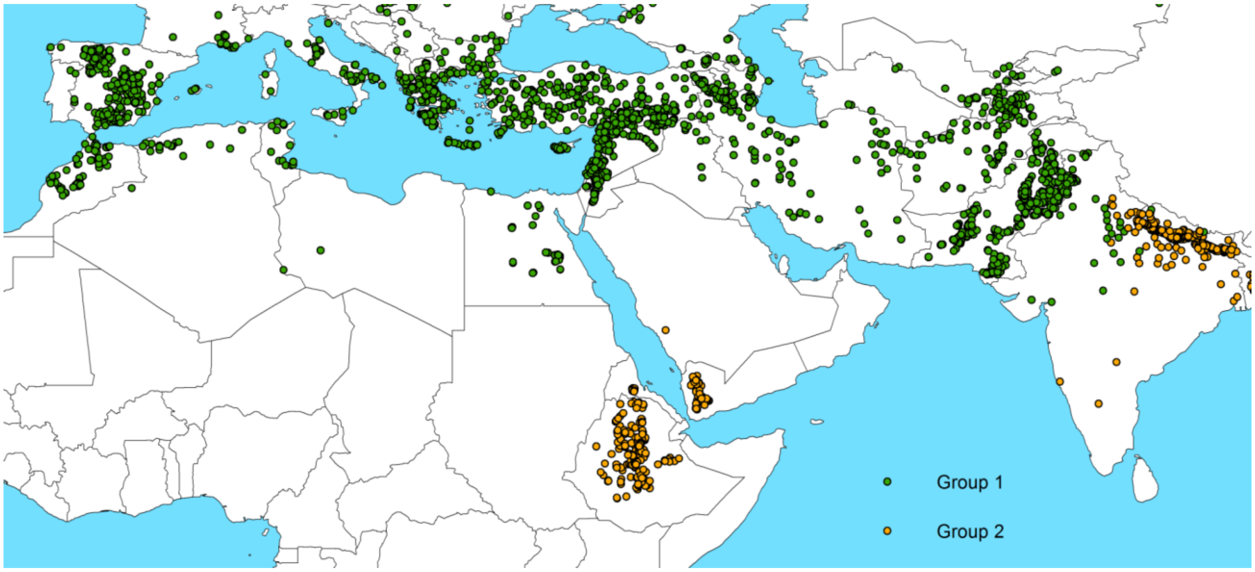


**Supplementary Fig. 16c**: Existing *ex situ* collection occurrences of lentil landrace groups.

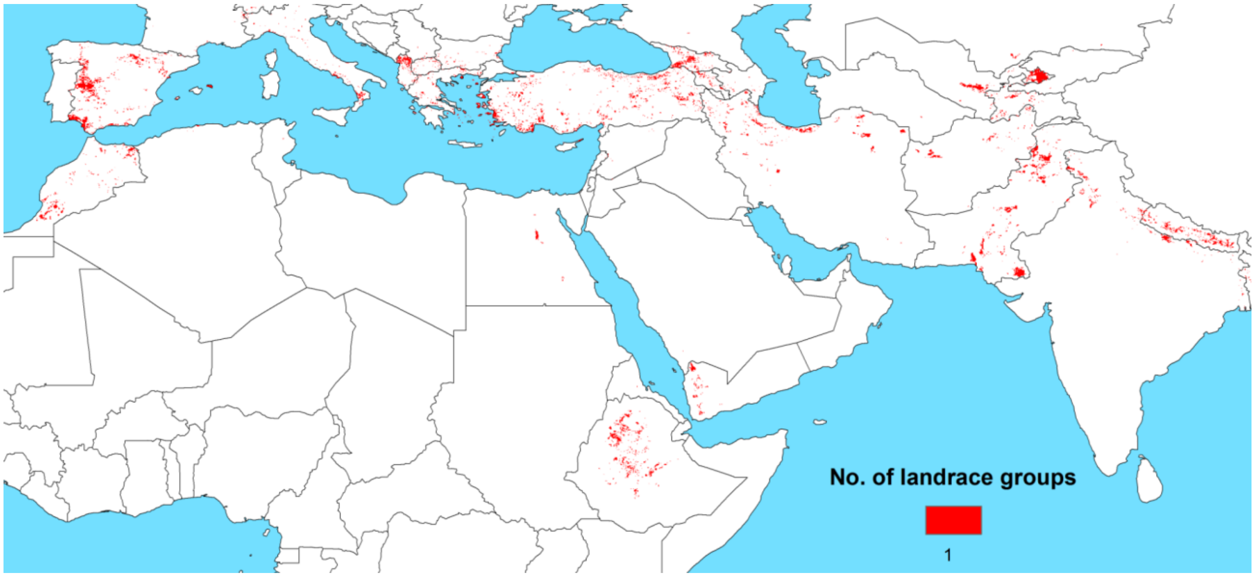


**Supplementary Fig. 16d**: Geographic gaps in the *ex situ* conservation of lentil landrace groups.

*Pea*

The genus *Pisum* L. includes the wild species *Pisum fulvum* Sm. and the wild/cultivated species *Pisum abyssinicum* A. Braun and *Pisum sativum* L. The crops are distributed throughout the world, while the native types are found in Europe and northwest Asia, extending south into temperate East Africa [96]. Two major forms of cultivated peas are distinguishable: field pea, *Pisum sativum* var. *arvense* (L.) Poir, and garden pea, *Pisum sativum* var. *sativum* L.. The first is commonly used for livestock fodder and green manure, while the second is the major variety for human consumption. We performed the modelling and conservation gap analysis based on the *arvense* and *sativum* subspecies classifications. The machine learning models reached an accuracy of 87.1%: 94.6% for *arvense,* and 81.7% for *sativum*.

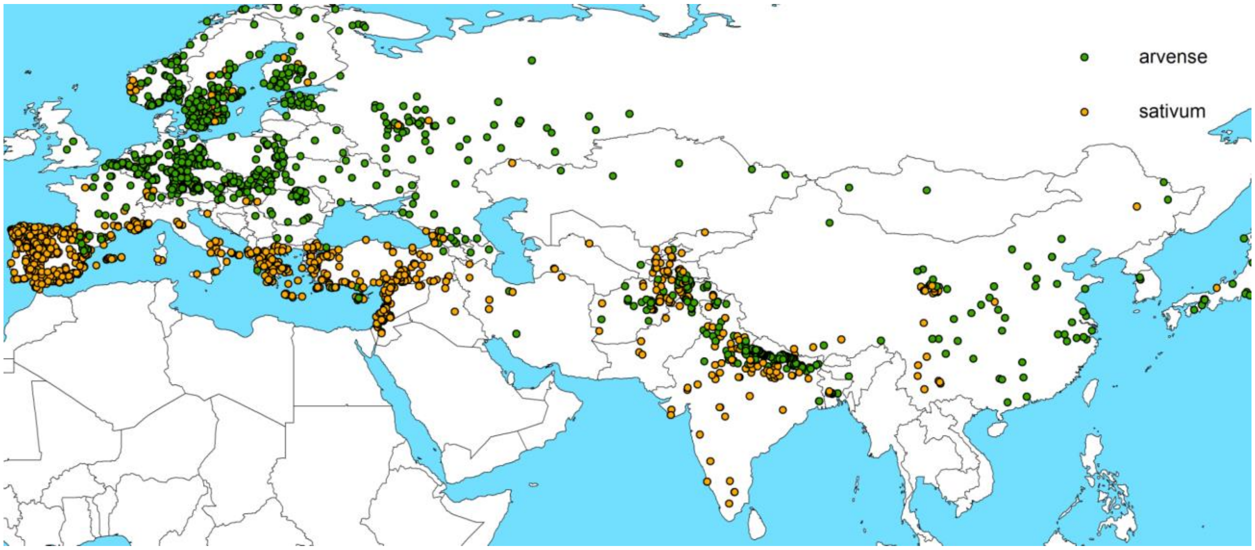


**Supplementary Fig. 17a**: All occurrences within the study region of pea landrace groups.

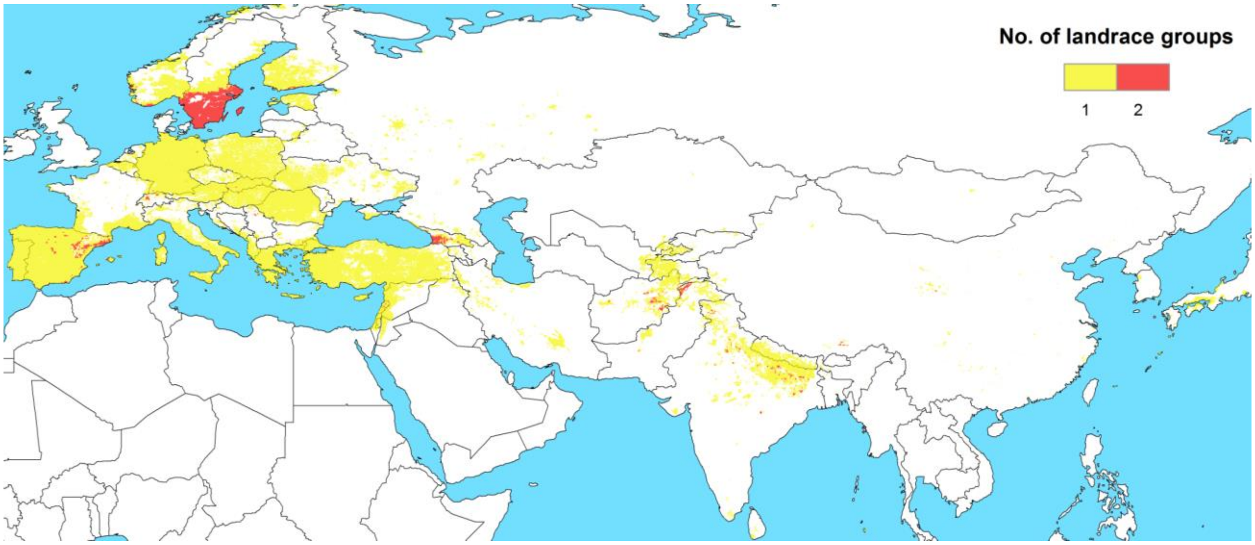


**Supplementary Fig. 17b**: Predicted distributions  of pea landrace groups.

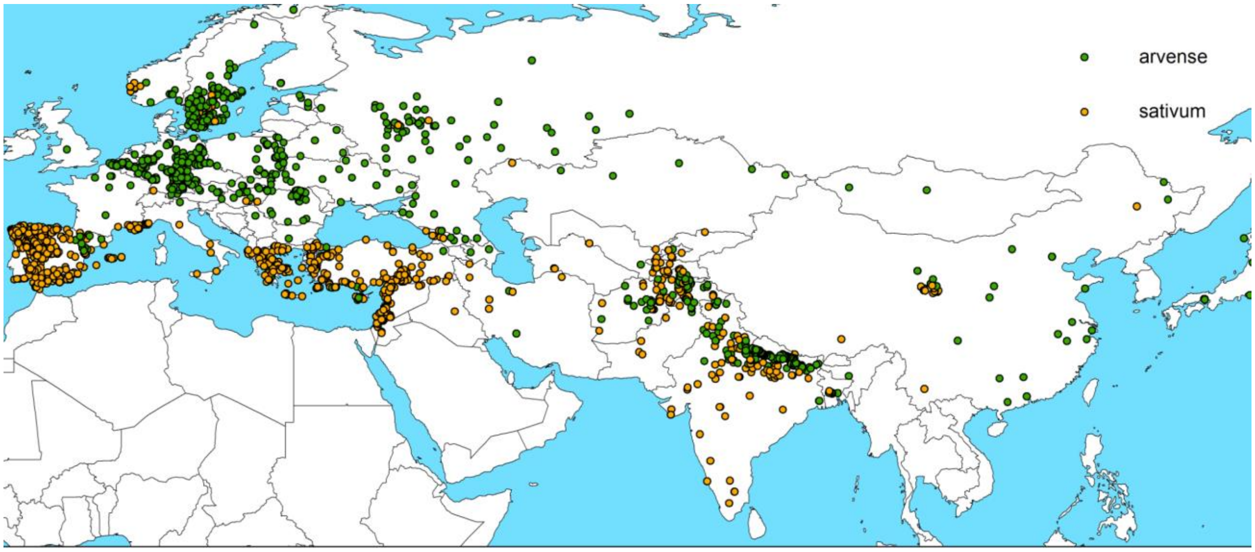


**Supplementary Fig. 17c**: Existing *ex situ* collection occurrences of pea landrace groups.

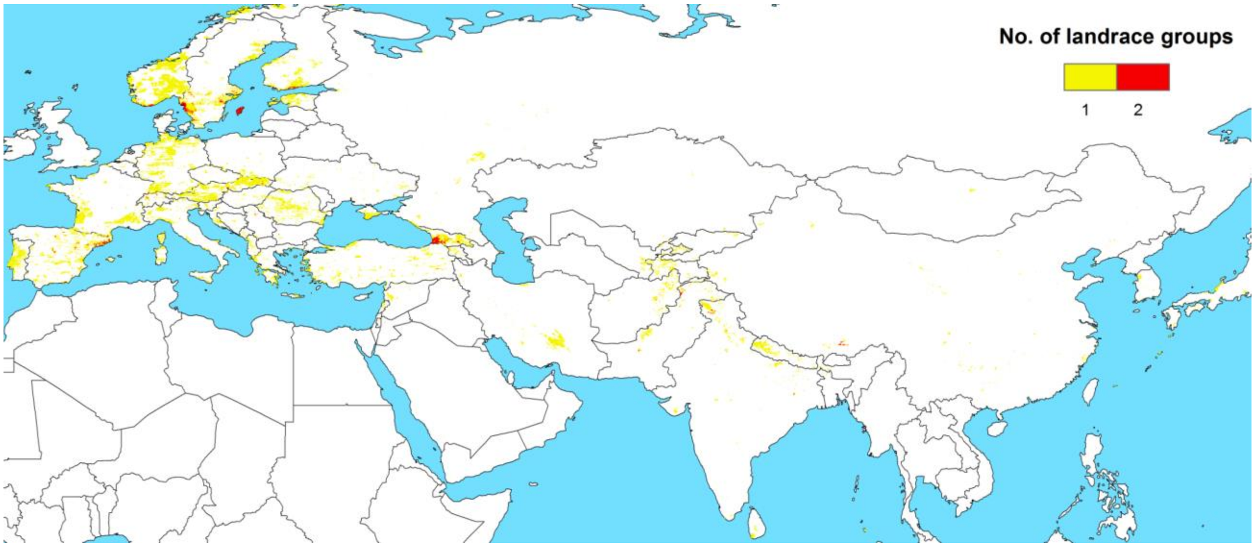


**Supplementary Fig. 17d**: Geographic gaps in the *ex situ* conservation of pea landrace groups.

*Pigeonpea*

Pigeonpea, *Cajanus cajan* (L.) Millsp., is a grain legume crop grown in several tropical and subtropical countries for human food and a variety of other purposes. It is widely adapted and drought-tolerant, with a large temporal variation for maturity, and can therefore be cultivated in a broad range of agroecological settings [97]. There is no established infraspecific structure for the crop and scarce data attached to available accessions indicating major differences. Based on our literature review, we decided to perform the modeling and conservation gap analysis for the entire crop in the semi-arid tropics of Asia and Africa.

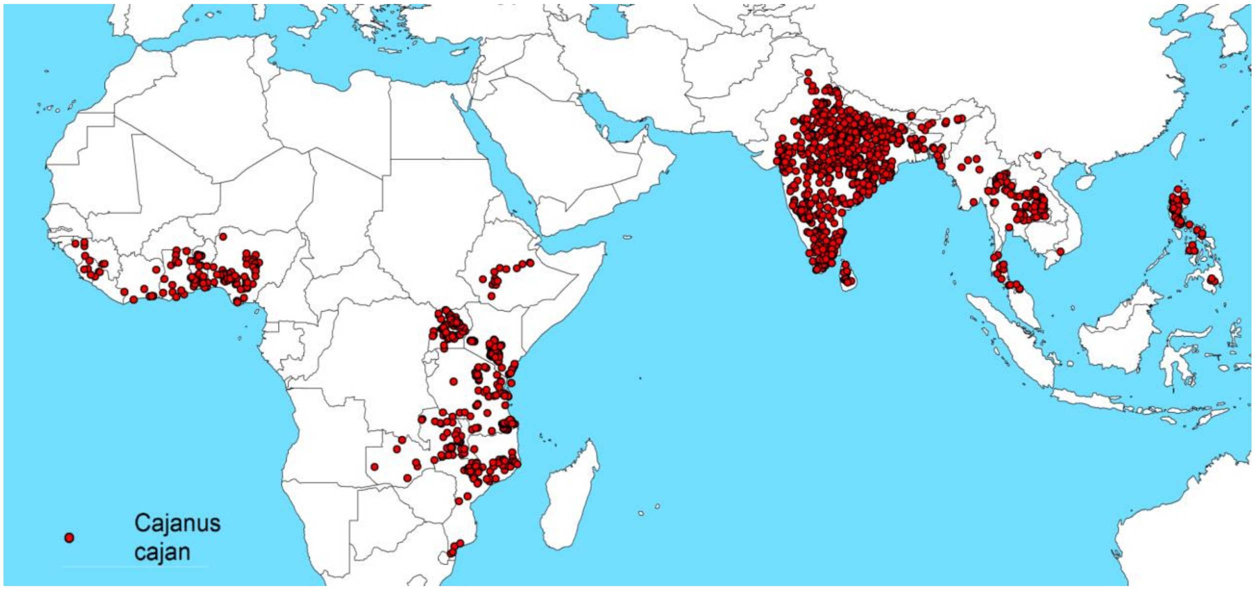


**Supplementary Fig. 18a**: All occurrences within the study region of pigeonpea landraces.

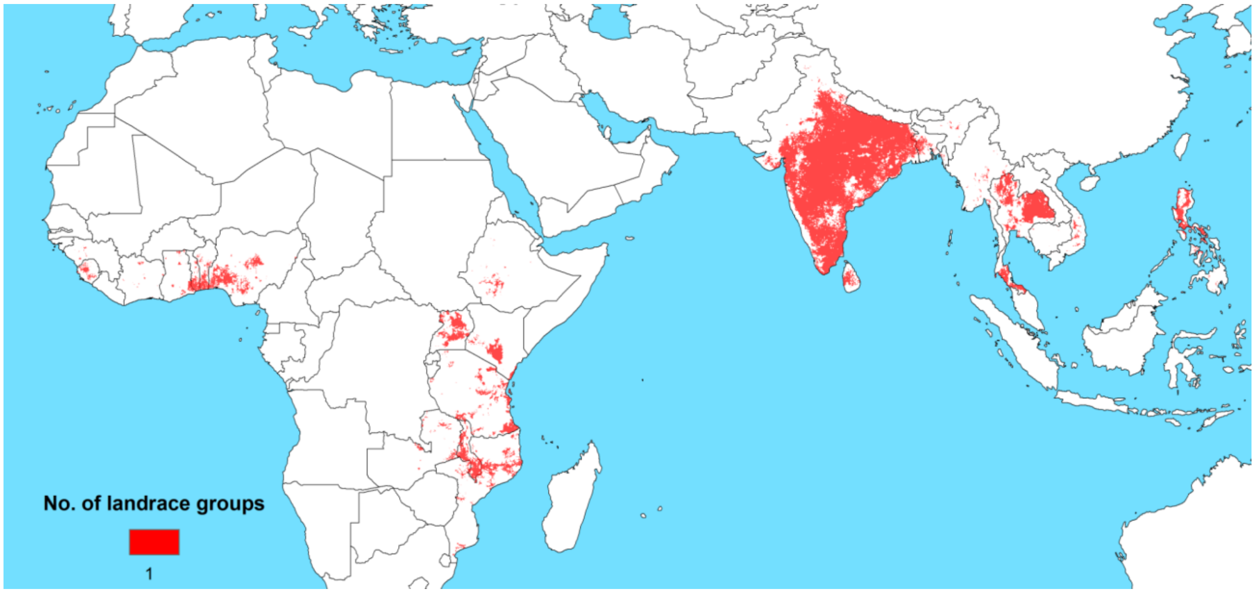


**Supplementary Fig. 18b**: Predicted distributions of pigeonpea landraces.

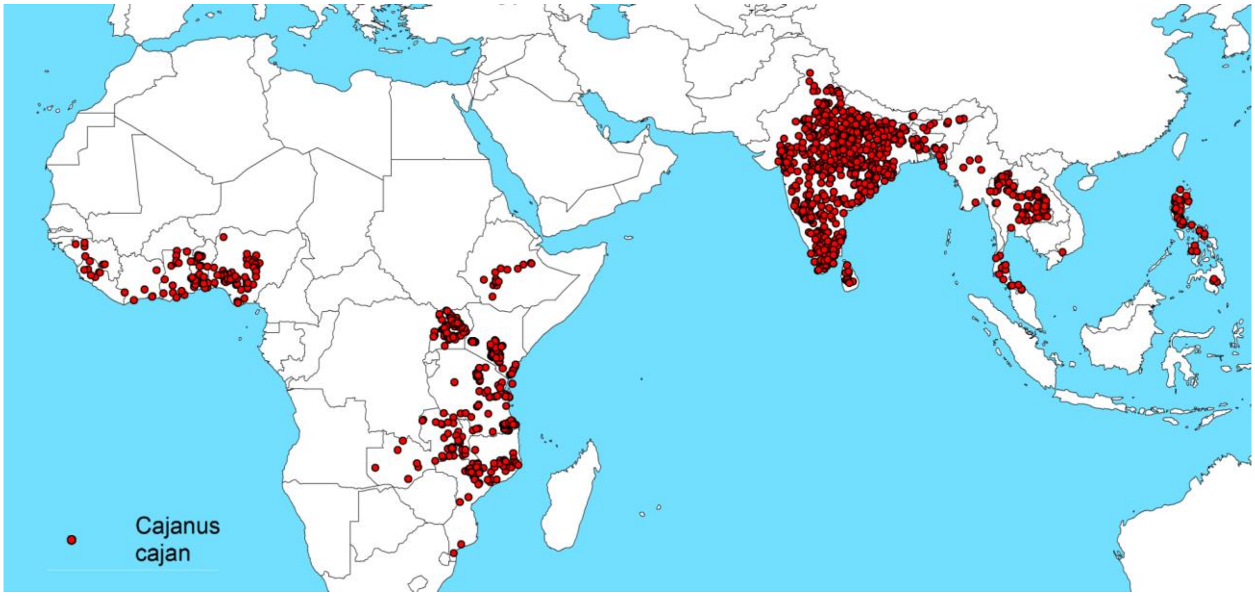

**Supplementary Fig. 18c**: Existing *ex situ* collection occurrences of pigeonpea landraces.

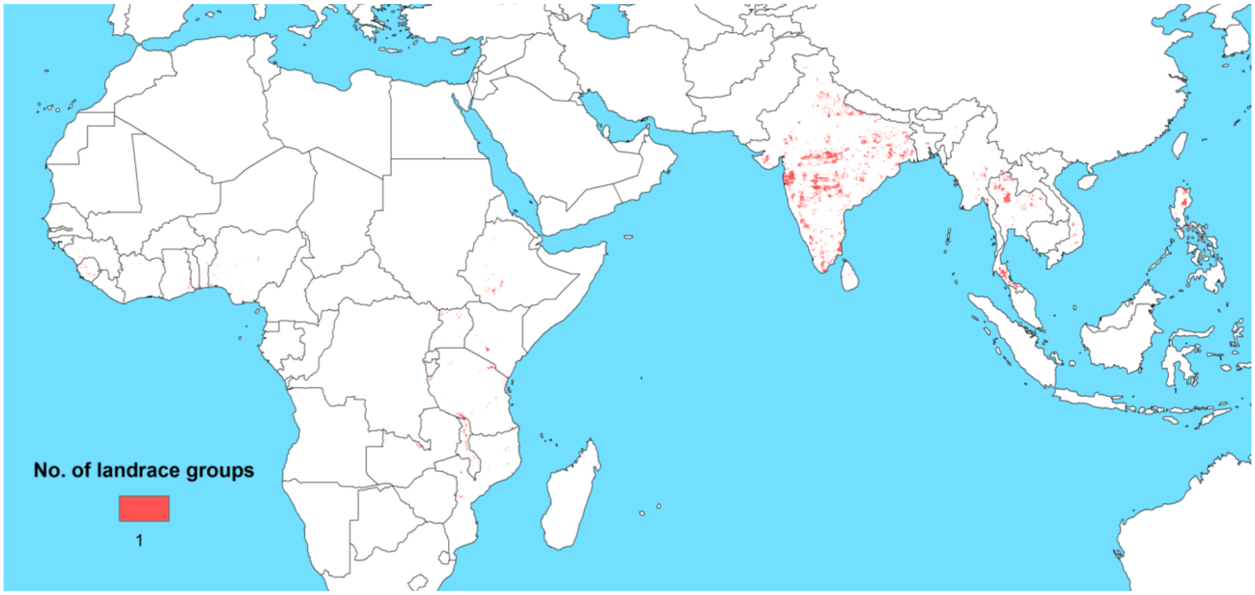

**Supplementary Fig. 18d**: Geographic gaps in the *ex situ* conservation of pigeonpea landraces.

*Starchy roots, tubers, and fruits*

*Banana and plantain*

Our literature review indicated that multiple domestications and subsequent introgressions from wild types of *Musa acuminata* Colla and *Musa balbisiana* Colla may have occurred in Southeast Asia and the Pacific [98]. Most edible bananas are diploids or triploids from *M. acuminata* (A-genome: AA or AAA) alone or from hybridization with *M. balbisiana* (B-genome: AAB or ABB), although there are a wide variety of edible types with different ploidy levels and genomes: AA, AB, AAA, AAB, ABB, AAT, AAS, AABB, AAAB, ABBB, ABBT [99]. A separate group, including Fe'i bananas, is confined to the Pacific region and is derived from *Australimusa* species [99,100]. Vegetative propagation of major diploid and triploid varieties over long periods has led to somaclonal variants with substantial phenotypic diversity (Perrier et al. 2011). Cultivated bananas have been documented in Southeast Asia for at least 7,000 years, and in West Africa for at least 3,000 years [99].

Many names are associated with banana and plantain landraces, and a smaller number of sets with common origins and distinctive but overlapping distributions have been described [99]. These groups appear to have been cultivated for very long periods in their respective areas. Phylogenetics, on the other hand, have not clearly distinguished groups beyond the A and B genome differentiations [98].

Through our literature review and available data, we identified the following possible landrace group classifiers: ploidy levels of 2, 3, or 4; genome groups A, B, S, T, FEI, and others; and vernacular names. The use of names, however, was not pragmatic because there are very many distinct local names for the same landrace type [100]. Our attempts to classify groups based on the association of ploidy or genome with environment produced low accuracy, perhaps in part due to the many forms of the crop being grown widely in the tropics. We therefore performed the modeling and conservation gap analysis for the starchy fruit at the crop level.

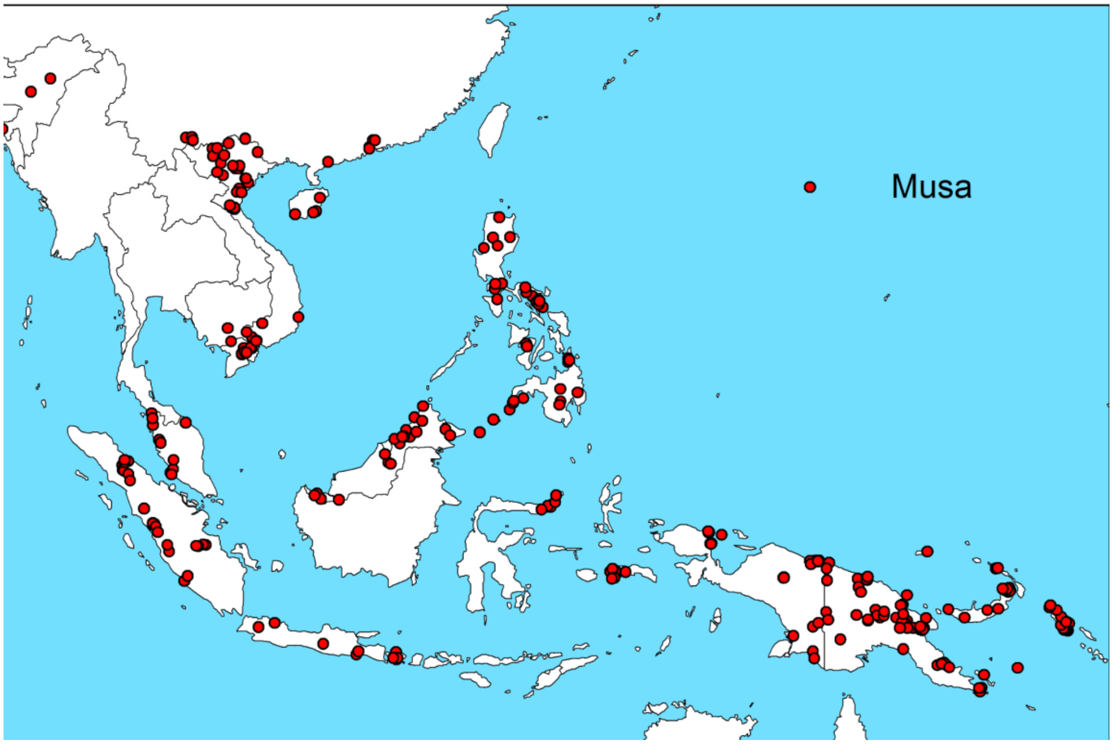


**Supplementary Fig. 19a**: All occurrences within the study region of banana and plantain landraces.

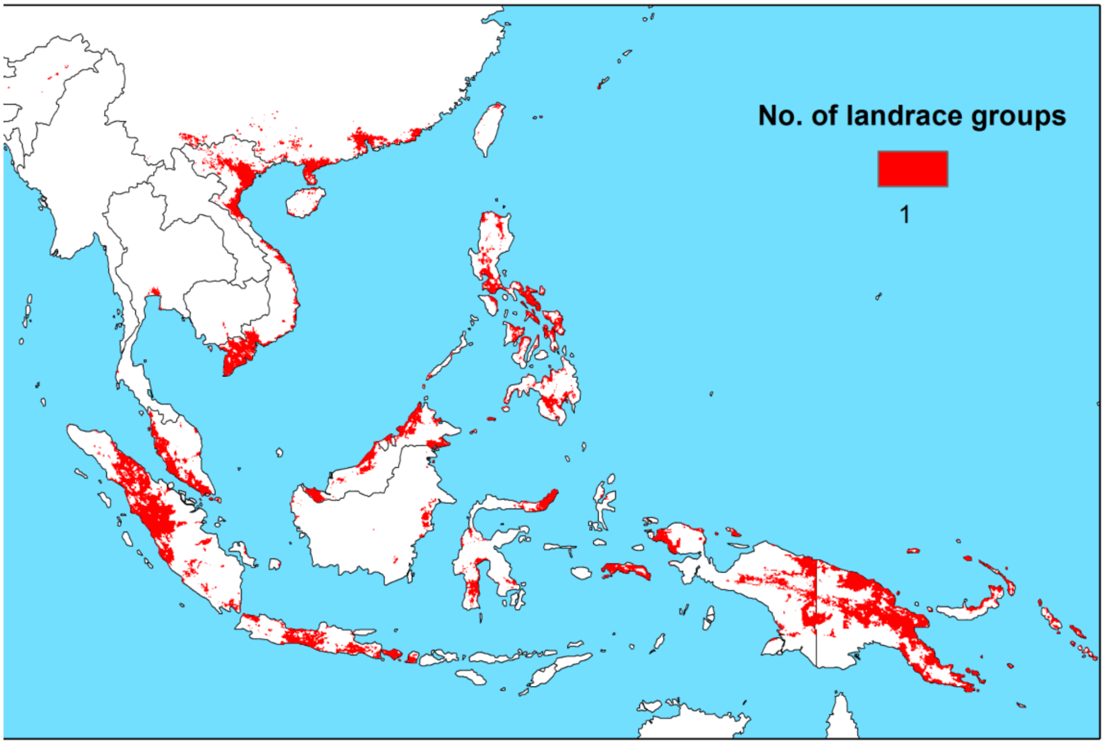


**Supplementary Fig. 19b**: Predicted distributions of banana and plantain landraces.

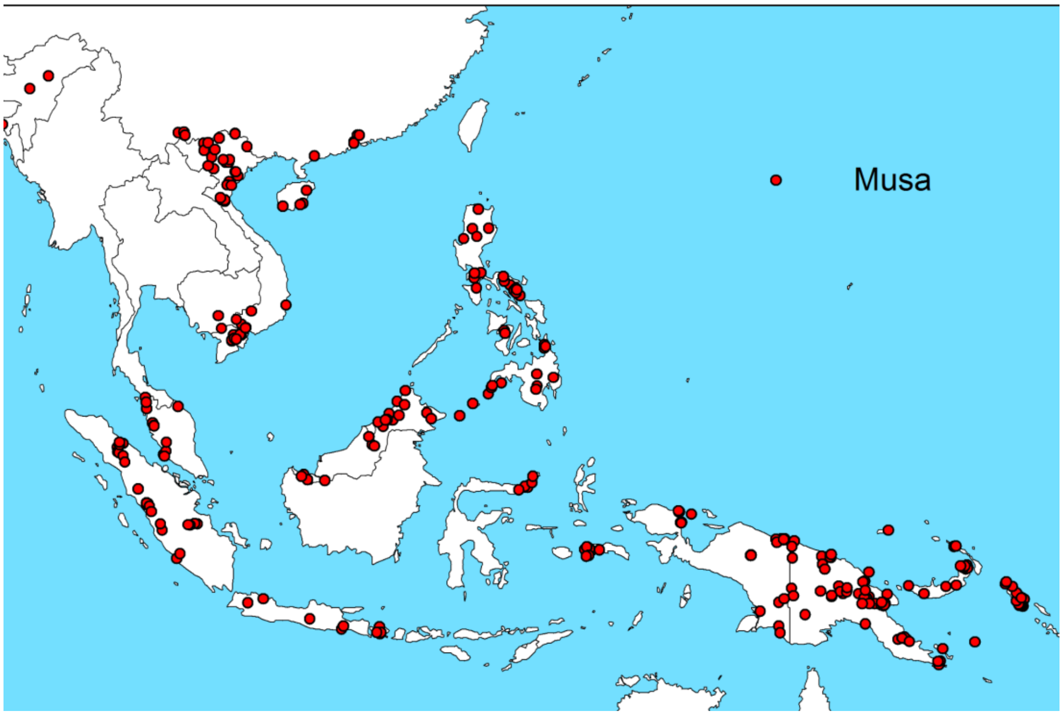


**Supplementary Fig. 19c**: Existing *ex situ* collection occurrences of banana and plantain landraces.

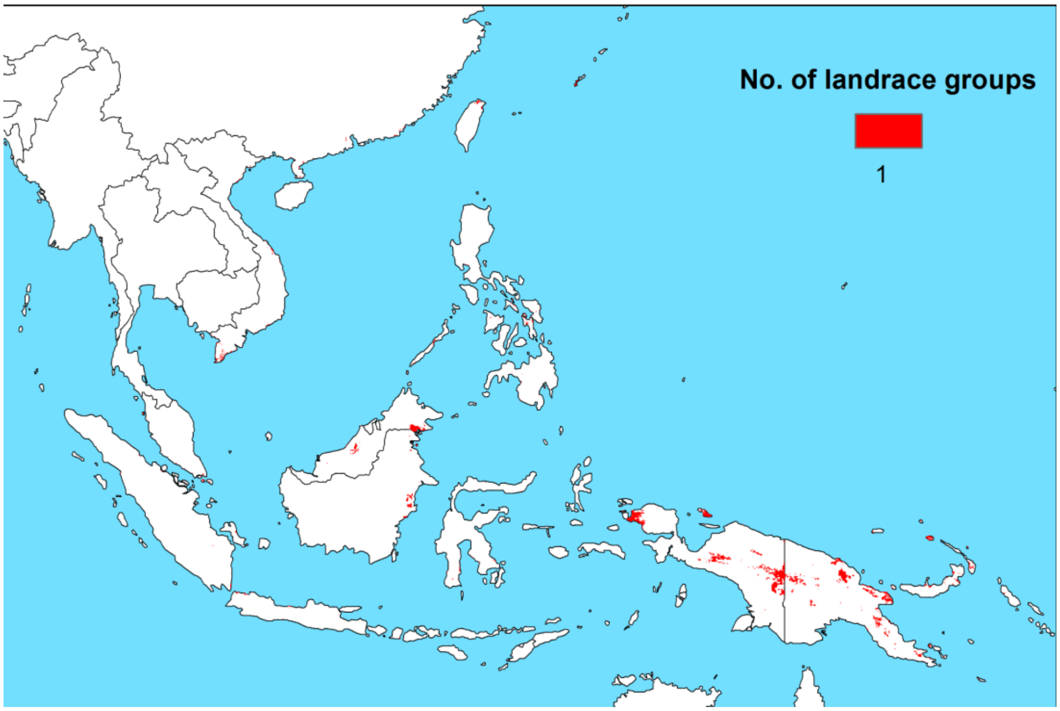


**Supplementary Fig. 19d**: Geographic gaps in the *ex situ* conservation of banana and plantain landraces.

*Breadfruit*

Breadfruit (*Artocarpus altilis* (Parkinson) Fosberg) is a tree producing a large starchy fruit which is a staple food of the South Pacific, Southeast Asia, and various other tropical areas. The genetic diversity and importance of breadfruit are considered to be most significant in Oceania, perhaps with particular emphasis on Melanesia, where breadfruit was likely domesticated [101-103].

Various efforts have been made to characterize, evaluate, and describe the germplasm of this crop with high morphological variability. Multiple species of cultivated breadfruit have been described, including *Artocarpus camansi* Blanco and *Artocarpus mariannensis* Trécul, alongside *altilis*. All of them occur in the Pacific region and show a wide range of morphological characteristics, from true seedless fruits to fruits with numerous, minute, aborted seeds, to fruits with one or a few viable seeds, to fruits with numerous seeds. Many authors group all of this variability within one species [104], and the most broadly accepted name for breadfruit is the single species *Artocarpus altilis* [105,106], which has taxonomic priority. Based on this literature review and the limited characterization data attached to available occurrences, we performed the modeling and conservation gap analysis for the starchy fruit at the crop level, in the Pacific through Southeast Asia.

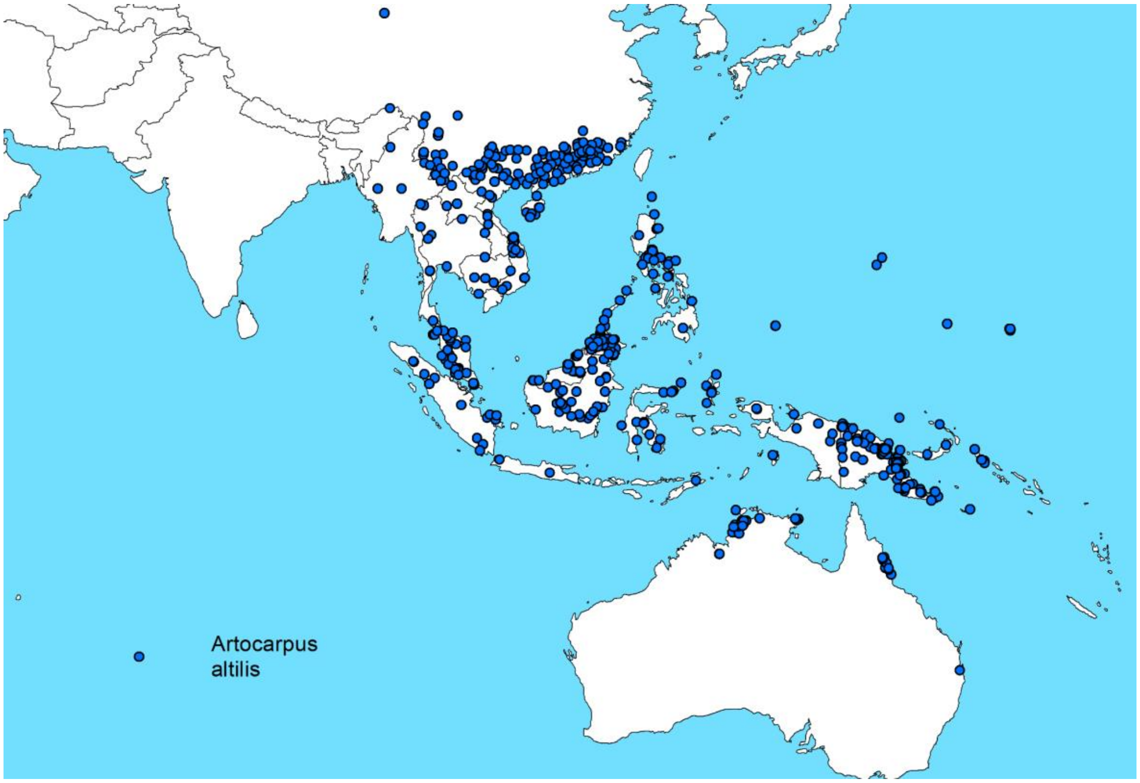


**Supplementary Fig. 20a:** All occurrences within the study region of breadfruit landraces.

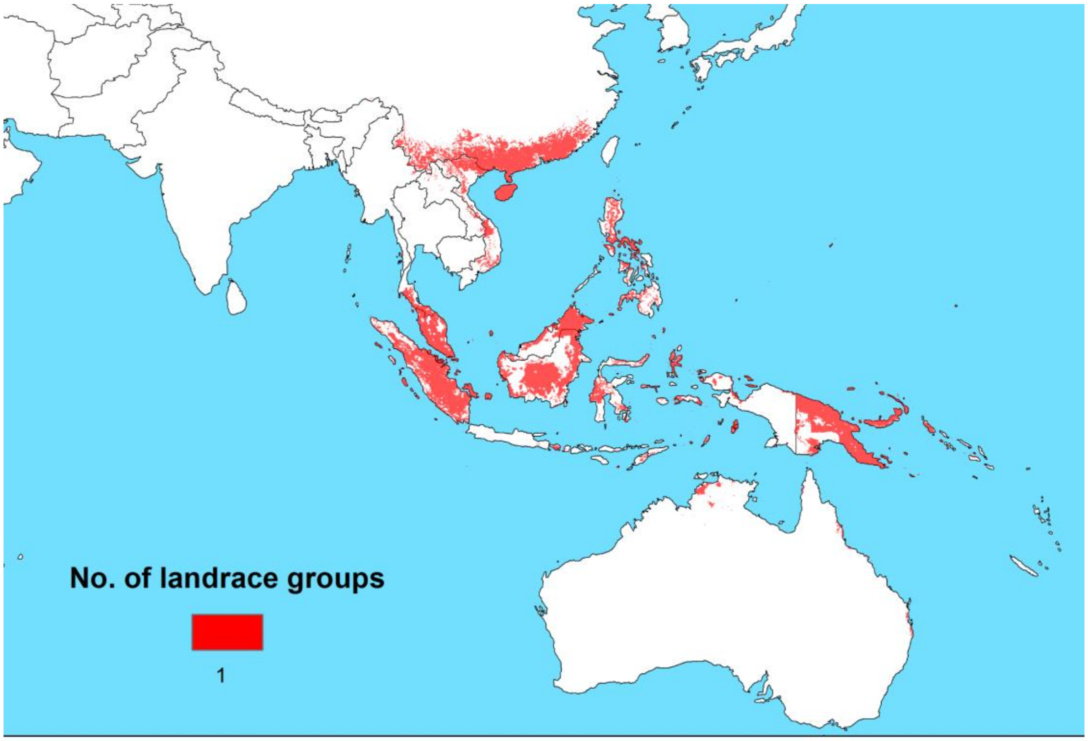


**Supplementary Fig. 20b**: Predicted distributions of breadfruit landraces.

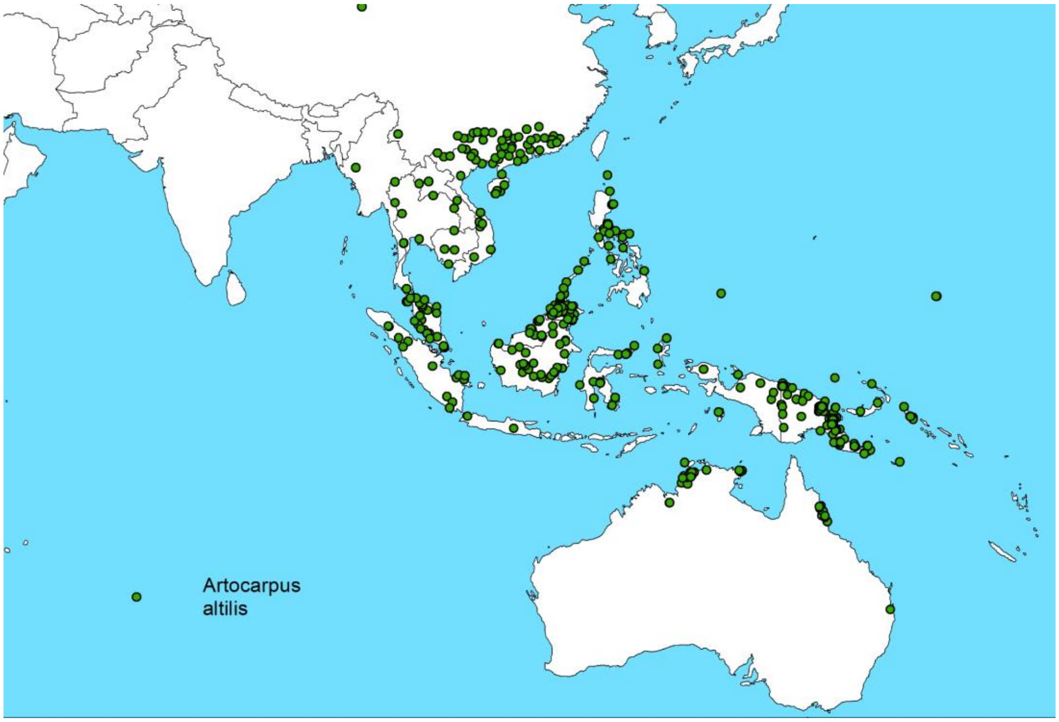


**Supplementary Fig. 20c**: Existing *ex situ* collection occurrences of breadfruit landraces.

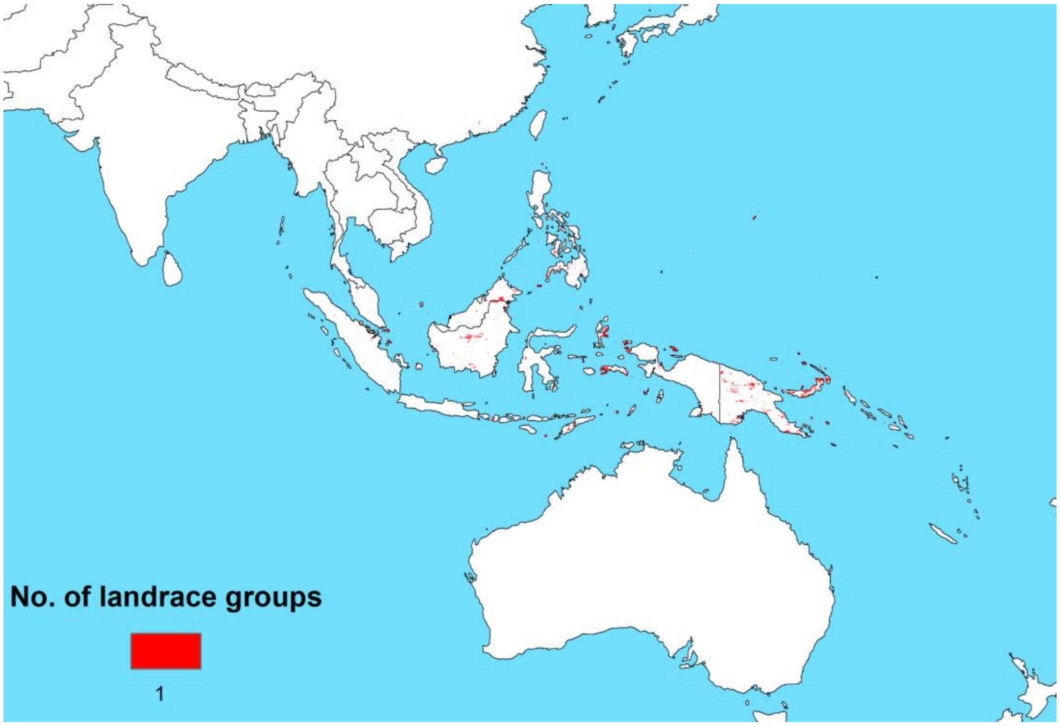


**Supplementary Fig. 20d**: Geographic gaps in the *ex situ* conservation of breadfruit landraces.

*Cassava*

*Manihot esculenta* Crantz subsp. *esculenta* is a staple starchy crop with great economic importance worldwide; however, its precise geographical origins remain unresolved and debatable [107]. Some evidence suggests that the domestication event occurred from the wild relative *Manihot esculenta* Crantz subsp. *flabellifolia* (Pohl) Cifs in neotropical lowland savannas and southern portions of the Amazon rainforest around 7000 BCE [108].

Some research has focused on genetically characterizing cassava, mainly concluding that this crop demonstrates high genetic variability and shows some ecogeographic patterns [109-111]. On the other hand, there is no strong agreement about infraspecific classifications and insufficient information and limited data attached to occurrences.

We implemented a cluster analysis using bioclimatic and socioeconomic variables to assess whether the crop could be classified by environment. A principal component analysis was carried out, plus a hierarchical clustering of components to obtain the optimal number of clusters. As a result, four environmental clusters were identified. The biplot figure depicts the grouping of the accessions and the influence upon them of the environmental and socioeconomic variables.

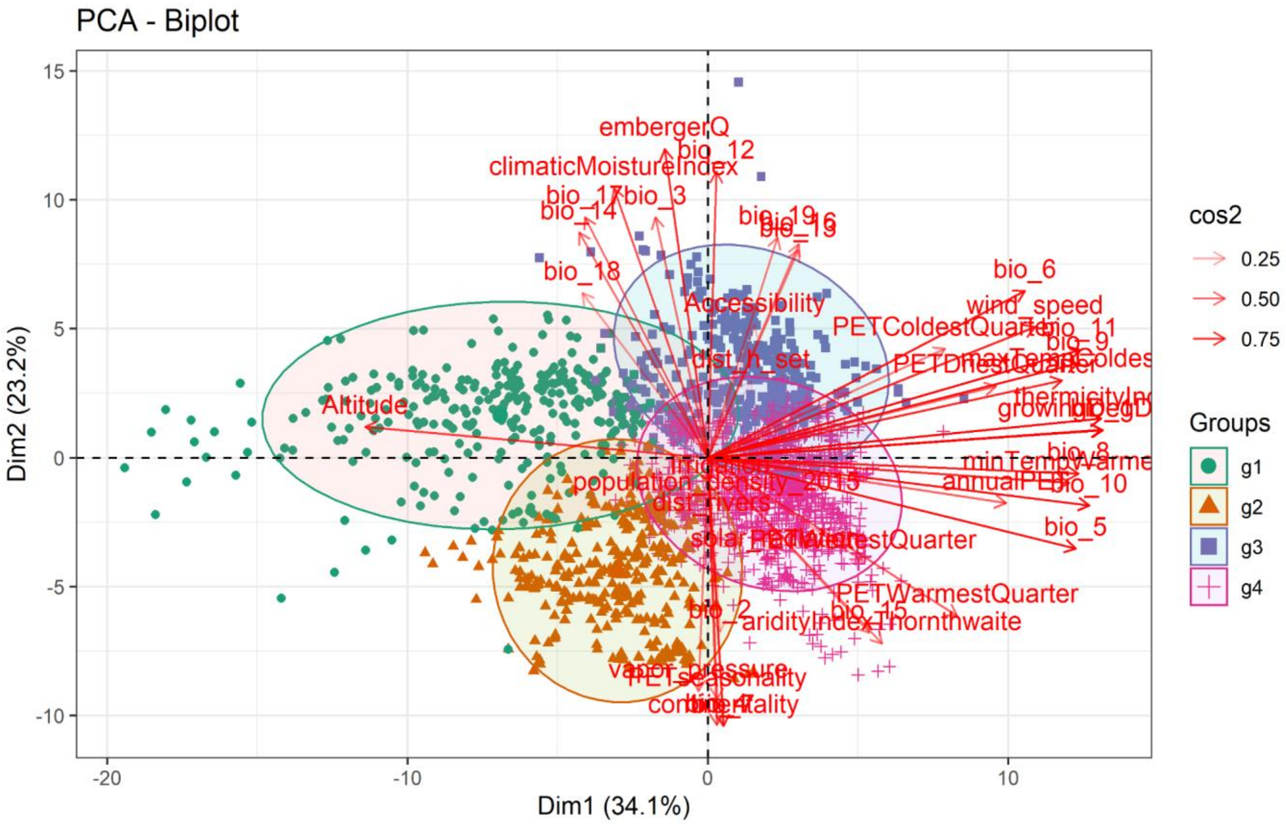

Group 1 is characterized by high altitudes, substantial irrigation, plentiful precipitation in the driest month, low-temperature seasonality, and low continentality. Group 2, in turn, is characterized by considerable irrigation, but less than group 1; high temperature seasonality; high continentality; strong seasonality of potential evapotranspiration (PET seasonality); a wide annual temperature range; scant precipitation during the coldest quarter; and a low minimum temperature during the coldest month. Group 3, meanwhile, is characterized by very substantial precipitation in the driest month, more even than group 1; substantial precipitation in the driest quarter; substantial precipitation in the coldest quarter; scant irrigation; low population density; short distances to rivers; low altitudes; and limited accessibility. Finally, group 4 is characterized by considerable population density, high temperature seasonality, long distances to rivers, high continentality, scant precipitation in the driest month, scant irrigation, and low precipitation in the driest quarter.

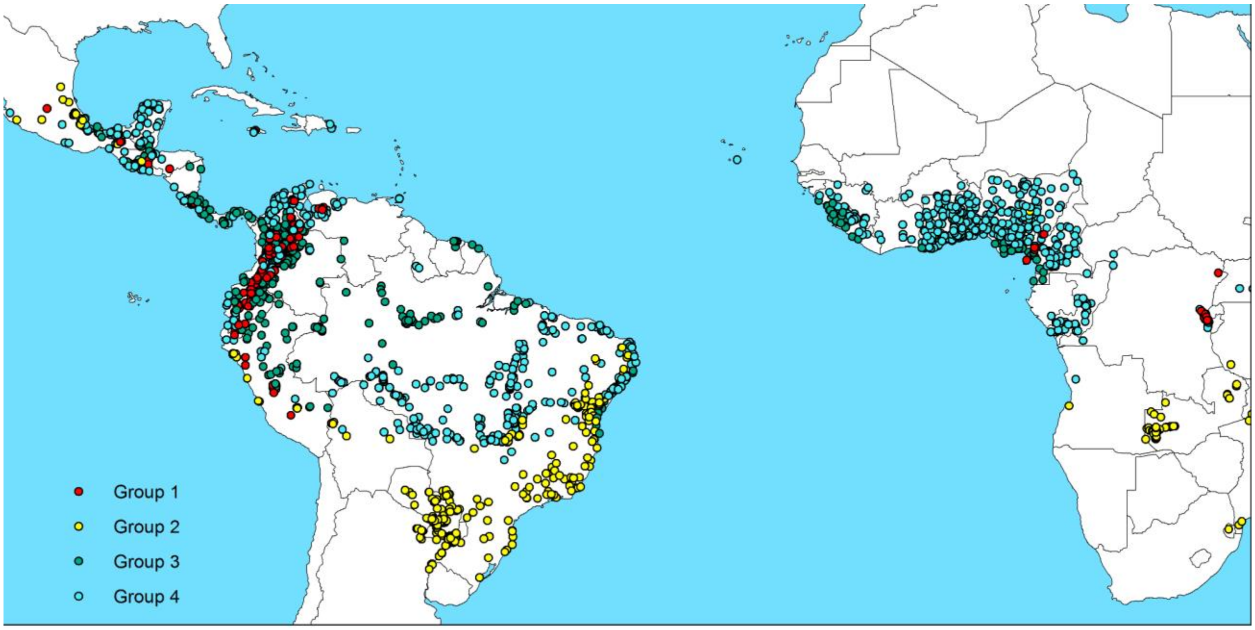


**Supplementary Fig. 21a**: All occurrences within the study region of cassava landrace groups.

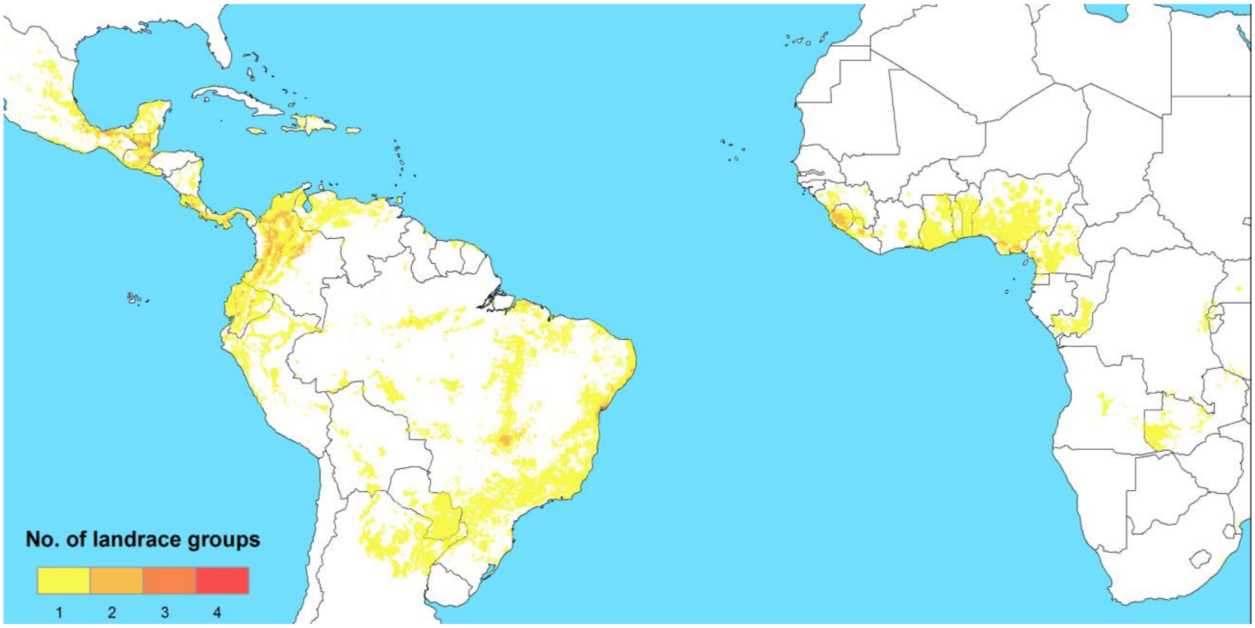


**Supplementary Fig. 21b**: Predicted distributions of cassava landrace groups.

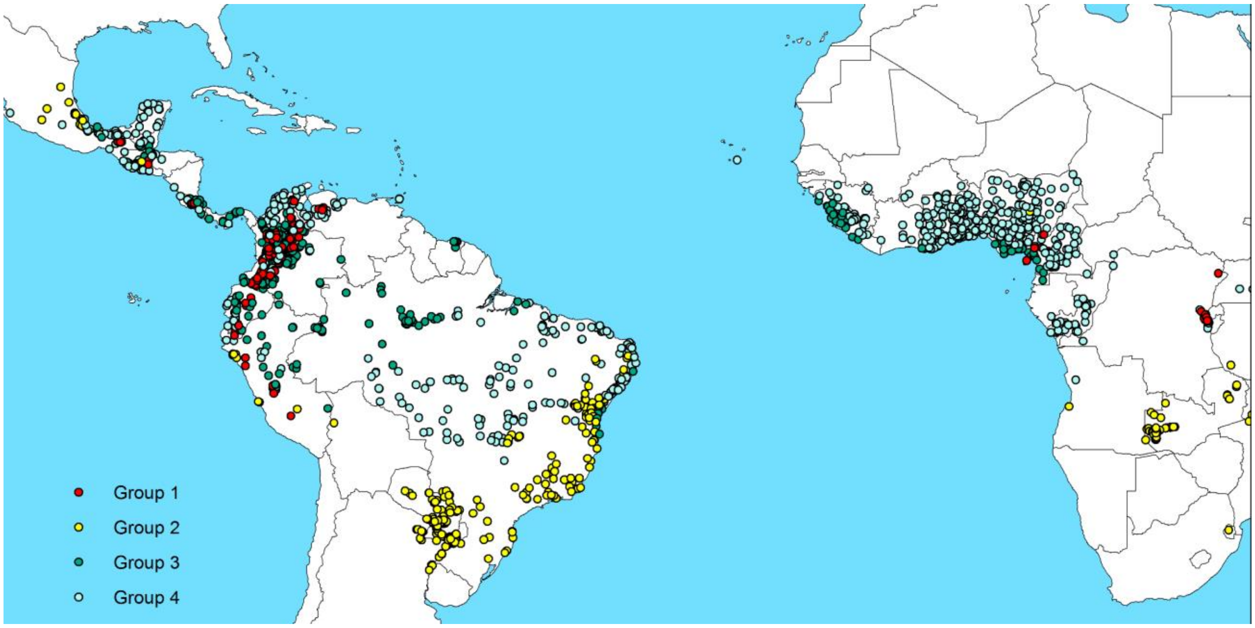


**Supplementary Fig. 21c**: Existing *ex situ* collection occurrences of cassava landrace groups.

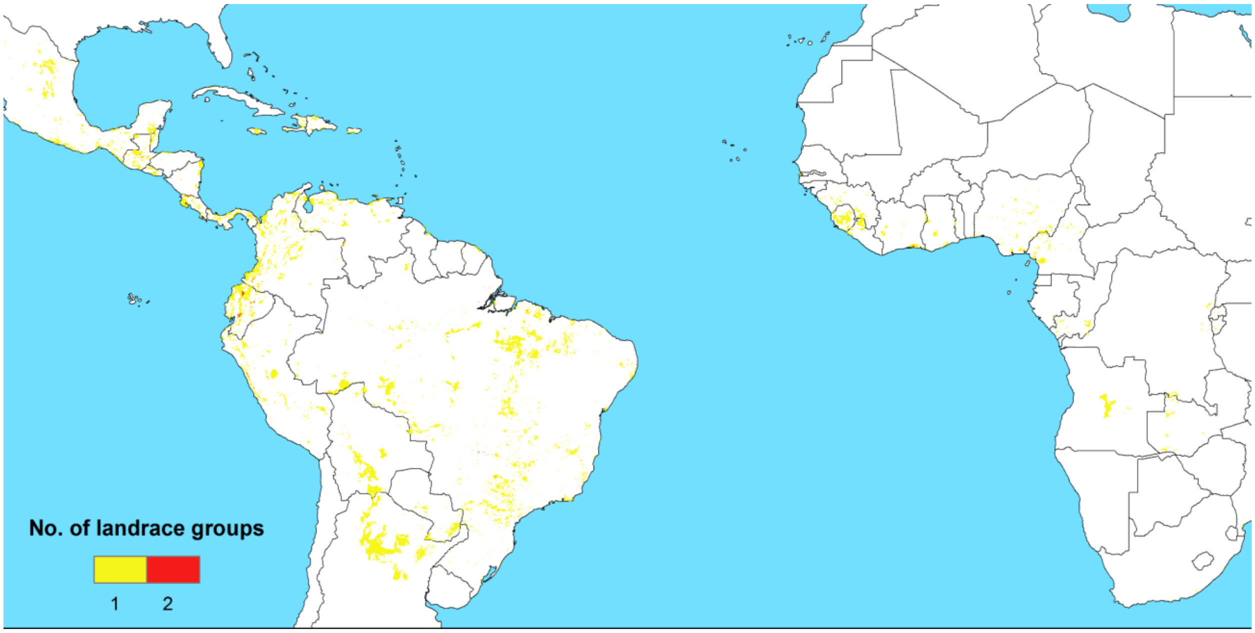


**Supplementary Fig. 21d**: Geographic gaps in the *ex situ* conservation of cassava landrace groups.

*Potato*

Potatoes were domesticated in the Central Andes of South America around 8,000-10,000 years ago from wild *Solanum* L. species [112,113]. Domesticated potatoes later spread in the highland equatorial regions of Colombia and Venezuela, and also in Argentina and Chile.

We tested two major landrace groupings for potato, responding to two recognized taxonomies at species level. The first taxonomy includes the species *Solanum ajanhuiri* Juz. & Bukasov, *Solanum (x) chaucha* Juz. & Bukasov, *Solanum curtilobum* Juz. & Bukasov, *Solanum juzepczukii* Bukasov, *Solanum stenotomum* Juz. & Bukasov, and *Solanum tuberosum* L. [114]. The second, more recent taxonomy is represented by the following species: *S. ajanhuiri*, *S. curtilobum*, *S. juzepczukii*, *Solanum tuberosum* L. subsp. *andigenum* (Juz. & Bukasov) Hawkes (andigena group), and *Solanum tuberosum* L. subsp. *tuberosum* (chilotanum group) [115].

These two taxonomies were evaluated using the published passport data--accession identifiers, geographic coordinates, and species classification--from the CIP database. Both structures are fairly well represented by occurrence data.

Within the classification of Spooner et al. (2007) [115], *S. ajanhuri* was evaluated along with the *andigenum* group comprising *S. tuberosum* subsp. *andigena* and the *chilotanum* group comprising *S. tuberosum* subsp. *tuberosum*. There were, however, insufficient occurrences for robust testing for *S. curtilobum* and *S. juzepczukii*. Within the Hawkes (1990) [114] classification, *S. phureja*, *S. stenotomum*, and *S. tuberosum* were included in the analysis. There were insufficient occurrences for *S. ajanhuiri* and *S. chaucha*. Those species were aggregated in a new class called "others." The Spooner based classification provided higher accuracy (average 90.71%; 49.88% for *ajanhuri;* 78.26% for *andigena*; and 82.58% for *chilotanum*) and was therefore chosen for inclusion in the cross-crop analyses presented here.

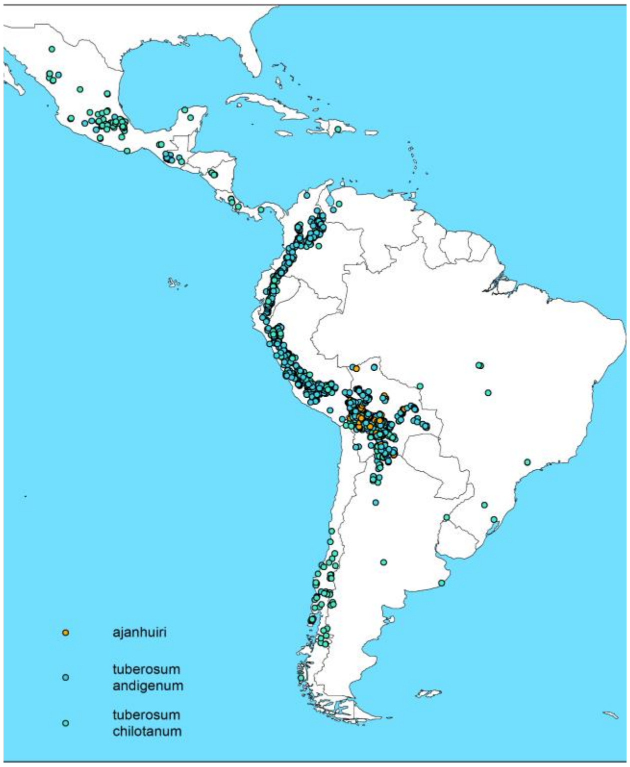


**Supplementary Fig. 22a:** All occurrences within the study region of potato landrace groups.

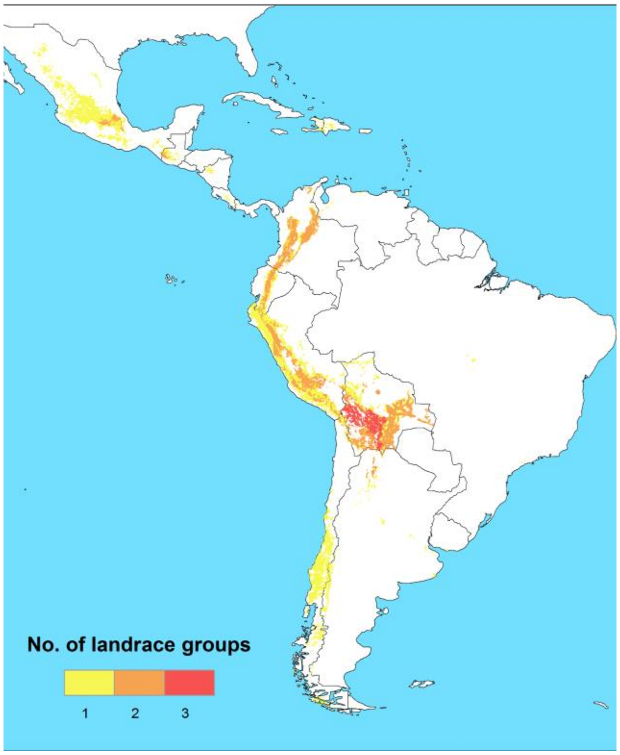


**Supplementary Fig. 22b**: Predicted distributions of potato landrace groups.

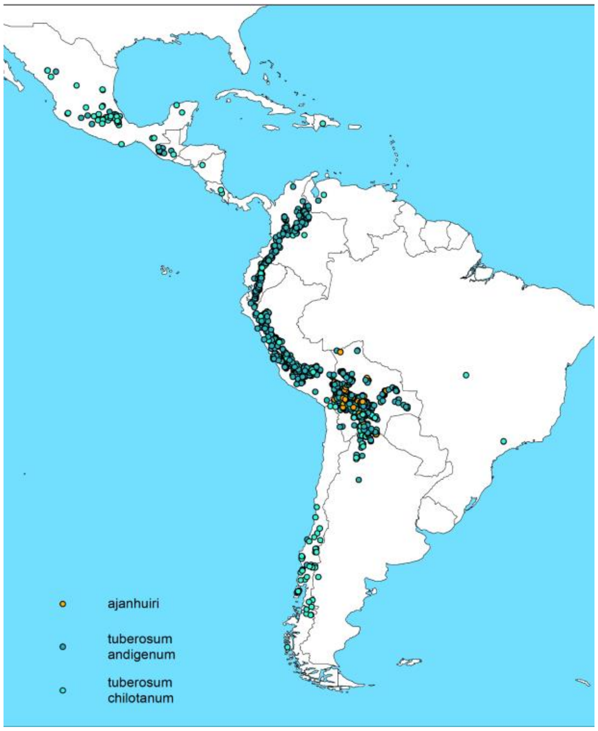


**Supplementary Fig. 22c**: Existing *ex situ* collection occurrences of potato landrace groups.

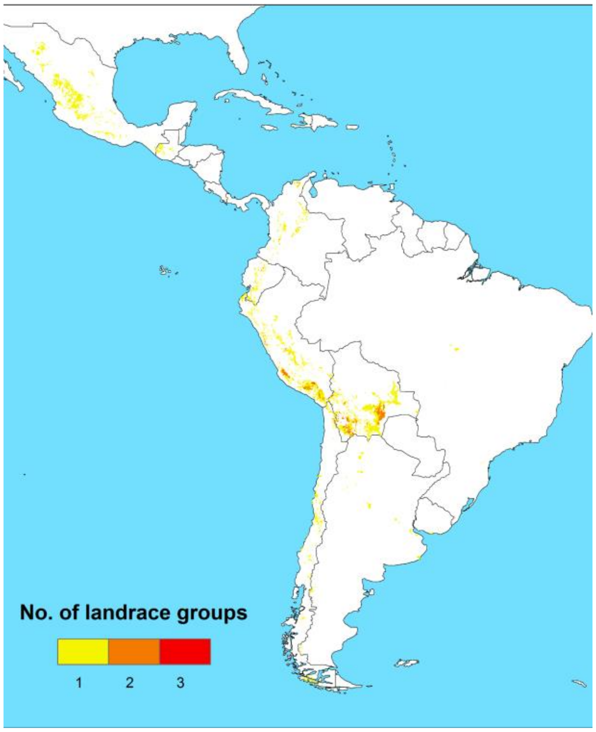


**Supplementary Fig. 22d**: Geographic gaps in the *ex situ* conservation of potato landrace groups.

*Sweetpotato*

Our literature review indicated that sweetpotato, *Ipomoea batatas* (L.) Lam., was likely domesticated independently via two different lineages: a northern lineage in Central America and the Caribbean, and a southern lineage in the northwestern part of South America [116].

Roullier et al. (2011) [116] developed a genetic study using chloroplast markers and identified differences between the northern and southern lineages suggesting two centers of domestication. Based on their work, we classified the passport data provided by CIP and compiled from Genesys, GBIF, and WIEWS (World Information and Early Warning System on Plant Genetic Resources for Food and Agriculture) into the two aforementioned lineages. The northern lineage includes accessions from Panama and countries farther north, and the southern lineage spans from Colombia and Venezuela to the southern countries. Our classification analysis was developed to measure if the two classes responded to bioclimatic and socioeconomic data, and the results showed that the two lineages did have good performance and were explained by ecogeographic characteristics. The average accuracy of the machine learning models was 99.4%, classifying the 98.2% of the occurrences belonging to *Northern lineage*, and 99.6% of the occurrences belonging to *Southern lineage*.

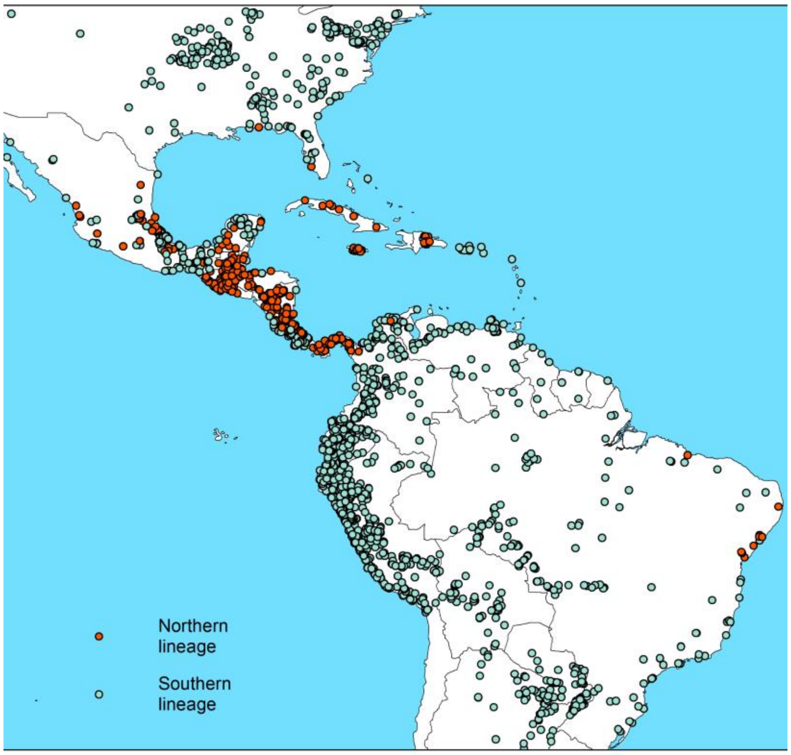


**Supplementary Fig. 23a**: All occurrences within the study region of sweetpotato landrace groups.

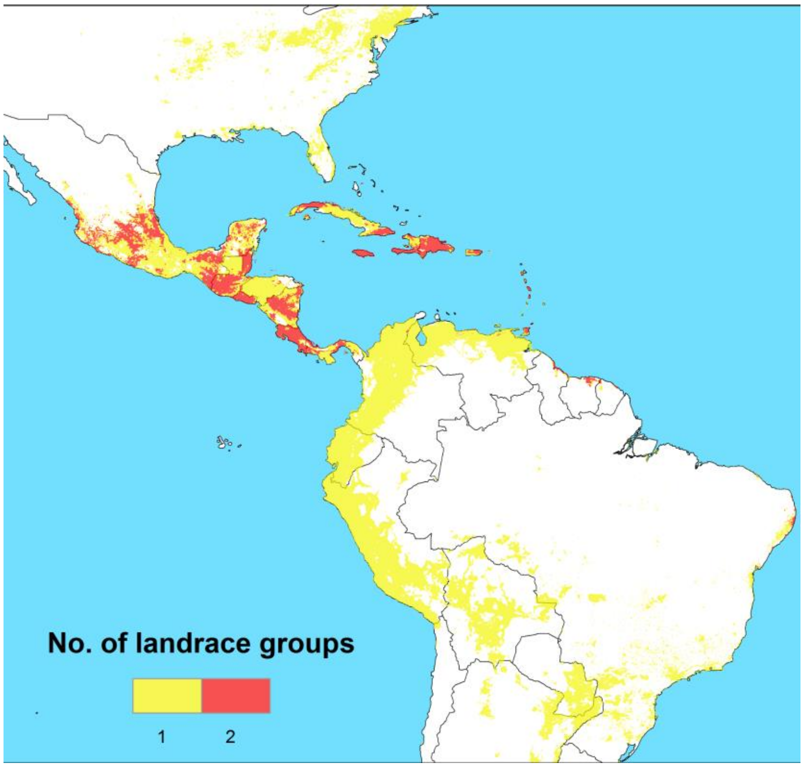


**Supplementary Fig. 23b**: Predicted distributions of sweetpotato landrace groups.

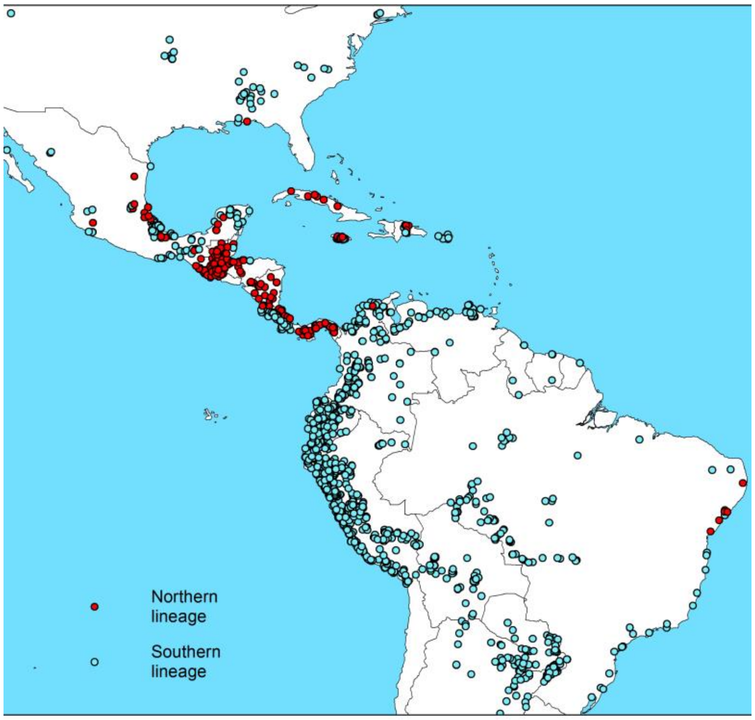


**Supplementary Fig. 23c**: Existing *ex situ* collection occurrences of sweetpotato landrace groups.

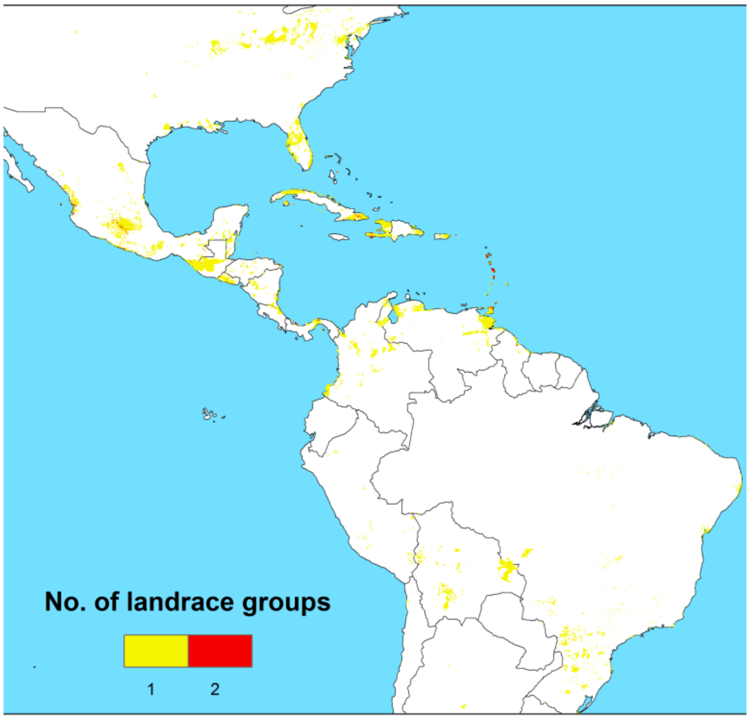


**Supplementary Fig. 23d**: Geographic gaps in the *ex situ* conservation of sweetpotato landrace groups.

*Taro*

Our literature review indicated that taro, *Colocasia esculenta* (L.) Schott, is a root crop distributed in tropical and subtropical areas and mainly grown in tropical China and New Guinea and on many Pacific Islands. It is morphologically diverse with potentially over 10,000 landraces worldwide [117]; however, its origins, diversification, and dispersal are unclear. Genetic diversity within the taro germplasm is known to be greater in accessions from Asia and New Guinea.

Two variants of taros are widely cultivated: *esculenta*, also called dasheen, and *antiquorum*, also called eddoe [118]. Most of the crops cultivated in Asia and the Pacific are dasheen. However, these classifications were very rarely attached to available occurrence data. We therefore performed the modeling and conservation gap analysis for the crop at the species level.

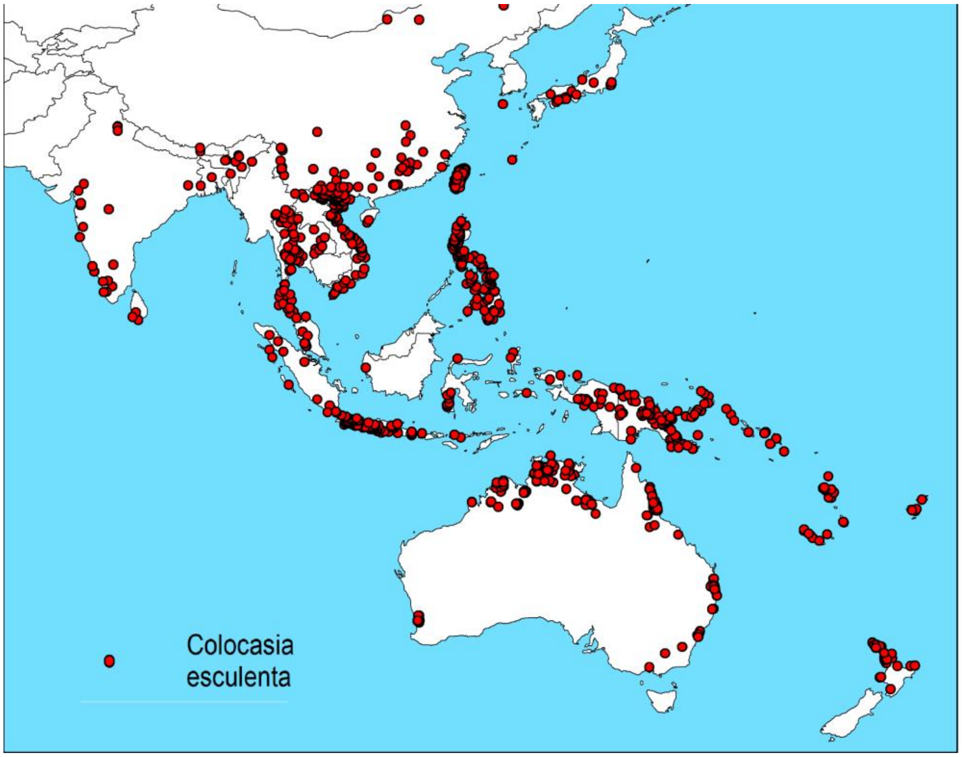


**Supplementary Fig. 24a**, All occurrences within the study region of taro landraces.

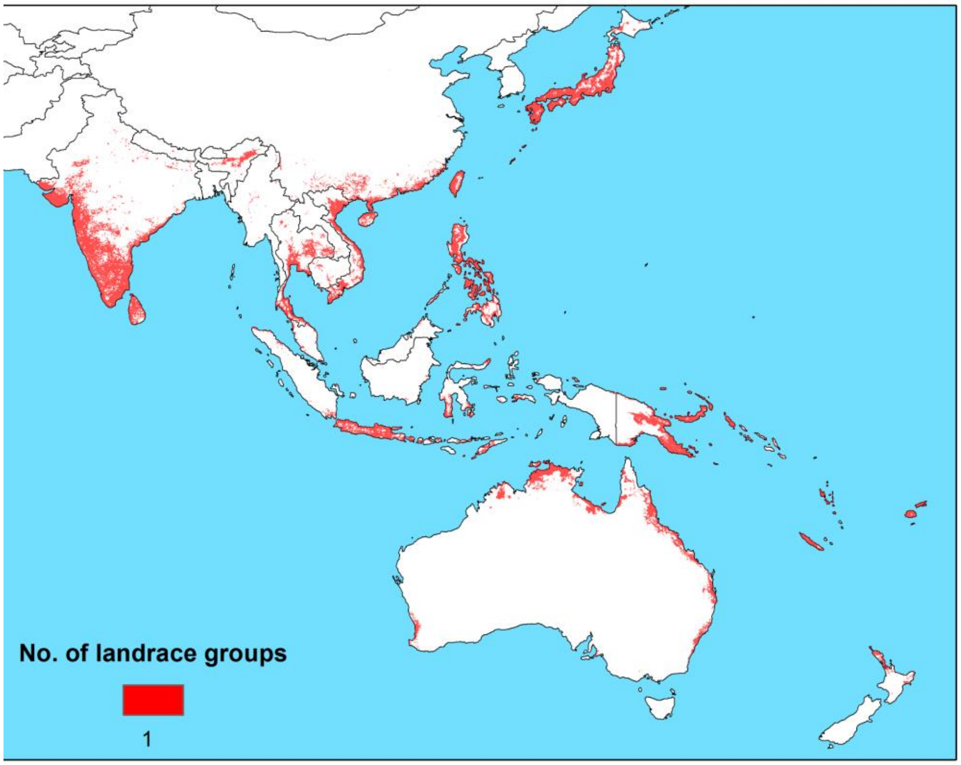


**Supplementary Fig. 24b**: Predicted distributions of taro landraces.

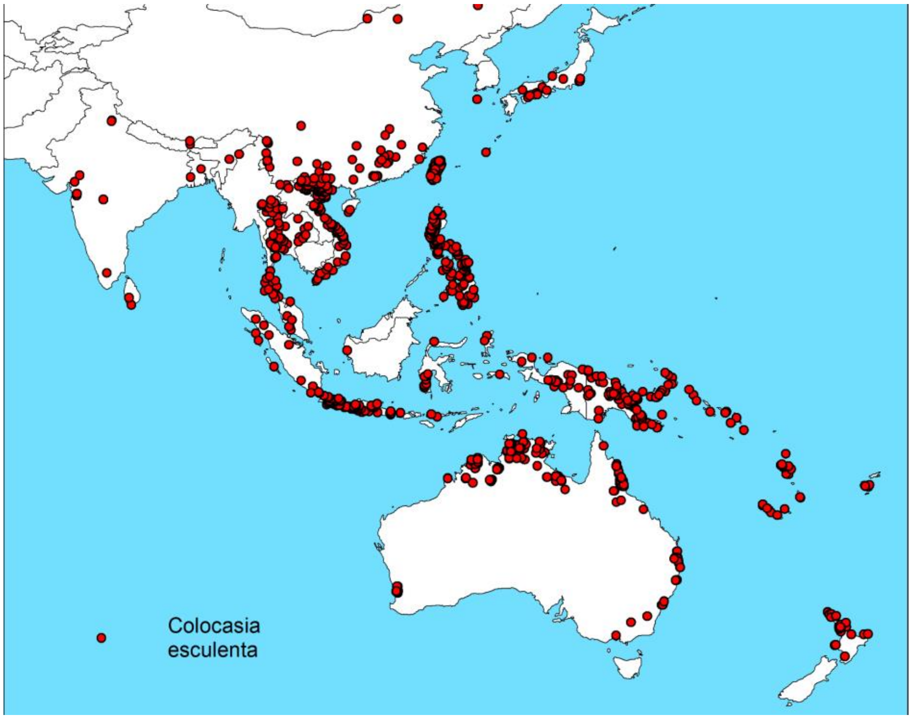


**Supplementary Fig. 24c**: Existing *ex situ* collection occurrences of taro landraces.

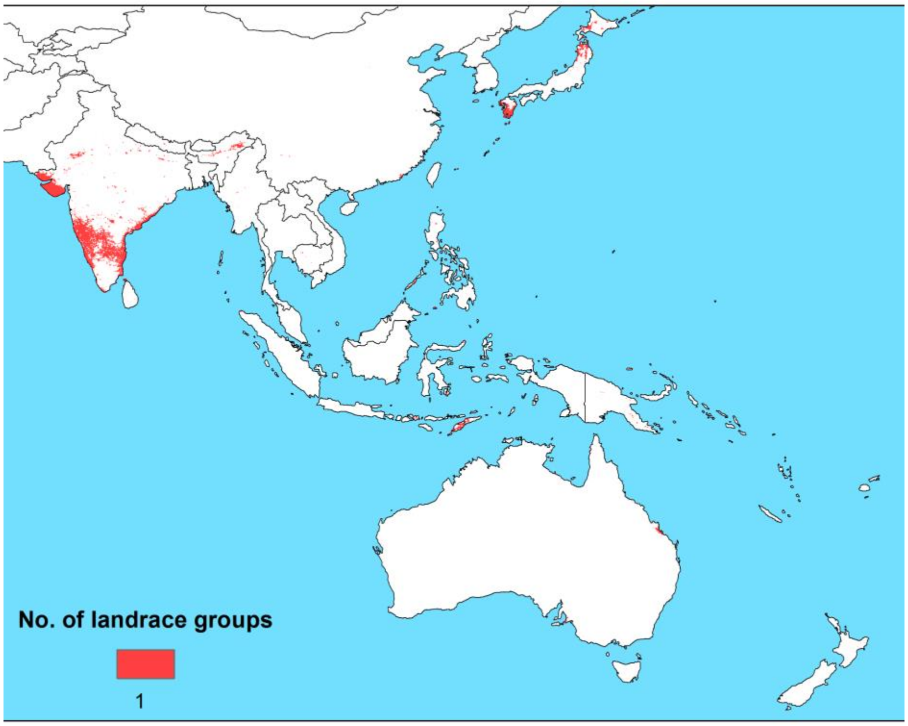


**Supplementary Fig. 24d**: Geographic gaps in the *ex situ* conservation of taro landraces.

*Yams*

According to the Global Strategy for the Conservation and Use of Yam [119], various *Dioscorea* L. spp., collectively called yams, are a staple food for millions of people in about 50 tropical countries. More than 98% of this crop is cultivated in Africa, mostly in Nigeria, Côte d'Ivoire, Ghana, and Benin. Latin America and the Caribbean produce slightly more than 1%, and Asia and Oceania together account for less than 1% of global production. There are at least 11 main food yam species: *Dioscorea alata* L., *Dioscorea bulbifera* L., *Dioscorea cayenensis* Lam., *Dioscorea dumetorum* (Kunth) Pax, *Dioscorea esculenta* (Lour.) Burkill, *Dioscorea japonica* Thunb., *Dioscorea nummularia* Lam., *Dioscorea oppositifolia* L., *Dioscorea pentaphylla* L., *Dioscorea rotundata* Poir., and *Dioscorea trifida* L. f.. The most common species in *ex situ* germplasm collections are *D. alata* and *D. rotundata*, which are well represented across the species' ranges. The other species are less well represented in germplasm collections. The Global Strategy mentions that the 11 main food yam species belong to five taxonomic sections:

- Enantiophyllum: *D. alata*, *D. cayenensis*, *D. japonica*, *D. nummularia*, *D. oppositifolia*, and *D. rotundata*
- Combilium: *D. esculenta*
- Opsophyton: *D. bulbifera*
- Macrogynodium: *D. trifida*
- Lasiophyton: *D. dumetorum* and *D. pentaphylla*

We found a marked imbalance in available occurrences of the 11 yam species. In particularly, there were low numbers of occurrences of species *dumetorum*, *bulbifera*, and *esculenta*. We eventually decided to group the data into the five taxonomic sections and perform the analysis at that classification level. The machine learning models reached an average accuracy of 72.53%, classifying 94.4% of the occurrences belonging to *enantiophyllum*, 34.4% of the occurrences belonging to *lasiophyton*, 18.4% of the occurrences belonging to *opsophyton.* We did not perform the analysis for the combilium group because there was a very low number of occurrences in this group.

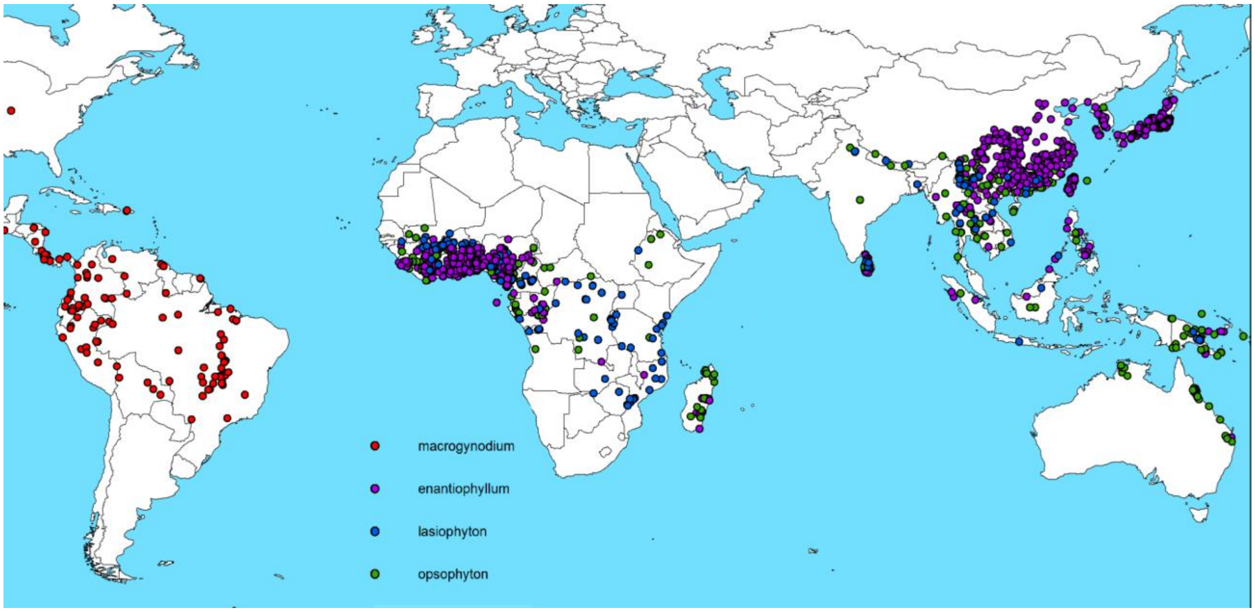


**Supplementary Fig. 25a**: All occurrences within the study region of yam landrace groups.

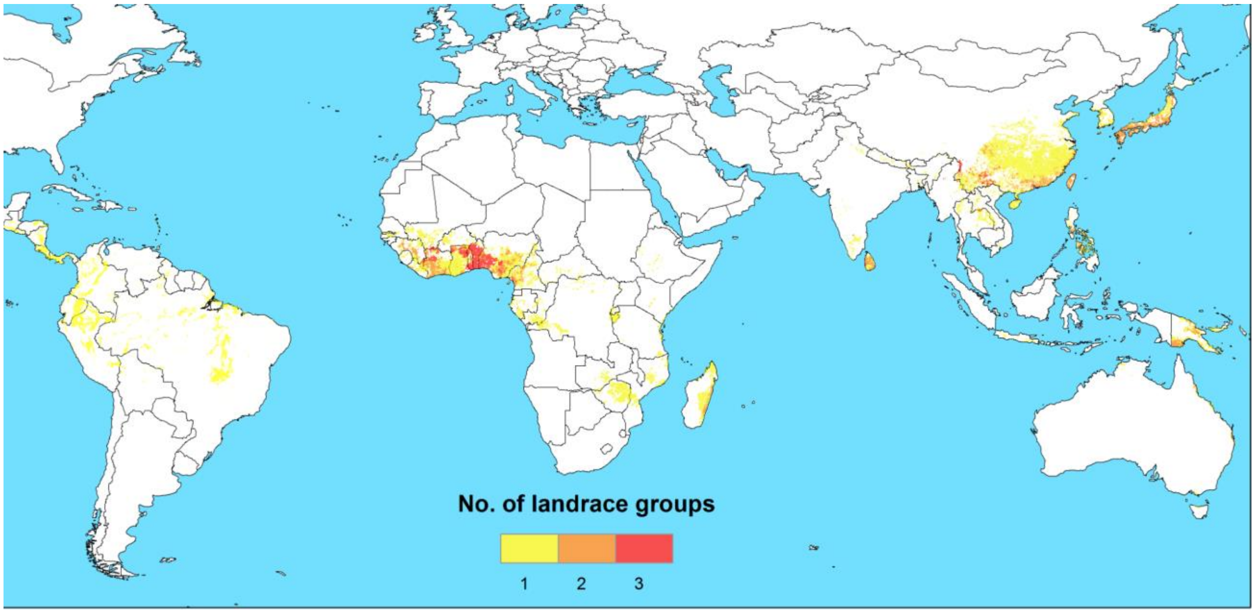


**Supplementary Fig. 25b**: Predicted distributions of yam landrace groups.

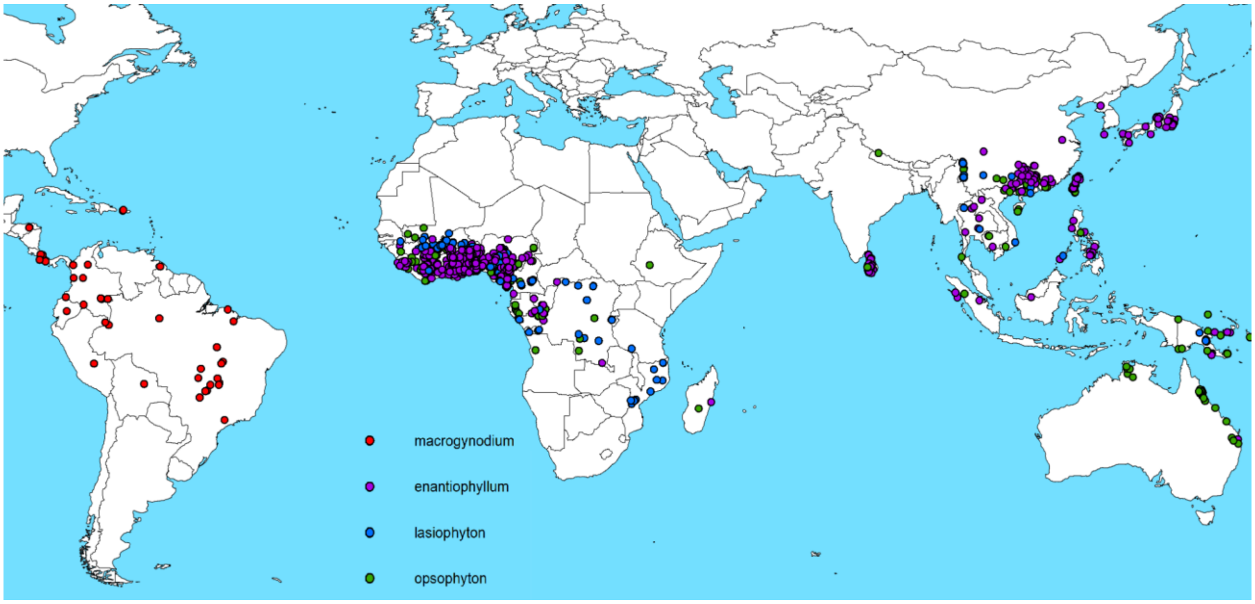


**Supplementary Fig. 25c**: Existing *ex situ* collection occurrences of yam landrace groups.

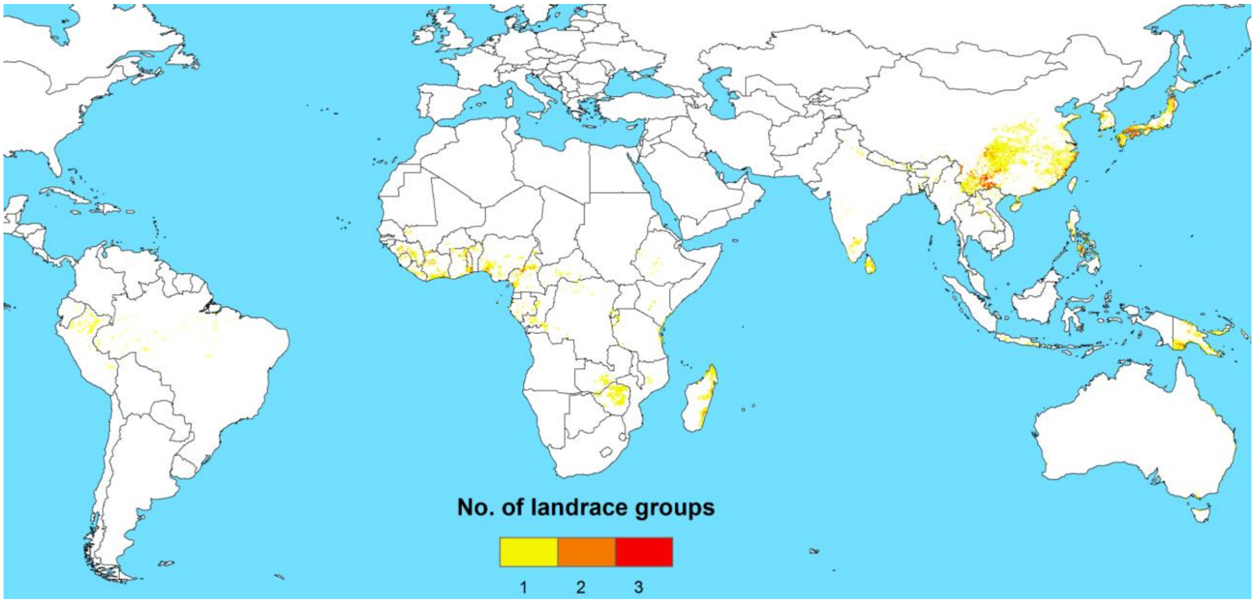


**Supplementary Fig. 25d**: Geographic gaps in the *ex situ* conservation of yam landrace groups.

***Crop importance***

For the values for current crop use in the visualization of current *ex situ* conservation representation (**Fig. 3**), a normalized mean global use score for each crop was produced based on four measures of global aggregate food supply [calories (kcal/capita/day), protein (g/capita/day), fat (g/capita/day), and food weight (g/capita/day)], three measures of total global agricultural production [production quantity (tonnes), harvested area (ha), and production value (gross constant million $USD)], and four measures of global trade [export quantity (tonnes), export value ($USD), import quantity (tonnes), and import value ($USD)], using FAO statistical data [120] data averaged over four recent years (2010-2013) that had data reported for all measures.

Values for crops with multiple pertinent commodity listings (e.g., for grain and for oil separately) were aggregated. Individual food supply values for crops listed within aggregated (general) food supply commodities (i.e., breadfruit in Fruits, Other; chickpea, cowpea, faba bean, grasspea, lentil, and pigeonpea in Pulses, Other; and taro in Roots, Other) were calculated by identifying the quantity of production (tonnes) value for the specific crop and using this value as a factor by which to disaggregate the total general commodity value. Individual production values for crops included in aggregated (general) production commodities (i.e., breadfruit in Fruit, tropical fresh nes; and grasspea in Pulses, nes) were calculated by dividing the general production commodity value equally across its constituent individual crops (for these crops, this process was completed prior to calculating the food supply value described above). An equivalent process was also conducted for trade values for breadfruit (in Fruit, tropical fresh nes). Trade values were not available for cowpea, grasspea, pigeonpea, taro, or yam. African rice (*Oryza glaberrima* Steud.) and Asian rice (*Oryza sativa* L.) were not separately distinguishable in this data, thus measure values for Rice were assigned to both; likewise for finger millet (*Eleusine coracana* (L.) Gaertn.) and pearl millet (*Cenchrus americanus* (L.) Morrone, formerly *Pennisetum glaucum* (L.) R. Br.) (for Millet); and for bread wheat (*Triticum aestivum* L.) and durum wheat (*Triticum turgidum* L. subsp. *durum*) (for Wheat). Reported common bean (Beans) as well as finger millet and pearl millet (Millet) values may actually be inclusive of additional crops which were not included in our conservation analysis.

Combined crop use values were produced for each crop by first normalizing each individual measure value between 0 and 1 across the 25 crops. The four food supply, three agricultural production, and four trade normalized values were then averaged separately for each crop, with a final current crop use value for each crop derived by averaging the mean food supply, production, and trade values. This final current use value is presented on a scale from 0 (low global use) to 100 (high global use).

*Supplementary References*


1. Zohary, D. & Hopf, M. *Domestication of Plants in the Old World: The Origin and Spread of Cultivated Plants in West Asia, Europe, and the Nile Valley* 3rd ed. (Oxford Univ. Press, 2000) pp. 59–69.
2. Komatsuda, T. et al. Six-rowed barley originated from a mutation in a homeodomain-leucine zipper I-class homeobox gene. *Proc Natl Acad Sci U S A.* 104(4), 1424–9 (2007).
3. Russell, J. et al. Exome sequencing of geographically diverse barley landraces and wild relatives gives insights into environmental adaptation. *Nat Genet.* 48(9), 1024–1030 (2016).
4. Crawford, G.W., Lee G-A. Agricultural Origins in the Korean Peninsula. *Antiquity* 77(295), 87–95 (2003).
5. Dai, F. et al. Tibet is one of the centers of domestication of cultivated barley. *Proc Natl Acad Sci U S A.* **109(42),** 16969–16973 (2012).
6. Zeng, X. et al. Origin and evolution of qingke barley in Tibet. *Nat Commun* **9(1)**, 5433 (2018).
7. Nevo, E. et al. Evolution of wild cereals during 28 years of global warming in Israel. *Proc Natl Acad Sci U S A.* **109(9),** 3412–5 (2012).
8. Orabi, J., Backes, G., Wolday, A., Yahyaoui, A., Jahoor, A. The Horn of Africa as a centre of barley diversification and a potential domestication site. *Theor Appl Genet.* **114(6),** 1117–27 (2007).
9. Badr, A. et al. On the Origin and Domestication History of Barley (Hordeum vulgare). *Mol. Biol. Evol.* **17(4),** 499–510 (2000).
10. Bhatty, R.S. The potential of hull-less barley. *Cereal Chem.* **76(5),** 589–599 (1999).
11. Takahashi, R. & Hayashi, J. Linkage study of two complementary genes for brittle rachis in barley. *Ber. Ohara Inst. landw. Biol.* **12,** 99–105 (1964).
12. Takahashi, R. & Yasuda, S. Genetic studies of spring and winter habit in growth in barley. *Ber. Ohara Inst. andw. Biol.* **10,** 245–308 (1956).
13. Hilu, K. W. & De Wet J. M. J. Domestication of Eleusine coracana. *Econ. Bot.* **30,** 199-208 (1976).
14. Phillips, S. M. A survey of the genus Eleusine in Africa. *Kew Bull.* **27,** 251-270 (1972).
15. Hussaini, H., Goodman, M.M., Timothy, D.H. Multivariate analysis and the geographical distribution of the world collection of Finger Millet crops. *Sci.* **17,** 257-263 (1977).
16. De Wet, J.M.J., Prasada, K.E., Brink, D.E. & Mengesha, M. Systematics and Evolution of Eleusine coracana (Gramineae). *Am. J. Bot.* **71(4),** 550-557 (1984).
17. Matsuoka, Y. A single domestication for maize shown by multilocus microsatellite genotyping. *Proc Natl Acad Sci U S A.* **99(9),** 6080–6084 (2002).
18. Van Heerwaarden J. et al. Genetic signals of origin, spread, and introgression in a large sample of maize landraces. *Proc Natl Acad Sci U S A.* **108(3),** 1088–92 (2011).

19. Bedoya, C.A. et al. Genetic diversity and population structure of native maize populations in Latin America and the Caribbean. *PLoS ONE* **12(4),** e0173488 (2017).
20. Mir C. et al. Out of America: tracing the genetic footprints of the global diffusion of maize. *Theor Appl Genet.* **126(11),** 2671–82 (2013).
21. Yadav, O.P., Rai, K.N. Genetic Improvement of Pearl Millet in India. *Agric Res.* **2**, 275–292 (2013).
22. Clark, J.D. The spread of food production in sub-Saharan Africa. *J. Afr. Hist. III* **2,** 211-228 (1962).
23. Portères R. Berceaux agricolos primaries sur le continent africain. *J. Afr. Hist. III* **2,** 195-210 (1962).
24. Harlan, J.R. Agricultural origins: Centres and non-centres. *Sci.* **174,** 468-474 (1971).
25. Marchais, L. Wild pearl millet population (Pennisetum glaucum, Poaceae) integrity in agricultural Sahelian areas. An example from Keita (Niger). *Plant Syst. Evol.***189,** 233-245 (1994).
26. Oumar, I., Mariac, C., Pham, J-L., Vigouroux, Y. Phylogeny and origin of pearl millet (Pennisetum glaucum [L.] R. Br) as revealed by microsatellite loci. *Theor Appl Genet.* **117(4),** 489–97 (2008).
27. IBPGR & ICRISAT. *Descriptors for Pearl Millet.* (International Board for Plant Genetic Resources and International Crops Research Institute for the Semi-Arid Tropics, 1993)
28. Upadhyaya, H.D., Mathur, P.N., & Guarino, L. Global strategy for the ex situ conservation of pearl millet and its wild relatives. https://www.croptrust.org/wp/wp-content/uploads/2014/12/Pearl-Millet-Strategy-FINAL-14May2012.pdf (2012).
29. Upadhyaya, H.D. et al. Characterization and genetic potential of African pearl millet named landraces conserved at the ICRISAT genebank. *Plant Genet Resour.* 1-15 (2016).
30. Brunken, J.N., De Wet, J.M.J., Harlan, J.R. The morphology and domestication of pearl millet. *Econ. Bot.* **31,** 163–174 (1977).
31. Choi, J. Y. et al. The complex geography of domestication of the African rice Oryza glaberrima. *PLoS genetics* **15(3),** e1007414 (2019).
32. Chen, C. et al. Molecular characterization and genetic diversity of different genotypes of Oryza sativa and Oryza glaberrima. *Electron. J. Biotechnol.* **30,** 48-57 (2017).
33. Ndjiondjop, M. N. et al. Assessment of genetic variation and population structure of diverse rice genotypes adapted to lowland and upland ecologies in Africa using SNPs. *Front. Plant Sci.* **9,** 446 (2018).
34. Choi, J. Y. & Purugganan, M. D. Multiple origins but single domestication led to Oryza sativa. *G3 (Bethesda)* **8(3),** 797-803 (2018).
35. Garris, A. J., Tai, T. H., Coburn, J., Kresovich, S., & McCouch, S. Genetic structure and diversity in Oryza sativa L. *Genetics* **169(3),** 1631-1638 (2005).
36. Zhao, K. et al. Genome-wide association mapping reveals a rich genetic architecture of complex traits in Oryza sativa. *Nat commun.* **2(1),** 1-10 (2011).

37. McCouch, S. R. et al. Open access resources for genome-wide association mapping in rice. *Nat commun,* **7(1),** 1-14 (2016).
38. Fuller, D. Q. & Stevens, C. J. (2018). Sorghum domestication and diversification: A current archaeobotanical perspective. In: *Plants and people in the African past* (eds. Mercuri, A. M. , D'Andrea, A. C. , Fornaciari, R. , Höhn, A.) pp. 427–452 (Springer, 2018).
39. Deu, M., Rattunde, F., Chantereau, J. A global view of genetic diversity in cultivated sorghums using a core collection. *Genome* **49(2),** 168–80 (2006).
40. Upadhyaya, H.D. et al. Developing a Mini Core Collection of Sorghum for Diversified Utilization of Germplasm. *Crop Sci.* **49,** 1769-1780 (2009).
41. Harlan, J.R. & De Wet, J.M.J. A Simplified Classification of Cultivated Sorghum1. *Crop Sci.* **12,** 172-176 (1972).
42. Charmet, G. Wheat domestication: lessons for the future. *C.R.-Biol.* **334(3),** 212-220 (2011).
43. Kabbaj, H., et al. Genetic diversity within a global panel of durum wheat (Triticum durum) landraces and modern germplasm reveals the history of alleles exchange. *Front. Plant Sci.* **8,** 1277 (2017).
44. Venske, E., Dos Santos, R. S., Busanello, C., Gustafson, P., De Oliveira, A. C. Bread wheat: a role model for plant domestication and breeding. *Hereditas* **156(1),** 1-11 (2019).
45. Sansaloni, C. et al. Diversity analysis of 80,000 wheat accessions reveals consequences and opportunities of selection footprints. *Nat Commun.* **11(1),** 4572 (2020).
46. Kassie, M. et al. Current situation and future outlooks of the chickpea sub-sector in Ethiopia. ICRISAT working paper. *ICRISAT and EIAR* (2009).
47. Ramirez-Villegas, J. *et al.* A gap analysis modelling framework to prioritize collecting for ex situ conservation of crop landraces. *Divers. Distrib.* **26**, 730–742 (2020).
48. Singh, S., Gepts, P., & Debouck, D. Races of common bean(Phaseolus vulgaris, Fabaceae). *Econ. Bot.* **45,** 379–396 (1991).
49. Bitocchi, E., et al. PNAS Plus: Mesoamerican Origin of the Common Bean (Phaseolus Vulgaris L.) Is Revealed by Sequence Data. *Proc. Natl. Acad. Sci.* (2012).
50. Blair, M. W., Díaz, J. M., Hidalgo, R., Díaz, L. M., & Duque, M. C. Microsatellite characterization of Andean races of common bean (Phaseolus vulgaris L.). *Theor Appl Genet.* **116,** 29–43 (2007).
51. Blair, M. W., Díaz, L. M., Buendía, H. F., & Duque, M. C. Genetic diversity, seed size associations and population structure of a core collection of common beans (Phaseolus vulgaris L.). *Theor Appl Genet.* **119,** 955–972 (2009).
52. Voysest, O. *Variedades de frijol en América Latina y su origen*. (Centro Internacional de Agricultura Tropical, Cali, Colombia, 1983).
53. Delgado-Salinas, A., Turley, T., Richman, A., Lavin, M. Phylogenetic Analysis of the Cultivated and Wild Species of Phaseolus (Fabaceae). *Syst. Bot.* **24(3),** 438 (1999).

54. Gepts, P. *Genetic Resources of Phaseolus Beans : Their Maintenance, Domestication, Evolution, and Utilization*. (Springer, Dordrecht, 1988).
55. Blair, M.W., Diaz, L.M. & Acosta-Gallegos, J.A. Race Structure in the Mexican Collection of Common Bean Landraces. *Crop Sci.* **53,** 1517 (2013).
56. Lobo Burle, M., Fonseca, J.R., Kami, J.A. & Gepts, P. Microsatellite diversity and genetic structure among common bean (Phaseolus vulgaris L.) landraces in Brazil, a secondary center of diversity. *Theor Appl Genet.* **121,** 801–813 (2010).
57. Chacon, M. I., Pickersgill, B., & Debouck, D. G. Domestication patterns in common bean (Phaseolus vulgaris L.) and the origin of the Mesoamerican and Andean cultivated races. *Theor Appl Genet.***110,** 432–444 (2005).
58. Benchimol, L.L., et al. Structure of genetic diversity among common bean (Phaseolus vulgaris L.) varieties of Mesoamerican and Andean origins using new developed microsatellite markers. *Genet. Resour. Crop Evol.* **54,** 1747–1762 (2007).
59. Beebe, S., Rengifo, J., Gaitan, E., Duque, M. C., & Tohme, J. Diversity and Origin of Andean Landraces of Common Bean. *Crop Sci.* **41,** 854 (2001).
60. Gill-Langarica, H.R., Muruaga-Martínez, J.S., Vargas-Vázquez, M.L.P., Rosales-Serna, R. & Mayek-Pérez, N. Genetic diversity analysis of common beans based on molecular markers. *Genet. Mol. Biol.* **34,** 595–605 (2011).
61. Logozzo, G. et al. Analysis of the contribution of mesoamerican and Andean gene pools to European common bean (Phaseolus vulgaris L.) germplasm and strategies to establish a core collection. *Genet. Resour. Crop Evol.* **54,** 1763–1779 (2007).
62. Rodriguez, M. et al. Landscape genetics, adaptive diversity and population structure in Phaseolus vulgaris. *New Phytol.* **209,** 1781–1794 (2016).
63. Duarte, J.M., Santos, J.B. dos & Melo, L.C. Genetic divergence among common bean cultivars from different races based on RAPD markers. *Genet. Mol. Biol.* **22,** 419–426 (1999).
64. Perez-Vega, J.C., Blair, M.W., Monserrate, F. & M, G.L. Evaluation of an Andean common bean reference collection under drought stress Evaluación de una colección de referencia de fríjol andino bajo condiciones de sequía. *Agron Colomb.* **29,** 17–26 (2011).
65. Rosales-Serna, R., Hernandez-Delgado, S., Gonzalez-Paz, M., Acosta-Gallegos, J.A. & Mayek-Perez, N. Genetic Relationships and Diversity Revealed by AFLP Markers in Mexican Common Bean Bred Cultivars. *Crop Science* **45,** 1951 (2005).
66. Becerra-Velasquez, V. L., & Gepts, P. RFLP diversity of common bean (Phaseolus vulgaris) in its centres of origin. *Genome* **37,** 256–263 (1994).
67. Balardin, R.S. & Kelly, J.D. Interaction between Colletotrichum lindemuthianum Races and Gene Pool Diversity in Phaseolus vulgaris. *J. Am. Soc. Hortic. Sci*. **123,** 1038–1047 (1998).
68. Beebe, S. et al. Structure of genetic diversity among common bean landraces of Middle American origin based on correspondence analysis of RAPD. *Crop Sci.* **40,** 264 (2000).

69. Blair, M.W. & Lorigados, S.M. Diversity of Common Bean Landraces, Breeding Lines, and Varieties from Cuba. *Crop Sci.* **56,** 322 (2016).
70. Santalla, M., Rodiño, A. & De Ron, A. Allozyme evidence supporting southwestern Europe as a secondary center of genetic diversity for the common bean. *Theor Appl Genet.* **104,** 934–944 (2002).
71. Paredes, M., Becerra Velasquez, V. & Tay U, J. Inorganic nutritional composition of common bean (Phaseolus vulgaris L.) genotypes race Chile. *Chil. J. Agric. Res.* **69,** 486–495 (2009).
72. Becerra-Velásquez, V., Paredes-Cárcomo, M. & Debouck, D.G. Genetic relationships of common bean (Phaseolus vulgaris L.) race Chile with wild Andean and Mesoamerican germplasm. *Chil. J. Agric. Res.* 71, 3–15 (2011).
73. Blair, M.W., Soler, A. & Cortés, A.J. Diversification and Population Structure in Common Beans (Phaseolus vulgaris L.). *PLoS ONE* **7,** e49488 (2012).
74. Ocampo, C.H., Martin, J.P., Sanchez-Yelamo, M.D., Ortiz, J.M. & Toro, O. Tracing the origin of Spanish common bean cultivars using biochemical and molecular markers. *Genet. Resour. Crop Evol.* **52,** 33–40 (2005).
75. Santalla, M., Rodiño, A. & De Ron, A. Allozyme evidence supporting southwestern Europe as a secondary center of genetic diversity for the common bean. *Theor Appl Genet.* **104,** 934–944 (2002).
76. Asfaw, A., W. Blair, M., Almekinders, C. Genetic Diversity and Population Structure of Common Bean (Phaseolus Vulgaris L.) Landraces from the East African Highlands. *Theor Appl Genet.* **120(1),** 1–12 (2009).
77. Gómez, O.J., Blair, M.W., Frankow-Lindberg, B.E. & Gullberg, U. Molecular and Phenotypic Diversity of Common Bean Landraces from Nicaragua. *Crop Sci.* **44,** 1412 (2004).
78. Kwak, M., Toro, O., Debouck, D.G., Gepts, P. Multiple Origins of the Determinate Growth Habit in Domesticated Common Bean (Phaseolus Vulgaris). *Ann. Bot.* **110(8),** 1573-1580 (2012).
79. Voysest, O., Valencia, M.C., Amezquita, M.C. Genetic Diversity among Latin American Andean and Mesoamerican Common Bean Cultivars. *Crop Sci.* **34(4),** 1100-1110 (1994).
80. Pasquet, R. Allozyme diversity of cultivated cowpea Vigna unguiculata (L.) Walp.. *Theor Appl Genet.* **101,** 211–219 (2000).
81. Koç, S., Orak, A., Tenikecier, H. S., Sağlam, N. Relationship between Seed Yield and Yield Chracteristics in Faba Bean (Vicia faba L.) by GGE-Biplot Analysis. *JoLS.* **12,** 105-110 (2018).
82. Duc, G. Faba bean (Vicia faba L.). *Field Crops Res.* **53(1-3),** 99-109 (1997).
83. Tanno, Ki., Willcox, G. The origins of cultivation of *Cicer arietinum* L. and *Vicia faba* L.: early finds from Tell el-Kerkh, north-west Syria, late 10th millennium B.P. . *Veget Hist Archaeobot* **15,** 197–204 (2006).

84. Muratova, V. Common beans (Vicia faba L.). *Bull. Appl. Bot. and Gen. Plant Breed, Suppl.* **50,** 285 (1931).
85. Hanelt, P. Die infraspezifische Variabilität von Vicia faba L. und ihre Gliederung. *Kulturpflanze* **20(1),** 75-128 (1972).
86. Wang, H. F. et al. Genetic diversity and relationship of global faba bean (Vicia faba L.) germplasm revealed by ISSR markers. *Theor Appl Genet.* **124(5),** 789-797 (2012).
87. Granati E., Bisignano, V., Chiaretti, D., Crin, P., Polignano, G.B. Characterization of Italian and Exotic Lathyrus germplasm for quality traits. *Genet. Resource Crop Evol.* **50,** 273–280 (2003).
88. Gixhari B. & Vrapi, H. Evaluation of Genetic Diversity of Grass Pea (Lathyrus sativum) Genotypes by Morphological Qualitative Traits. *IJGHC* **2(4),** 1050-1056 (2013).
89. Chowdhury, M.A., Slinkard, A.E. Linkage of random amplified polymorphic DNA, isozyme and morphological markers in grasspea (Lathyrus sativus). *J Agric Sci.* **133,** 389-395 (1999).
90. Sammour, R.H., El-Zahar, M.A., Badr S., Tahr, W. Genetic variations in accessions of Lathyrus sativus. *Acta Bot. Croat.* **66(1),** 1–13 (2007).
91. Gixhari, B., Doko, A., Hobdari, V., Vrapi, H. Diversity of grass pea (L. sativum) landraces for sustainable field grass pea breeding in Albania. *IJEES* **6(1),** 81-88 (2016).
92. Krapovickas, A., Gregory, W.C. Taxonomia del genero Arachis (Leguminosae). *Bonpladia* **8,** 1–187 (1994).
93. Gregory, W.C., Krapovickas, A., Gregory, M.P. Structures, variation, evolution and classification in Arachis. In *Advances in legume science* (eds. Summerfield, R.J. & Bunting, A.H.) 469–481 (Royal Botanic Gardens, 1980).
94. Singh, A.K. & Simpson, C.E. Biosystematics and genetic resources. In *The peanut crop: A scientific basis for improvement* (ed. Smartt, J.) 96-137 (Chapman and Hall London, 1994).
95. Barulina, H. Lentils of the USSR and of other countries (English summary). *Bull. Appl. Bot. and Gen. Plant Breed, Suppl.* **40,** 265-304 (1930).
96. Maxted, N. & Ambrose, M. Peas (pisum l.). In: *Plant genetic resources of legumes in the Mediterranean* (eds. Maxted, N. & Bennet, S.J.) 181–190 (Kluwer, Dordrecht, 2000).
97. Saxena, K.B. Genetic Improvement of Pigeonpea- A Review. *Tropical Plant Biol*. **1,** 159–178 (2008).
98. Li, L-F. et al. Origins and Domestication of Cultivated Banana Inferred from Chloroplast and Nuclear Genes. *PLoS ONE* 8(11), e80502 (2013).
99. Perrier, X. et al. Multidisciplinary perspectives on banana (Musa spp.) domestication. *Proc Natl Acad Sci U S A.* **108(28),** 11311–11318 (2011).
100. Lusty, C. Global conservation strategy for Musa (banana and plantain): A consultative document prepared by INIBAP with the collaboration of numerous partners in the Musa research-and-development community. https://www.croptrust.org/wp/wp-content/uploads/2014/12/Musa-Strategy-FINAL-30Jan07.pdf (2006).

101. Zerega, N.J.C., Ragone, D., Motley, T.J. Complex origins of breadfruit (Artocarpus altilis, Moraceae): implications for human migrations in Oceania. *Am J Bot.* **91(5),** 760–766 (2004).
102. Zerega, N.J.C., Ragone, D., Motley, T.J. Systematics and species limits of breadfruit (Artocarpus, Moraceae). *Sys Bot.* **30,** 603–615 (2005).
103. Zerega, N.J.C, Ragone, D., Motley, T.J. Breadfruit origins, diversity, and human-facilitated distribution. In: *Darwin's harvest: new approaches to the origins, evolution, and conservation of crops* (eds. Motley, T.J., Zerega, N.J.C., Cross, H.) 213–238 (Columbia University Press, New York, 2006).
104. Ragone D. *Breadfruit, Artocarpus altilis (Parkinson) Fosberg*. (IPGRI, 1997).
105. Fosberg, F.R. Names in Amaranthus,Artocarpus and Inocarpus. *J. Washington Acad.Sci.* **31(3),** 93-96 (1941).
106. Fosberg, F.R. Introgression in Artocarpus in Micronesia. *Brittonia* **12,** 101-113 (1960).
107. Olsen, K. M., & Schaal, B. A. Evidence on the origin of cassava: phylogeography of Manihot esculenta. *Proc Natl Acad Sci U S A.* **96(10),** 5586-5591 (1999).
108. Isendahl, C. The domestication and early spread of manioc (Manihot esculenta Crantz): a brief synthesis. *Latin American Antiquity* **22(4),** 452-468 (2011).
109. Fregene, M. et al. A molecular genetic map of cassava (Manihot esculenta Crantz). *Theor Appl Genet.* **95(3),** 431-441 (1997).
110. Siqueira, M. V. et al. Genetic characterization of cassava (Manihot esculenta) landraces in Brazil assessed with simple sequence repeats. *Genet. Mol. Biol* **32(1),** 104-110 (2009).
111. Mba, R. E. C. et al. Simple sequence repeat (SSR) markers survey of the cassava (Manihot esculenta Crantz) genome: towards an SSR-based molecular genetic map of cassava. *Theor Appl Genet.* **102(1),** 21-31.
112. Hawkes, J. G. The evolution of cultivated potatoes and their tuber-bearing wild relatives. *Die Kulturpflanze* 36(1), 189-208 (1988).
113. Hardigan, M. A. et al. Genome diversity of tuber-bearing Solanum uncovers complex evolutionary history and targets of domestication in the cultivated potato. *Proc Natl Acad Sci U S A.* **114(46),** E9999-E10008 (2017).
114. Hawkes, J.G. *The potato: Evolution, biodiversity and genetic resources.* (Belhaven Press, 1990) pp. 259.
115. Spooner, D. M. et al. Extensive simple sequence repeat genotyping of potato landraces supports a major reevaluation of their gene pool structure and classification. *Proc Natl Acad Sci U S A.* **104(49),** 19398-19403 (2007).
116. Roullier, C., Rossel, G., Tay, D., Mckey, D., Lebot, V. Combining chloroplast and nuclear microsatellites to investigate origin and dispersal of New World sweet potato landraces. *Mol. Ec.* **20(19),** 3963-3977 (2011).
117. Miyasaka S.C. et al. Diversity in horticultural plants. *Sustainable Development and Biodiversity.* **22(7),** 191-209 (2019).

118. Deo, P.C., Tyagi, A.P., Taylor, M., Becker, D.K., Harding, R.M. Improving Taro (Colocasia esculenta var. esculenta) production using biotechnological approaches. *S Pac J Nat Sci*. **27,** 6-13 (2009).
119. Global Crop Diversity Trust. Towards a global strategy for the conservation and use of yam. https://cdn.croptrust.org/wp/wp-content/uploads/2017/07/Yam-conservation-strategy-Part-1-and-2.pdf (2010).
120. Food and Agriculture Organization of the United Nations. FAOSTAT. https://www.fao.org/faostat/en (2019).
