# Supplementary material for: State of ex situ conservation of landrace groups of 25 major crops
Source: Nat Plants. 2022 May 9;8(5):491–9. doi: 10.1038/s41477-022-01144-8 (PMC9122826; doi:10.1038/s41477-022-01144-8)
Supplement: Supplementary file 2 — Reporting Summary [file 41477_2022_1144_MOESM2_ESM.pdf]

nature portfolio

Corresponding author(s): Colin Khoury

Last updated by author(s): 2022_3_19

# Reporting Summary

Nature Portfolio wishes to improve the reproducibility of the work that we publish. This form provides structure for consistency and transparency in reporting. For further information on Nature Portfolio policies, see our Editorial Policies and the Editorial Policy Checklist.

## Statistics

For all statistical analyses, confirm that the following items are present in the figure legend, table legend, main text, or Methods section.

| n/a | Confirmed | |
|---|---|---|
| ☐ | ☒ | The exact sample size (*n*) for each experimental group/condition, given as a discrete number and unit of measurement |
| ☒ | ☐ | A statement on whether measurements were taken from distinct samples or whether the same sample was measured repeatedly |
| ☒ | ☐ | The statistical test(s) used AND whether they are one- or two-sided<br>*Only common tests should be described solely by name; describe more complex techniques in the Methods section.* |
| ☐ | ☒ | A description of all covariates tested |
| ☒ | ☐ | A description of any assumptions or corrections, such as tests of normality and adjustment for multiple comparisons |
| ☐ | ☒ | A full description of the statistical parameters including central tendency (e.g. means) or other basic estimates (e.g. regression coefficient) AND variation (e.g. standard deviation) or associated estimates of uncertainty (e.g. confidence intervals) |
| ☒ | ☐ | For null hypothesis testing, the test statistic (e.g. *F*, *t*, *r*) with confidence intervals, effect sizes, degrees of freedom and *P* value noted<br>*Give P values as exact values whenever suitable.* |
| ☒ | ☐ | For Bayesian analysis, information on the choice of priors and Markov chain Monte Carlo settings |
| ☐ | ☒ | For hierarchical and complex designs, identification of the appropriate level for tests and full reporting of outcomes |
| ☒ | ☐ | Estimates of effect sizes (e.g. Cohen's *d*, Pearson's *r*), indicating how they were calculated |

*Our web collection on statistics for biologists contains articles on many of the points above.*

## Software and code

Policy information about availability of computer code

| | |
|---|---|
| Data collection | No software or code was used to collect data. Data collection is fully described in the Methods section. |
| Data analysis | We created custom code which is permanently available at: https://github.com/CIAT-DAPA/gap_analysis_landraces. A code availability statement is included in the Methods section. |

For manuscripts utilizing custom algorithms or software that are central to the research but not yet described in published literature, software must be made available to editors and reviewers. We strongly encourage code deposition in a community repository (e.g. GitHub). See the Nature Portfolio guidelines for submitting code & software for further information.

## Data

Policy information about availability of data

All manuscripts must include a data availability statement. This statement should provide the following information, where applicable:

- Accession codes, unique identifiers, or web links for publicly available datasets
- A description of any restrictions on data availability
- For clinical datasets or third party data, please ensure that the statement adheres to our policy

Occurrence data - For ex situ conservation records, occurrences marked as landraces were retrieved from two major online databases: the Genesys Plant Genetic Resources portal32 and the World Information and Early Warning System on Plant Genetic Resources for Food and Agriculture (WIEWS) of the Food and Agriculture Organization of the United Nations33. Occurrences were also obtained directly from individual international genebank information systems: AfricaRice, the International Transit Centre and Musa Germplasm Information System of Bioversity International34, CePaCT, CIAT, CIMMYT, CIP, ICARDA, ICRISAT, IITA, and IRRI, as well as from the USDA Genetic Resources Information Network (GRIN)–Global35 and the Comisión Nacional para el Conocimiento y Uso de la Biodiversidad (CONABIO)36. Occurrences were compiled from the Global Biodiversity Information Facility (GBIF)37, with 'living specimen' records classified as ex situ conservation

records and the remaining serving as reference sightings for use in distribution modeling. Reference occurrences were also drawn from published literature (Supplementary Dataset 2 [Supplementary References]).

Spatial predictor data - We compiled and calculated spatially explicit gridded information for 50 potential environmental and cultural predictors of landrace distributions, including climatic, topographic, evolutionary history, and socioeconomic variables (Supplementary Dataset 1 [Supplementary Table 3])13. For climate data, we gathered or derived 39 variables, from WorldClim version 238 and Environmental Rasters for Ecological Modeling (ENVIREM)39. We included elevation from the Shuttle Radar Topography Mission (SRTM) dataset of the CGIAR-Consortium on Geospatial Information portal40,41. Two crop evolutionary history proxies were included: distance to human settlements before the year CE 150042, and distance to known wild progenitor populations13. The eight socioeconomic variables included population density43, distance to navigable rivers44, percentage of the area under irrigation45, population accessibility46,47, geographic distributions of ethnic or cultural groups48, and crop harvested area, production quantity, and yield49. All predictor data were scaled to 2.5 arc-minute resolution with World Geodetic System (WGS) 84 as a datum. Occurrence data, including spatial predictor variable results (at 2.5 arc minute resolution) for each occurrence (available at Supplementary Dataset 3). A global spatial predictor dataset (2.5 arc minute resolution, all 50 variables) (available at Supplementary Dataset 4).

Crop landrace group classification data - For each crop, we  conducted an extensive literature review to identify recognized infraspecific groups with distinct morphological, physiological, chemical, genetic, nomenclatural, or other characteristics that could be tested for environmental and cultural associations (Supplementary Dataset 1 [Supplementary Table 1], Supplementary Dataset 2 [Supplementary Methods and References]). The nature of these groups varied by crop, and included genepools, races, genetic clusters, and geographic or environmental groupings. Crops often had more than one proposed grouping or classification.

# Field-specific reporting

Please select the one below that is the best fit for your research. If you are not sure, read the appropriate sections before making your selection.

☐ Life sciences ☐ Behavioural & social sciences ☒ Ecological, evolutionary & environmental sciences

For a reference copy of the document with all sections, see nature.com/documents/nr-reporting-summary-flat.pdf

# Ecological, evolutionary & environmental sciences study design

All studies must disclose on these points even when the disclosure is negative.

**Study description**

Literature review and expert consultation were conducted to understand the various possible infraspecific genetic structures within assessed crops. Occurrence data from authoritative databases and published literature were compiled for these structures. This dataset was used to test the power of ecogeographic (spatial) information in distinguishing the various landrace groups within a crop (using the 50 ecogeographic spatial predictors, compiled in Ramirez-Villegas et al. 2020). Once the infraspecific structure with highest accuracy (ecogeographic signal) was identified, a total occurrence dataset for the landraces of each crop was compiled, with each occurrence attributed to a specific landrace group. These groups were then spatially modeled. Models were then subjected to the conservation gap analysis - essentially a comparison of these models to previous locations where germplasm for these landraces has been collected and is now conserved in genebanks. This gap analysis used three main approaches in determining conservation gaps- connectivity, accessibility, and environmental difference.

**Research sample**

Two main research samples are identified:

Crop landrace group sample (an occurrence dataset with crop landrace group information attributed) - This dataset was used to test the power of ecogeographic signal in distinguishing the various landrace groups within a crop (using the 50 ecogeographic spatial predictors). This research sample was compiled by conducting an extensive literature review to identify recognized infraspecific groups with distinct morphological, physiological, chemical, genetic, nomenclatural, or other characteristics that could be tested for environmental and cultural associations (Supplementary Dataset 1 [Supplementary Table 1], Supplementary Dataset 2 [Supplementary Methods and References]). The nature of these groups varied by crop, and included genepools, races, genetic clusters, and geographic or environmental groupings.

The total occurrence dataset for each crop - For ex situ conservation records, occurrences marked as landraces were retrieved from two major online databases: the Genesys Plant Genetic Resources portal32 and the World Information and Early Warning System on Plant Genetic Resources for Food and Agriculture (WIEWS) of the Food and Agriculture Organization of the United Nations33. Occurrences were also obtained directly from individual international genebank information systems: AfricaRice, the International Transit Centre and Musa Germplasm Information System of Bioversity International34, CePaCT, CIAT, CIMMYT, CIP, ICARDA, ICRISAT, IITA, and IRRI, as well as from the USDA Genetic Resources Information Network (GRIN)–Global35 and the Comisión Nacional para el Conocimiento y Uso de la Biodiversidad (CONABIO)36. Occurrences were compiled from the Global Biodiversity Information Facility (GBIF)37, with 'living specimen' records classified as ex situ conservation records and the remaining serving as reference sightings for use in distribution modeling. Reference occurrences were also drawn from published literature (Supplementary Dataset 2 [Supplementary References]). Duplicated observations within or between data sources were eliminated, with a preference to utilize the most original data. Coordinates were corrected or removed when latitude and longitude were equal to zero or inverted, located in water bodies or in the wrong country, or had poor resolution (< 2 decimal places). Occurrences were clipped to study areas per crop. The complete occurrence dataset is available in Supplementary Dataset 3. After performing the steps described in a, all other occurrence data records not attributed to landrace groups were predicted. This complete dataset was then used for crop landrace distribution modeling and conservation gap analysis

**Sampling strategy**

Crop landrace group structures were tested using 15-fold cross-validation with 80% training and 20% testing. We accepted a given classification if each of its components was predicted with an average cross-validated accuracy of at least 80%. Spatial distribution models were fitted through five-fold (K = 5) cross-validation with 80% training and 20% testing. For each fold, we calculated the area under the receiving operating characteristic curve (AUC), sensitivity, specificity, and Cohen's kappa as measures of model performance. To create a single prediction that represents the probability of occurrence for the landrace group, we computed the

median across K models. Geographic areas in the form of pixels with probability values above the maximum sum of sensitivity and specificity were treated as the final area of predicted presence.

Data collection

Occurrence data - For ex situ conservation records, occurrences marked as landraces were retrieved from two major online databases: the Genesys Plant Genetic Resources portal32 and the World Information and Early Warning System on Plant Genetic Resources for Food and Agriculture (WIEWS) of the Food and Agriculture Organization of the United Nations33. Occurrences were also obtained directly from individual international genebank information systems: AfricaRice, the International Transit Centre and Musa Germplasm Information System of Bioversity International34, CePaCT, CIAT, CIMMYT, CIP, ICARDA, ICRISAT, IITA, and IRRI, as well as from the USDA Genetic Resources Information Network (GRIN)–Global35 and the Comisión Nacional para el Conocimiento y Uso de la Biodiversidad (CONABIO)36. Occurrences were compiled from the Global Biodiversity Information Facility (GBIF)37, with 'living specimen' records classified as ex situ conservation records and the remaining serving as reference sightings for use in distribution modeling. Reference occurrences were also drawn from published literature (Supplementary Dataset 2 [Supplementary References]). Duplicated observations within or between data sources were eliminated, with a preference to utilize the most original data. Coordinates were corrected or removed when latitude and longitude were equal to zero or inverted, located in water bodies or in the wrong country, or had poor resolution (< 2 decimal places). Occurrences were clipped to study areas per crop. The complete occurrence dataset is available in Supplementary Dataset 3.

Spatial predictors - We compiled and calculated spatially explicit gridded information for 50 potential environmental and cultural predictors of landrace distributions, including climatic, topographic, evolutionary history, and socioeconomic variables (Supplementary Dataset 1 [Supplementary Table 3])13. For climate data, we gathered or derived 39 variables, from WorldClim version 238 and Environmental Rasters for Ecological Modeling (ENVIREM)39. We included elevation from the Shuttle Radar Topography Mission (SRTM) dataset of the CGIAR-Consortium on Geospatial Information portal40,41. Two crop evolutionary history proxies were included: distance to human settlements before the year CE 150042, and distance to known wild progenitor populations13. The eight socioeconomic variables included population density43, distance to navigable rivers44, percentage of the area under irrigation45, population accessibility46,47, geographic distributions of ethnic or cultural groups48, and crop harvested area, production quantity, and yield49. All predictor data were scaled to 2.5 arc-minute resolution with World Geodetic System (WGS) 84 as a datum. Extended descriptions of the sources and their justification for inclusion are provided in Ramirez-Villegas et al. (2020)13. For both datasets, data are provided in spreadsheets (i.e. Microsoft Excel).

Crop landrace group classification - for each crop, we conducted an extensive literature review to identify recognized infraspecific groups with distinct morphological, physiological, chemical, genetic, nomenclatural, or other characteristics that could be tested for environmental and cultural associations (Supplementary Dataset 1 [Supplementary Table 1], Supplementary Dataset 2 [Supplementary Methods and References]). The nature of these groups varied by crop, and included genepools, races, genetic clusters, and geographic or environmental groupings. Crops often had more than one proposed grouping or classification.

Timing and spatial scale

Occurrence data covers all time periods but the vast majority of data is from the past 50 years. Worldclim predictor data is annual average from 1970-2000; Envirem data is annual average from 1960-1990 (current); other predictors described in Ramirez-Villegas et al. 2020 refer to current conditions (e.g., road network, ethnic groups). Spatial scale is region and crop dependent; in total across this study, spatial scale is global, whereas the spatial resolution is 2.5 arc-min. Data collection for this work initiated in January 2017 and concluded in July 2021. We generally compiled this data crop by crop in collaboration with crop experts and often during in person collaborative workshops at each of the international research centers.

Data exclusions

Crop landrace group (infraspecific structure) data that was outperformed by other competing proposed structures was not used in the final combined analysis; results for these alternative structures are provided in Supplementary Table 2.

Reproducibility

All data is available through open access repositories; all code is available on Github (https://github.com/CIAT-DAPA/gap_analysis_landraces).

Randomization

Data: Occurrence data from authoritative databases and published literature were compiled for landrace group structures. This dataset was used to test the power of ecogeographic (spatial) information in distinguishing the various landrace groups within a crop (using the 50 ecogeographic spatial predictors, compiled in Ramirez-Villegas et al. 2020). Once the infraspecific structure with highest accuracy (ecogeographic signal) was identified, a total occurrence dataset for the landraces of each crop was compiled, with each occurrence attributed to a specific landrace group. We then built and tested classification models to determine how well the proposed groups could be predicted and distinguished based on spatial predictors, drawing from the occurrence database and training datasets compiled from the literature review. A random forest53, a support vector machine54, the K-nearest neighbor (KNN) algorithm55, and artificial neural networks56 were used to determine classification performance. The response variable was the group to which a given occurrence was assigned, whereas the explanatory variables were the spatial predictors. Models were combined into an ensemble using the mode—that is, the most frequent predicted value among the models—and tested using 15-fold cross-validation with 80% training and 20% testing (samples drawn at random). We accepted a given classification if each of its components was predicted with an average cross-validated accuracy of at least 80%. In the case of multiple classification proposals per crop, we selected the one with the best overall performance. Finally, we used the trained models to predict the corresponding group for occurrences missing such information. All landrace groups for all crops are provided in Supplementary Dataset 1 [Supplementary Table 2], with the best-performing groups identified.

Distribution modelling: To predict the probability of geographic occurrence for each landrace group within each crop, we generated MaxEnt models57,58 using the 'maxnet' R package59. Group-specific spatial predictors were selected using a combination of the variance inflation factor (VIF) and a principal component analysis (PCA) to control for excessive model complexity and variable collinearity60. We removed variables that did not contribute significantly to the variance in the PCA, defined as contributing less than 15% to the first component, and we further discarded variables with a VIF > 1061. The predictors and whether they were selected for the modeling of each landrace group are presented in Supplementary Dataset 1 (Supplementary Table 4). We generated a random sample of pseudo-absences as background points in areas that (a) were within the same ecological land units62 as the occurrence points, (b) were deemed potentially suitable according to a support vector machine classifier that uses all occurrences and predictor variables, and (c) were further than 5 km from any occurrence63. The number of pseudo-absences generated per crop group was ten times its number of unique occurrences. MaxEnt models were fitted through five-fold (K = 5) cross-validation with 80% training and 20% testing (with samples for these splits drawn at random each time). For each fold, we calculated the area under the receiving

operating characteristic curve (AUC), sensitivity, specificity, and Cohen's kappa as measures of model performance. To create a single prediction that represents the probability of occurrence for the landrace group, we computed the median probability across K models. Geographic areas in the form of pixels with probability values above the maximum sum of sensitivity and specificity were treated as the final area of predicted presence13.

Gap analysis validation - Spatial ex situ conservation gaps were determined from the conservation gap scores using a cross-validation procedure to derive a threshold for each score. We created synthetic gaps by removing existing genebank occurrences in five randomly chosen circular areas with a 100 km radius within the distribution model. We then tested whether these artificial gaps could be predicted by our gap analysis, identifying the threshold value of each score that would maximize the prediction of these synthetic gaps. Performance for each of the five gap areas was assessed using AUC, sensitivity, and specificity. The average cross-area threshold value was calculated for each score to discern pixels with a high likelihood of finding ex situ conservation gaps and which thus were higher priority for further field sampling. These were pixels with combined gap scores above the threshold, assigned a value of 1, as opposed to the relatively well-conserved areas below the threshold, which were assigned a value of 0.

Blinding

Blinding was not relevant to this study. All relevant data for pertinent crop landraces was acquired from authoritative databases and from published literature. These were used to test classification models, build distribution models, and perform gap analysis and validation based on statistical power/significance, as described above

Did the study involve field work? ☐ Yes ☒ No

# Reporting for specific materials, systems and methods

We require information from authors about some types of materials, experimental systems and methods used in many studies. Here, indicate whether each material, system or method listed is relevant to your study. If you are not sure if a list item applies to your research, read the appropriate section before selecting a response.

## Materials & experimental systems

| n/a | Involved in the study | |
|---|---|---|
| ☒ | ☐ | Antibodies |
| ☒ | ☐ | Eukaryotic cell lines |
| ☒ | ☐ | Palaeontology and archaeology |
| ☒ | ☐ | Animals and other organisms |
| ☒ | ☐ | Human research participants |
| ☒ | ☐ | Clinical data |
| ☒ | ☐ | Dual use research of concern |

## Methods

| n/a | Involved in the study | |
|---|---|---|
| ☒ | ☐ | ChIP-seq |
| ☒ | ☐ | Flow cytometry |
| ☒ | ☐ | MRI-based neuroimaging |
